# Precision and accuracy of single-molecule FRET measurements—a multi-laboratory benchmark study

**DOI:** 10.1038/s41592-018-0085-0

**Published:** 2018-08-31

**Authors:** Björn Hellenkamp, Sonja Schmid, Olga Doroshenko, Oleg Opanasyuk, Ralf Kühnemuth, Soheila Rezaei Adariani, Benjamin Ambrose, Mikayel Aznauryan, Anders Barth, Victoria Birkedal, Mark E. Bowen, Hongtao Chen, Thorben Cordes, Tobias Eilert, Carel Fijen, Christian Gebhardt, Markus Götz, Giorgos Gouridis, Enrico Gratton, Taekjip Ha, Pengyu Hao, Christian A. Hanke, Andreas Hartmann, Jelle Hendrix, Lasse L. Hildebrandt, Verena Hirschfeld, Johannes Hohlbein, Boyang Hua, Christian G. Hübner, Eleni Kallis, Achillefs N. Kapanidis, Jae-Yeol Kim, Georg Krainer, Don C. Lamb, Nam Ki Lee, Edward A. Lemke, Brié Levesque, Marcia Levitus, James J. McCann, Nikolaus Naredi-Rainer, Daniel Nettels, Thuy Ngo, Ruoyi Qiu, Nicole C. Robb, Carlheinz Röcker, Hugo Sanabria, Michael Schlierf, Tim Schröder, Benjamin Schuler, Henning Seidel, Lisa Streit, Johann Thurn, Philip Tinnefeld, Swati Tyagi, Niels Vandenberk, Andrés Manuel Vera, Keith R. Weninger, Bettina Wünsch, Inna S. Yanez-Orozco, Jens Michaelis, Claus A. M. Seidel, Timothy D. Craggs, Thorsten Hugel

**Affiliations:** 1 Institute of Physical Chemistry, University of Freiburg, Freiburg im Breisgau, Germany; 2 Engineering and Applied Sciences, Columbia University, New York, NY USA; 3 Department of Bionanoscience, Kavli Institute of Nanoscience Delft, Delft University of Technology, Delft, the Netherlands; 4 Molecular Physical Chemistry, Heinrich-Heine-Universität Düsseldorf, Düsseldorf, Germany; 5 Department of Physics and Astronomy, Clemson University, Clemson, SC USA; 6 Department of Chemistry, University of Sheffield, Sheffield, UK; 7 Interdisciplinary Nanoscience Center (iNANO) and Department of Chemistry, Aarhus University, Aarhus, Denmark; 8 Physical Chemistry, Department of Chemistry, Nanosystems Initiative Munich (NIM), Center for Integrated Protein Science Munich (CiPSM) and Center for Nanoscience (CeNS), Ludwig-Maximilians-Universität München, Munich, Germany; 9 Department of Physiology & Biophysics, Stony Brook University, Stony Brook, NY USA; 10 Department of Biomedical Engineering, University of California, Irvine, Irvine, CA USA; 11 Molecular Microscopy Research Group, Zernike Institute for Advanced Materials, University of Groningen, Groningen, the Netherlands; 12 Physical and Synthetic Biology, Faculty of Biology, Ludwig-Maximilians-Universität München, Planegg-Martinsried, Germany; 13 Institute for Biophysics, Ulm University, Ulm, Germany; 14 Laboratory of Biophysics, Wageningen University & Research, Wageningen, the Netherlands; 15 Department of Biomedical Engineering, John Hopkins University, Baltimore, MD USA; 16 Department of Physics, North Carolina State University, Raleigh, NC USA; 17 B CUBE—Center for Molecular Bioengineering, TU Dresden, Dresden, Germany; 18 Laboratory for Photochemistry and Spectroscopy, Department of Chemistry, University of Leuven, Leuven, Belgium; 19 Dynamic Bioimaging Lab, Advanced Optical Microscopy Center and Biomedical Research Institute, Hasselt University, Hasselt, Belgium; 20 Institute of Physics, University of Lübeck, Lübeck, Germany; 21 Microspectroscopy Research Facility Wageningen, Wageningen University & Research, Wageningen, the Netherlands; 22 Gene Machines Group, Clarendon Laboratory, Department of Physics, University of Oxford, Oxford, UK; 23 School of Chemistry, Seoul National University, Seoul, South Korea; 24 Molecular Biophysics, Technische Universität Kaiserslautern (TUK), Kaiserslautern, Germany; 25 Departments of Biology and Chemistry, Pharmacy and Geosciences, Johannes Gutenberg-University Mainz, Mainz, Germany; 26 Institute of Molecular Biology (IMB), Mainz, Germany; 27 Structural and Computational Biology Unit, European Molecular Biology Laboratory (EMBL), Heidelberg, Germany; 28 School of Molecular Sciences and The Biodesign Institute, Arizona State University, Tempe, AZ USA; 29 Department of Biochemistry, University of Zurich, Zurich, Switzerland; 30 Department of Chemistry, Ludwig-Maximilians-Universität München, München, Germany; 31 Institute of Physical & Theoretical Chemistry, Braunschweig Integrated Centre of Systems Biology (BRICS), and Laboratory for Emerging Nanometrology (LENA), Braunschweig University of Technology, Braunschweig, Germany; 32 BIOSS Centre for Biological Signalling Studies, University of Freiburg, Freiburg im Breisgau, Germany

**Keywords:** DNA, Structure determination, Fluorescence resonance energy transfer, Single-molecule biophysics, Biophysical methods

## Abstract

Single-molecule Förster resonance energy transfer (smFRET) is increasingly being used to determine distances, structures, and dynamics of biomolecules in vitro and in vivo. However, generalized protocols and FRET standards to ensure the reproducibility and accuracy of measurements of FRET efficiencies are currently lacking. Here we report the results of a comparative blind study in which 20 labs determined the FRET efficiencies (*E*) of several dye-labeled DNA duplexes. Using a unified, straightforward method, we obtained FRET efficiencies with s.d. between ±0.02 and ±0.05. We suggest experimental and computational procedures for converting FRET efficiencies into accurate distances, and discuss potential uncertainties in the experiment and the modeling. Our quantitative assessment of the reproducibility of intensity-based smFRET measurements and a unified correction procedure represents an important step toward the validation of distance networks, with the ultimate aim of achieving reliable structural models of biomolecular systems by smFRET-based hybrid methods.

## Abstract (web summary)

A multi-laboratory study finds that single-molecule FRET is a reproducible and reliable approach for determining accurate distances in dye-labeled DNA duplexes.

## Main

FRET^1^, also known as fluorescence resonance energy transfer, is a well-established method for studying biomolecular conformations and dynamics at both the ensemble^2–4^ and the single-molecule level^5–10^. In such experiments, the energy transfer between donor and acceptor fluorophores is quantified with respect to their proximity^1^. The fluorophores are usually attached via flexible linkers to defined positions of the system under investigation. The transfer efficiency depends on the interdye distance, which is well described by Förster’s theory for distances > 30 Å^11,12^. Accordingly, FRET has been termed a ‘spectroscopic ruler’ for measurements on the molecular scale^2^, capable of determining distances in vitro, and even in cells^13^, with potentially ångström-level accuracy and precision. In its single-molecule implementation, FRET largely overcomes ensemble-averaging and time-averaging and can uncover individual species in heterogeneous and dynamic biomolecular complexes, as well as transient intermediates^5^.

The two most popular smFRET approaches for use in determining distances are confocal microscopy of freely diffusing molecules in solution and total internal reflection fluorescence (TIRF) microscopy of surface-attached molecules. Various fluorescence-intensity- and lifetime-based procedures have been proposed with the aim of determining FRET efficiencies^10,14–20^. Here we focus on intensity-based measurements in which the FRET efficiency *E* is determined from donor and acceptor photon counts and subsequently used to calculate the interfluorophore distance according to Förster’s theory.

So far most intensity-based smFRET studies have characterized relative changes in FRET efficiency. This ratiometric approach is often sufficient to distinguish different conformations of a biomolecule (e.g., an open conformation with low FRET efficiency versus a closed conformation with high FRET efficiency) and to determine their interconversion kinetics. However, knowledge about distances provides additional information that can be used, for example, to compare an experimental structure with known structures, or to assign conformations to different structural states. In combination with other structural measurements and computer simulations, FRET-derived distances are increasingly being used to generate novel biomolecular structural models via hybrid methods^7–9,21–26^.

However, it is difficult to compare and validate distance measurements from different labs, especially when detailed methodological descriptions are lacking. In addition, different methods for data acquisition and analysis, which often involve custom-built microscopes and in-house software, can have very different uncertainties and specific pitfalls. To overcome these issues, here we describe general methodological recommendations and well-characterized standard samples for FRET that can enable researchers to validate results and estimate the accuracy and precision of distance measurements. This approach should allow the scientific community to confirm the consistency of smFRET-derived distances and structural models. To facilitate data validation across the field, we recommend the use of a unified nomenclature to report specific FRET-related parameters.

The presented step-by-step procedure for obtaining FRET efficiencies and relevant correction parameters was tested in a worldwide, comparative, blind study by 20 participating labs. We show that, for standardized double-stranded DNA FRET samples, FRET efficiencies can be determined with an s.d. value of less than ±0.05.

To convert the measured smFRET efficiencies to distances, we used the Förster equation (equation (3); all numbered equations cited in this paper can be found in the Methods section), which critically depends on the dye-pair-specific Förster radius, *R*_0_. We discuss the measurements required to determine *R*_0_ and the associated uncertainties. Additional uncertainty arises from the fact that many positions are sampled by the dye relative to the biomolecule to which it is attached. Therefore, specific models are used to describe the dynamic movement of the dye molecule during the recording of each FRET-efficiency measurement^22,23^. The investigation of the uncertainties in FRET-efficiency determination and the conversion into distance measurements enabled us to specify uncertainties for individual FRET-derived distances.

## Results

### Benchmark samples and approaches

**Fig. 1:**
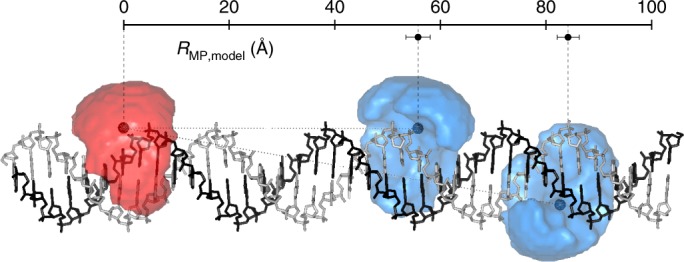
Schematic of the FRET standard molecules. Double-stranded DNA was labeled with a FRET pair at 15-bp or 23-bp separation for the “lo” and “mid” samples, respectively (sequences are provided in the Methods). The accessible volumes (AVs) of the dyes (donor, blue; acceptor, red) are illustrated as semi-transparent surfaces and were calculated with freely available software^8^. The mean dye positions are indicated by darker spheres (assuming homogeneously distributed dye positions; Supplementary Note 3). The distance between the mean dye positions is defined as *R*_MP,model_. Calculated values for *R*_MP,model_ and the errors obtained by varying parameters of the AV model are shown (Supplementary Note 3). The B-DNA model was generated with Nucleic Acid Builder version 04/17/2017 for Amber^27^.

We chose double-stranded DNA as a FRET standard for several reasons: DNA sequences can be synthesized, FRET dyes can be specifically tethered at desired positions, the structure of B-form DNA is well characterized, and the samples are stable at room temperature long enough that they can be shipped to labs around the world. The donor and acceptor dyes were attached via C2 or C6 amino linkers to thymidines of opposite strands (Supplementary Fig. 1). These thymidines were separated by 23 bp, 15 bp (Fig. 1), or 11 bp (Supplementary Fig. 1, Supplementary Table 1, and Supplementary Note 1). The attachment positions were known only to the reference lab that designed the samples. The samples were designed in such a way that we were able to determine all correction parameters and carry out a self-consistency test (described below).

In this study we used Alexa Fluor and Atto dyes because of their high quantum yields and well-studied characteristics (Supplementary Note 2). Eight hybridized double-stranded FRET samples were shipped to all participating labs. In the main text, we focus on four FRET samples that were measured by most labs in our study:

- 1-lo: Atto 550/Atto 647N; 23-bp separation
- 1-mid: Atto 550/Atto 647N; 15-bp separation
- 2-lo: Atto 550/Alexa Fluor 647; 23-bp separation
- 2-mid: Atto 550/Alexa Fluor 647; 15-bp separation

In revision, 13 labs evaluated two additional samples:

- 1-hi: Atto 550/Atto 647N; 11-bp separation
- 2-hi: Atto 550/Alexa Fluor 647; 11-bp separation

In this nomenclature, the number refers to the dye pair, and lo, mid, and hi indicate low-efficiency, medium-efficiency, and high-efficiency configurations, respectively. The results with other FRET pairs (Alexa Fluor 488/Alexa Fluor 594 and Alexa Fluor 488/Atto 647N) at these positions, per lab, for all samples and for different methods, are reported in Supplementary Fig. 2 and Supplementary Note 2.

To avoid dye stacking^28,29^, we designed the DNA molecules such that the dyes were attached to internal positions sufficiently far from the duplex ends. As a first test for the suitability of the labels, we checked the fluorescence lifetimes and time-resolved anisotropies (Supplementary Table 2) of all donor-only and acceptor-only samples. The results indicated that there was no significant quenching or stacking and that all dyes were sufficiently mobile at these positions (Supplementary Note 2).

Most measurements were carried out on custom-built setups that featured at least two separate spectral detection channels for donor and acceptor emission (Supplementary Figs. 3 and 4). Results obtained with different fluorophores (samples 3 and 4) and different FRET methods (ensemble lifetime^30^, single-molecule lifetime^16^, and a phasor approach^31^) are presented in Supplementary Fig. 2 and Supplementary Notes 1 and 2.

A robust correction procedure to determine absolute fluorescence intensities is needed. The ideal solution is a ratiometric approach that, for intensity-based confocal FRET measurements, was pioneered by Weiss and coworkers and uses alternating two-color laser excitation (ALEX) with microsecond pulses^17,32^. In this approach the fluorescence signal after donor excitation is divided by the total fluorescence signal after donor and acceptor excitation (referred to as apparent stoichiometry; see equation (16)), to correct for dye and instrument properties^17^. The ALEX approach was also adapted for TIRF measurements^20^. To increase time resolution and to enable time-resolved spectroscopy, Lamb and coworkers introduced pulsed interleaved excitation with picosecond pulses^33^.

### Procedure to determine the experimental FRET efficiency 〈*E*〉

In both confocal and TIRF microscopy, the expectation value of the FRET efficiency 〈*E*〉 is computed from the corrected FRET efficiency histogram. In this section, first we outline a concise and robust procedure to obtain 〈*E*〉. Then we describe distance and uncertainty calculations, assuming a suitable model for the interdye distance distribution and dynamics^6,11,34^. Finally, we derive self-consistency arguments and comparisons to structural models to confirm the accuracy of this approach.

Our general procedure is largely based on a previous approach^17^, with modifications to establish a robust workflow and standardize the nomenclature. Intensity-based determination of FRET efficiencies requires consideration of the following correction factors (details in the Methods section): background signal correction (BG) from donor and acceptor channels; *α*, a factor for spectral cross-talk arising from donor fluorescence leakage in the acceptor channel; *δ*, a factor for direct excitation of the acceptor with the donor laser; and a detection correction factor (*γ*). The optimal way to determine these factors is to alternate the excitation between two colors, which allows for determination of the FRET efficiency (*E*) and the relative stoichiometry (*S*) of donor and acceptor dyes, for each single-molecule event. This requires the additional excitation correction factor *β* to normalize the excitation rates.

The following step-by-step guide presents separate instructions for confocal and TIRF experiments; notably, the order of the steps is crucial (Methods).

#### Diffusing molecules: confocal microscopy

**Fig. 2:**
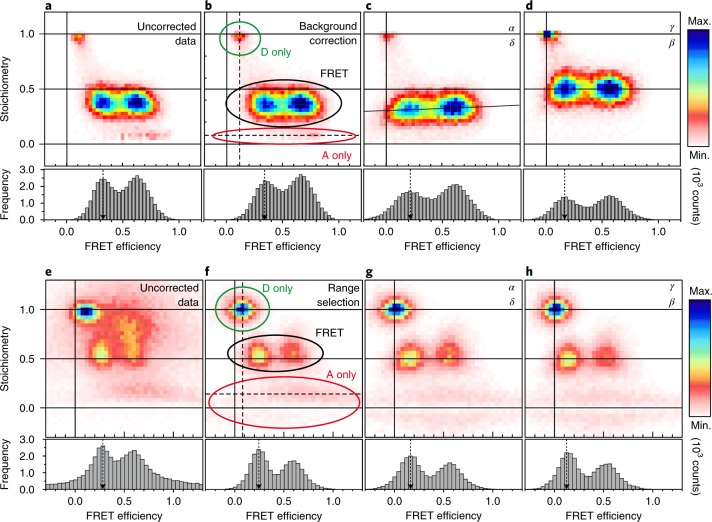
Stepwise data correction for 1-lo and 1-mid samples. **a**–**d**, Workflow for correction of the confocal data for background (**a** → **b**); leakage (factor *α*); and direct excitation (*δ*) (**b** → **c**), excitation, and detection factors (*β*, *γ*) (**c** → **d**). **e**–**h**, Workflow for correction of TIRF data for background and photobleaching by selection of the prebleached range (**e** → **f**); leakage; and direct excitation (**f** → **g**), detection, and excitation factors (**g** → **h**). The efficiency histograms show a projection of the data with a stoichiometry between 0.3 and 0.7. The general terms “stoichiometry” and “FRET efficiency” are used in place of the corresponding specific terms for each correction step. Donor (D)-only, FRET, and acceptor (A)-only populations are specified.

Photon arrival times from individual molecules freely diffusing through the laser focus of a confocal microscope are registered. Signal threshold criteria are applied, and bursts are collected and analyzed. From the data, first a 2D histogram of the uncorrected FRET efficiency (^i^*E*_app_) versus the uncorrected stoichiometry (^i^*S*_app_) is generated (Fig. 2a). Then the average number of background photons is subtracted for each channel separately (Fig. 2b). Next, to obtain the FRET sensitized acceptor signal (*F*_A|D_), one must subtract the donor leakage (*α*^ii^*I*_Dem|Dex_) and direct excitation (*δ*^ii^*I*_Aem|Aex_) from the acceptor signal after donor excitation. As samples never comprise 100% photoactive donor and acceptor dyes, the donor-only and acceptor-only populations are selected from the measurement and used to determine the leakage and direct excitation (Fig. 2c). After this correction step, the donor-only population should have an average FRET efficiency of 0, and the acceptor-only population should have an average stoichiometry of 0.

The last step deals with the detection correction factor *γ* and the excitation correction factor *β*. If at least two species (two different samples or two populations within a sample) with different interdye distances are present, they can be used to obtain the ‘global *γ*-correction’. If one species with substantial distance fluctuations (e.g., from intrinsic conformational changes) is present, a ‘single-species *γ*-correction’ may be possible. Both correction schemes assume that the fluorescence quantum yields and extinction coefficients of the dyes are independent of the attachment point. The correction factors obtained by the reference lab are compiled in Supplementary Table 3. The final corrected FRET efficiency histograms are shown in Fig. 2d. The expected efficiencies 〈*E*〉 are obtained as the mean of a Gaussian fit to the respective efficiency distributions. After correction, we noted a substantial shift of the FRET-efficiency peak positions, especially for the low-FRET-efficiency peak (*E* ~ 0.25 uncorrected to *E* ~ 0.15 when fully corrected).

#### Surface-attached molecules: TIRF microscopy

The correction procedure for TIRF-based smFRET experiments is similar to the procedure for confocal-based experiments. In the procedure used for ALEX data^20^, a 2D histogram of the uncorrected FRET efficiency versus the uncorrected stoichiometry is generated (Fig. 2e). The background subtraction is critical in TIRF microscopy, as it can contribute substantially to the measured signal. Different approaches can be used to accurately determine the background signal, such as measuring the background in the vicinity of the selected particle or measuring the intensity after photobleaching (Fig. 2f). After background correction, the leakage and direct excitation can be calculated from the ALEX data as for confocal microscopy (Fig. 2g).

Again, determination of the correction factors *β* and *γ* is critical^15^. As with confocal microscopy, one can use the stoichiometry information available from ALEX when multiple populations are present to determine an average detection correction factor (global *γ*-correction). In TIRF microscopy, the detection correction factor can also be determined on a molecule-by-molecule basis, provided the acceptor photobleaches before the donor (individual *γ*-correction). In this case, the increase in the fluorescence of the donor can be directly compared to the intensity of the acceptor before photobleaching. A 2D histogram of corrected FRET efficiency versus corrected stoichiometry is shown in Fig. 2h.

In the absence of alternating laser excitation, the following problems occasionally arose during this study: (i) the low-FRET-efficiency values were shifted systematically to higher efficiencies, because FRET-efficiency values at the lower edge were overlooked owing to noise; (ii) the direct excitation was difficult to detect and correct because of its small signal-to-noise ratio; and (iii) acceptor bleaching was difficult to detect for low FRET efficiencies. Therefore, we strongly recommend implementing ALEX in order to obtain accurate FRET data.

**Fig. 3:**
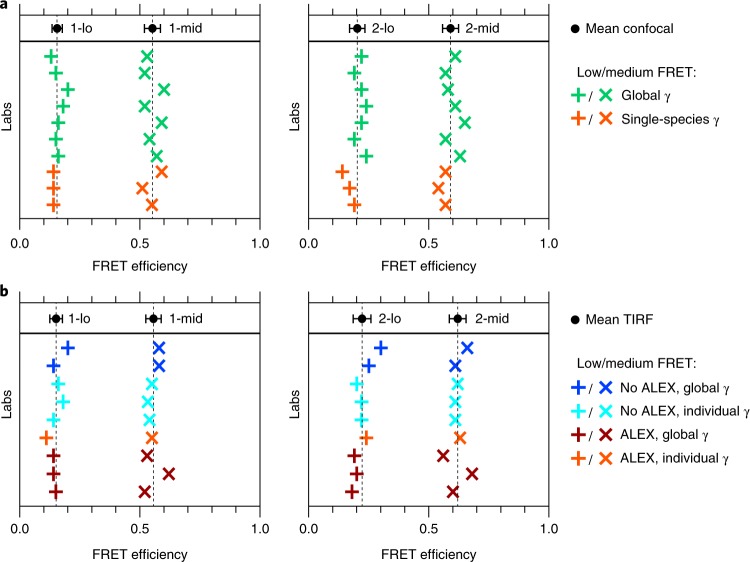
Summary of the results of the intensity-based methods. **a**, Confocal measurements. **b**, TIRF measurements. Note that some laboratories performed measurements with both methods. The mean ± s.d. is depicted in the upper portion of each plot. Dashed lines indicate mean values (summarized in Supplementary Table 4). Example correction factors are given in Supplementary Table 3.

Nine of the twenty participating labs determined FRET efficiencies by confocal methods for samples 1 and 2 (Fig. 3a). Seven of the twenty participating labs determined FRET efficiencies by TIRF-based methods (Fig. 3b). The combined data from all labs for measurements of samples 1 and 2 agree very well, with s.d. for the complete dataset of Δ*E* < ±0.05. This is a remarkable result, considering that different setup types were used (confocal- and TIRF-based setups) and different correction procedures were applied (e.g., individual, global, or single-species *γ*-correction).

### Distance determination

The ultimate goal of this approach is to derive distances from FRET efficiencies. The efficiency-to-distance conversion requires knowledge of the Förster radius, *R*_0_, for the specific FRET pair used and of a specific dye model describing the behavior of the dye attached to the macromolecule^22,23^. In the following, we describe (i) how *R*_0_ can be determined and (ii) how to use a specific dye model to calculate two additional values, *R*_〈*E*〉_ and *R*_MP_. *R*_〈*E*〉_ is the apparent distance between the donor and the acceptor, which is directly related to the experimental FRET efficiency 〈*E*〉 that is averaged over all sampled donor–acceptor distances *R*_DA_ (equation (5)), but it is not a physical distance. *R*_MP_ is the real distance between the center points (mean positions) of the accessible volumes and deviates from *R*_〈*E*〉_ because of the different averaging in distance and efficiency space. *R*_MP_ cannot be measured directly but is important, for example, for mapping the physical distances required for structural modeling^34^.

*R*_0_ is a function of equation (7) and depends on the index of refraction of the medium between the two fluorophores (*n*_im_), the spectral overlap integral (*J*), the fluorescence quantum yield of the donor (Φ_F,D_), and the relative dipole orientation factor (*κ*^2^) (an estimate of their uncertainties is provided in the Methods section). Our model assumes that the FRET rate (*k*_FRET_) is much slower than the rotational relaxation rate (*k*_rot_) of the dye and that the translational diffusion rate (*k*_diff_) allows the dye to sample the entire accessible volume within the experimental integration time (1/*k*_int_), that is, *k*_rot_ >> *k*_FRET_ >> *k*_diff_ >> *k*_int_. The validity of these assumptions is justified by experimental observables discussed in the Methods.

The determined Förster radii for samples 1 and 2 are given in Supplementary Table 4. Note that literature values differ mainly because donor fluorescence quantum yields are not specified and the refractive index of water is often assumed, whereas we used *n*_im_ = 1.40 here. Our careful error analysis led to an error estimate of 7% for the determined *R*_0_, which is relatively large (mainly owing to the uncertainty in *κ*^2^).

**Fig. 4:**
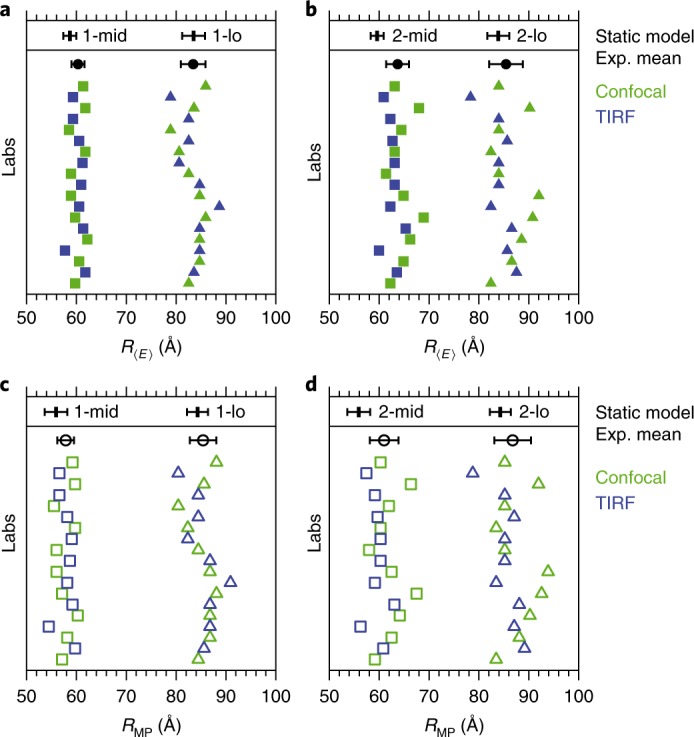
Mean interdye distances determined from 19 〈*E*〉 values measured in 16 different labs. **a**,**b**, *R*_〈*E*〉_ for samples 1 (**a**) and 2 (**b**). **c**,**d**, *R*_MP_ for samples 1 (**c**) and 2 (**d**). Data are shown as individual values (colored symbols) and as the mean (black dots) and s.d., assuming *R*_0_ = 62.6 Å and *R*_0_ = 68.0 Å for samples 1 and 2, respectively. The black bars at the top of each plot indicate the static model values and their error (determined by variation of model parameters); see Supplementary Table 4 for values. The depicted errors include only the statistical variations of the FRET efficiencies, and do not include the error in the Förster radii; thus these errors represent the precision of the measurement, but not the accuracy. Exp., experimental.

We used the measured smFRET efficiencies and the calculated Förster radii to compute the apparent distance *R*_〈*E*〉_ from each lab’s data (equation (5)). Figure 4a,b shows the calculated values for these apparent distances for samples 1 and 2 for each data point in Fig. 3. The average values for all labs are given in Supplementary Table 4, together with model values based on knowledge of the dye attachment positions, the static DNA structure, and the mobile dye model (Supplementary Note 3). Considering the error ranges, the experimental and model values agree very well with each other (the deviations range between 0 and 8%).

Although this study focused on measurements on DNA, the described FRET analysis and error estimation are fully generalizable to other systems (e.g., proteins), assuming mobile dyes are used. What becomes more difficult with proteins is specific dye labeling, and the determination of an appropriate dye model, if the dyes are not sufficiently mobile (Supplementary Note 3). *R*_〈*E*〉_ corresponds to the real distance *R*_MP_ only in the hypothetical case in which both dyes are unpolarized point sources, with zero accessible volume (AV). In all other cases, *R*_MP_ is the only physical distance. It can be calculated in two ways: (i) if the dye model and the local environment of the dye are known, simulation tools such as the FRET Positioning and Screening tool^8^ can be used to compute *R*_MP_ from *R*_〈*E*〉_ for a given pair of AVs; or (ii) if the structure of the investigated molecule is unknown a priori, a sphere is a useful assumption for the AV. In both cases, a lookup table is used to convert *R*_〈*E*〉_ to *R*_MP_ for defined AVs and *R*_0_ values (Supplementary Note 5). Our results for these calculations, given as distances determined via the former approach, are shown in Fig. 4c,d and Supplementary Table 4. The respective model values are based on the center points of the AVs depicted in Fig. 1 and given in Supplementary Table 4 (details in Supplementary Note 3).

### Distance uncertainties

**Fig. 5:**
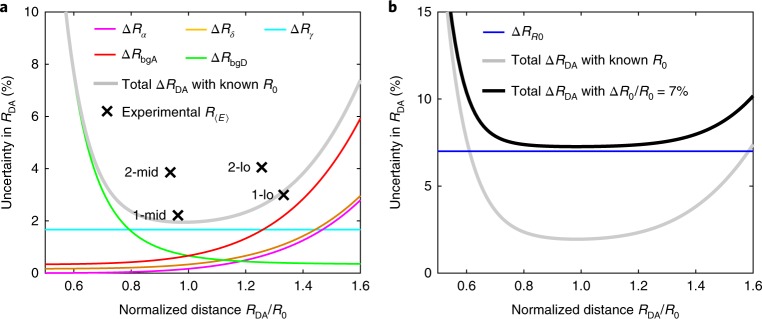
Error propagation of experimental uncertainty. **a**, *R*_DA_ uncertainty contributions from the experimental correction factors: ∆*R*_*γ*_ (gamma factor), Δ*R*_bgD_ and Δ*R*_bgA_ (background), ∆*R*_*α*_ (leakage), ∆*R*_*δ*_ (direct excitation), and total uncertainty with known *R*_0_; crosses indicate the uncertainty of experimental values of *R*_〈*E*〉_ across the labs. **b**, Uncertainty in *R*_DA_ (black line) based on the efficiency-related uncertainty (gray line) and the uncertainty for determining *R*_0_ (blue line). Here we used the following uncertainties, which were determined for the confocal-based measurements on sample 1: Δ*R*_0_/*R*_0_ = 7%, Δ*γ*/*γ* = 10%, Δ*I*^(BG)^/*I* = 2%, Δ*α*/*α* = 10%, and Δ*δ*/*δ* = 10%. Absolute values are presented in Supplementary Table 3.

We estimated all uncertainty sources arising from both the measurement of the corrected FRET efficiencies and the determination of the Förster radius, and propagated them into distance uncertainties. We discuss the error in determining the distance between two freely rotating but spatially fixed dipoles, *R*_DA_, with the Förster equation (equation (26)). Figure 5a shows how uncertainty in each of the correction factors (*α*, *γ*, and *δ*) and the background signals (BG_D_, BG_A_) is translated into the uncertainty of *R*_DA_ (Supplementary Note 6). The uncertainty of *R*_MP_ is similar but depends on the dye model and the AVs. The solid gray line in Fig. 5a  shows the sum of these efficiency-dependent uncertainties, which are mainly setup-specific quantities. For the extremes of the distances, the largest contribution to the uncertainty in *R*_DA_ arises from background photons in the donor and acceptor channels. In the presented example with *R*_0_ *=* 62.6 Å, the total uncertainty Δ*R*_DA_ based on the setup-specific uncertainties is less than 4 Å for 35 Å < *R*_DA_ < 90 Å. Notably, in confocal measurements, larger intensity thresholds can decrease this uncertainty further. The uncertainty in *R*_DA_ arising from errors in *R*_0_ (blue line in Fig. 5b) is added to the efficiency-related uncertainty in *R*_DA_ (bold gray line in Fig. 5b) to estimate the total experimental uncertainty in *R*_DA_ (black line in Fig. 5b). The uncertainties for determining *R*_0_ are dominated by the dipole orientation factor *κ*^2^ and the refractive index *n*_im_ (Methods). Including the uncertainty in *R*_0_, the error Δ*R*_DA,total_ for a single smFRET-based distance between two freely rotating point dipoles is less than 6 Å for 35 Å < *R*_DA_ < 80 Å. The uncertainty is considerably reduced when multiple distances are calculated and self-consistency in distance networks is exploited^9^. Besides background contributions, an *R*_DA_ shorter than 30 Å may lead to larger errors due to (i) potential dye–dye interactions and (ii) the dynamic averaging of the dipole orientations being reduced owing to an increased FRET rate.

### Comparing distinct dye pairs

To validate the model assumption of a freely rotating and diffusing dye, we developed a self-consistency argument based on the relationship between different dye pairs that bypasses several experimental uncertainties. We define the ratio *R*_rel_ for two dye pairs as the ratio of their respective *R*_〈*E*〉_ values (Methods, equation (30)). This ratio is quasi-independent of *R*_0_, because all dye parameters that are contained in *R*_0_ are approximately eliminated by our DNA design. Therefore, these ratios should be similar for all investigated dye pairs, which we indeed found was the case (Supplementary Table 4). When comparing, for example, the low- to mid-distances for three dye pairs with *E* > 0.1, we obtained a mean *R*_rel_ of 1.34 and a maximum deviation of 2.7%. This is a relative error of 2.3%, which is less than the estimated error of our measured distances of 2.8% (Fig. 5a). This further demonstrates the validity of the assumptions for the dye model and averaging regime used here. This concept is discussed further in the  Methods.

Although calculated model distances are based on a static model for the DNA structure, DNA is known not to be completely rigid^35^. We tested our DNA model by carrying out molecular dynamics simulations using the DNA molecule (without attached dye molecules; Supplementary Note 7) and found that the averaged expected FRET efficiency obtained with the computed dynamically varying slightly bent DNA structure led to comparable but slightly longer distances than for the static model. The deviations between the models and data were reduced (Supplementary Table 4) for those cases where we observed larger deviations with static models.

## Discussion

Despite differences in the setups used, the reported intensity-based FRET efficiencies were consistent between labs in this study. We attribute this remarkable consistency (Δ*E* < ±0.05) to the use of a general step-by-step procedure for the experiments and data analysis.

We also showed that the factors required for the correction of FRET efficiency can be determined with high precision, regardless of the setup type and acquisition software used. Together the measurement errors caused an uncertainty in *R*_DA_ of less than 5%, which agrees well with the variations between the different labs. Ultimately, we were interested in the absolute distances derived from these FRET efficiencies. Figure 5 shows that any distance between 0.6 *R*_0_ and 1.6 *R*_0_ could be determined with an uncertainty of less than ±6 Å. This fits well with the distance uncertainty measured across the labs and corresponds to a distance range from 35 to 80 Å for the dye pairs used in sample 1. This estimation is valid if the dyes are sufficiently mobile, as has been supported by time-resolved anisotropy measurements and further confirmed by a self-consistency argument. The s.d. for sample 2 was slightly larger than that for sample 1 (Fig. 5a), which could be explained by specific photophysical properties. The values for samples 3 and 4 (Supplementary Table 4) showed similar precision, considering the smaller number of measurements.

For the samples 1-hi and 2-hi, which were measured after each lab verified its setup and procedure, the precision was further increased by almost a factor of two (Supplementary Table 4), possibly owing to the thorough characterization during this study.

We also tested the accuracy of the experimentally derived distances by comparing them with distances in the static model. For every single FRET pair we found excellent agreement between 0.1% and 4.1% (0.4–2.4 Å) for sample 1 and agreement mostly within the range of experimental error between 3.1% and 9.0% (2.7–5.5 Å) for sample 2. The deviations could be even smaller for dynamic DNA models. For sample 2, which had the cyanine-based dye Alexa Fluor 647 instead of the carbopyronine-based dye Atto 647N as an acceptor, the lower accuracy could be explained by imperfect sampling of the full AV or dye-specific photophysical properties (details are presented in Supplementary Table 2). It was shown previously that cyanine dyes are sensitive to their local environment^36^ and therefore require especially careful characterization for each newly labeled biomolecule.

For future work, it will be powerful to complement intensity-based smFRET studies with single-molecule lifetime studies, as the picosecond time resolution could provide additional information on calibration and fast dynamic biomolecular exchange. In addition, it will be important to establish appropriate dye models for more complex (protein) systems in which the local chemistry may affect dye mobility (Supplementary Note 4). However, when used with mobiles dyes (which can be checked via anisotropy and lifetime experiments; Supplementary Note 2), the dye model here is fully generalizable to any biomolecular system^8,9^.

The results from different labs and the successful self-consistency test clearly show the great potential of absolute smFRET-based distances for investigations of biomolecular conformations and dynamics, as well as for integrative structural modeling. The ability to accurately determine distances on the molecular scale with smFRET experiments and to estimate the uncertainty of the measurements provides the groundwork for smFRET-based structural and hybrid approaches. Together with the automated selection of the most informative pairwise labeling positions^23^ and fast analysis procedures^8–10^, we anticipate that smFRET-based structural methods will become an important tool for de novo structural determination and structure validation, especially for large and flexible structures with which the application of other structural biology methods is difficult.

## Methods

### Nomenclature and definitions

See Supplementary Table 5 for a summary of the following section.

The FRET efficiency *E* is defined as

1 $$E = \frac{{F_{\rm{A|D}}}}{{F_{\rm{D|D}} + F_{\rm{A|D}}}}$$

where *F* is the signal. The stoichiometry *S* is defined as

2 $$S = \frac{{F_{\rm{D|D}} + F_{\rm{A|D}}}}{{F_{\rm{D|D}} + F_{\rm{A|D}} + F_{\rm{A|A}}}}$$

The FRET efficiency for a single donor–acceptor distance *R*_DA_ is defined as

3 $$E = \frac{1}{{1 + R_{\rm{DA}}^6/R_0^6}}$$

The mean FRET efficiency for a discrete distribution of donor–acceptor distances with the position vectors ${\boldsymbol{R}}_{{\rm{D}}(i)}$ and ${\boldsymbol{R}}_{{\rm{A}}(j)}$ is calculated as

4 $$\left\langle E \right\rangle = \frac{1}{{nm}}\mathop {\sum }\limits_{{\boldsymbol{i}} = 1}^{\boldsymbol{n}} \mathop {\sum }\limits_{{\boldsymbol{j}} = 1}^{\boldsymbol{m}} \,\frac{1}{{1 + \left| {{\boldsymbol{R}}_{{\rm{A}}(j)} - {\boldsymbol{R}}_{{\rm{D}}(i)}} \right|^6/R_0^6}}$$

The apparent donor–acceptor distance *R*_〈*E*〉_ is computed from the average FRET efficiency for a distance distribution. It is a FRET-averaged quantity that is also referred to as the FRET-averaged distance 〈*R*_DA_〉_E_ (ref. ^37^):

5 $$R_{\langle E\rangle} \equiv R\left( E \right) = R_0\left( {E^{ - 1} - 1} \right)^{1/6}$$

The distance between the mean dye positions with the position vectors ${\boldsymbol{R}}_{{\rm{D}}\left( i \right)}$ and ${\boldsymbol{R}}_{{\rm{A}}\left( j \right)}$ is obtained by normalization of sums over all positions within the respective AVs:

6 $$R_{{\rm{MP}}} = \left| {\left\langle {{\boldsymbol{R}}_{{\rm{D}}(i)}} \right\rangle - \left\langle {{\boldsymbol{R}}_{{\rm{A}}\left( j \right)}} \right\rangle } \right| = \left| {\frac{1}{n}\mathop {\sum }\limits_{i = 1}^n {\boldsymbol{R}}_{{\rm{D}}(i)} - \frac{1}{m}\mathop {\sum }\limits_{j = 1}^m {\boldsymbol{R}}_{{\rm{A}}(j)}} \right|$$

Definitions of abbreviations in subscripts and superscripts are as follows:

- D or A: donor or acceptor
- A|D: acceptor fluorescence upon donor excitation (similarly for D|D, A|A, etc.)
- Aem|Dex: intensity in the acceptor channel upon donor excitation (similarly for Dem|Dex, Aem|Aex, etc.)
- app: apparent, that is, including systematic, experimental offsets
- BG: background
- DO/AO: donor-only/acceptor-only species
- DA: FRET species
- i–iii: (i) the uncorrected intensity; (ii) intensity after BG correction; (iii) intensity after BG, *α*, and *δ* corrections

The four correction factors are defined as follows.

Leakage of donor fluorescence into the acceptor channel:

$$\alpha = \frac{{g_{\rm{R|D}}}}{{g_{\rm{G|D}}}} = \frac{{\left\langle {\,^{\rm{ii}}{{E}}_{{\rm{app}}}^{\left( {{\rm{DO}}} \right)}} \right\rangle }}{{1 - \left\langle {\,^{\rm{ii}}{{E}}_{{\rm{app}}}^{\left( {{\rm{DO}}} \right)}} \right\rangle }}$$

Normalization of excitation intensities *I* and cross-sections *σ* of the acceptor and donor:

$${\mathrm{\beta }} = \frac{{\sigma _{\rm{A|R}}}}{{\sigma _{\rm{D|G}}}}\frac{{I_{\rm{Aex}}}}{{I_{\rm{Dex}}}}$$

Normalization of effective fluorescence quantum yields, ^**eff**^Φ_*F*_ = *a*_b_Φ_*F*_, and detection efficiencies *g* of the acceptor and donor, where *a*_b_ is the fraction of molecules in the bright state and Φ_*F*_ is the fluorescence quantum yield without photophysical (saturation) effects:

$$\gamma = \frac{{g_{\rm{R|A}}}}{{g_{\rm{G|D}}}}\frac{{\,{}^{\rm{eff}}{\mathrm{\Phi }}_{{F,\rm{A}}}}}{{\,{}^{\rm{eff}}{\mathrm{\Phi }}_{{F,\rm{D}}}}}$$

Direct acceptor excitation by the donor excitation laser (lower wavelength):

$${\mathrm{\delta }} = \frac{{\sigma _{\rm{A|G}}}}{{\sigma _{\rm{A|R}}}}\frac{{I_{\rm{Dex}}}}{{I_{\rm{Aex}}}} = \frac{{\left\langle {\,{}^{\rm{ii}}S_{\rm{app}}^{({\rm{AO}})}} \right\rangle }}{{1 - \left\langle {\,{}^{\rm{ii}}S_{\rm{app}}^{({\rm{AO}})}} \right\rangle }}$$

where *I* is the experimentally observed intensity; *F* indicates the corrected fluorescence intensity; ${\mathrm{\Phi }}_{F,\rm{A}}\,{\mathrm{and}}\,{\mathrm{\Phi }}_{F,\rm{D}}$ are the fluorescence quantum yield of the acceptor and the donor, respectively; *g*_R|A_ and *g*_G|D_ represent the detection efficiency of the red detector (R) if only the acceptor was excited or green detector (G) if the donor was excited (analogously for other combinations); and *σ*_Α_|_G_ is the excitation cross-section for the acceptor when excited with green laser (analogously for the other combinations).

The Förster radius (in angstroms) for a given *J* in the units shown below is given by

7 $$\frac{{R_0}}{{\rm{\AA}}} = 0.2108\,\root {6} \of {{\left( {\frac{{{\mathrm{\Phi }}_{{F},\mathrm{D}}\kappa ^2}}{{n_{{\rm{im}}}^4}}} \right)\frac{J}{{\mathrm{M}^{-1}\mathrm{cm}^{-1}\mathrm{nm}^4}}}}$$

with the dipole orientation factor $\kappa ^2 = \left( {\cos \theta _{\rm{AD}} - 3\cos \theta _{\rm{D}}\cos \theta _{\rm{A}}} \right)^2$ and the spectral overlap integral (in cm^–1^ M^–1^ nm^4^)

$$J = \mathop {\smallint }\limits_0^\infty {\bar{F}}_{\rm{D}}\left( \lambda \right)\varepsilon _{\rm{A}}\left( \lambda \right)\lambda ^4{\mathrm{d}}\lambda$$

with the normalized spectral radiant intensity of the excited donor (in nm^–1^), defined as the emission intensity *F* per unit wavelength,

$${\bar{F}}_{\rm{D}}\left( \lambda \right)\:{\mathrm{with}}\,\mathop {\smallint }\limits_0^\infty \bar F_{\rm{D}}\left( \lambda \right){\mathrm{d}}\lambda = 1$$

and the extinction coefficient of the acceptor (in M^–1^ cm^–1^), $\varepsilon _{\rm{A}}(\lambda )$, and the refractive index of the medium between the dyes, *n*_im_.

### Samples

Altogether, eight different FRET samples were designed with the acceptor dyes positioned 15 or 23 bp away from the donor dyes. The exact sequences and dye positions are given in Supplementary Table 1 and Supplementary Note 1. We ordered them from IBA GmbH (Göttingen), which synthesized and labeled the single DNA strands and then carried out HPLC purification. Here the dyes were attached to a thymidine (dT), which is known to cause the least fluorescence quenching of all nucleotides^26^.

Most labs measured the four DNA samples listed in Supplementary Table 1. Therefore, we focus on these four samples in the main text of this paper. The additional samples and the corresponding measurements are described in Supplementary Note 1, Supplementary Fig. 2, and Supplementary Table 4. A buffer consisting of 20 mM MgCl_2_, 5 mM NaCl, 5 mM Tris, pH 7.5, was requested for all measurements, with de-gassing just before the measurement at room temperature.

The linker lengths were chosen in such a way that all dyes had about the same number of flexible bonds between the dipole axis and the DNA. Atto 550, Alexa Fluor 647, and Atto 647N already have an intrinsic flexible part before the C-linker starts (Supplementary Fig. 1). In addition, the DNAs were designed such that the distance ratio between the high-FRET-efficiency and low-FRET-efficiency samples should be the same for all samples, largely independent of *R*_0_.

Details on all used setups and analysis software are presented in Supplementary Note 8.

### General correction procedure

The FRET efficiency *E* and stoichiometry *S* are defined in equations (1) and (3). Determination of the corrected FRET *E* and *S* is based largely on the approach of Lee et al.^17^ and consists of the following steps: (1) data acquisition, (2) generation of uncorrected 2D histograms for *E* versus *S*, (3) background subtraction, (4) correction for position-specific excitation in TIRF experiments, (5) correction for leakage and direct acceptor excitation, and (6) correction for excitation intensities and absorption cross-sections, quantum yields, and detection efficiencies.

#### Data acquisition

The sample with both dyes is measured, and three intensity time traces are extracted: acceptor emission upon donor excitation ($I_{\rm{Aem|Dex}}$), donor emission upon donor excitation ($I_{\rm{Dem|Dex}}$), and acceptor emission upon acceptor excitation ($I_{\rm{Aem|Aex}}$).

For the confocal setups, a straightforward burst identification is carried out in which the trace is separated into 1-ms bins. Usually a minimum threshold (e.g., 50 photons) is applied to the sum of the donor and acceptor signals after donor excitation for each bin. This threshold is used again in every step, such that the number of bursts used may change from step to step (if the *γ* correction factor is not equal to 1). Some labs use sophisticated burst-search algorithms. For example, the dual-channel burst search^38,39^ recognizes the potential bleaching of each dye within bursts. Note that the choice of burst-search algorithm can influence the *γ* correction factor. For standard applications, the simple binning method is often sufficient, especially for well-characterized dyes and low laser powers. This study shows that the results do not depend heavily on these conditions (if they are applied properly), as every lab used its own setup and procedure at this stage. The number of photon bursts per measurement was typically between 1,000 and 10,000.

For the TIRF setups, traces with one acceptor and one donor are selected, defined by a bleaching step. In addition, only the relevant range of each trajectory (i.e., prior to photobleaching of either dye) is included in all subsequent steps. The mean length of the time traces analyzed by the reference lab was 47 frames (18.8 s) for the 185 traces of sample 1-lo and 15 frames (6 s) for the 124 traces of sample 2-lo measured at an ALEX sampling rate of 2.5 Hz. For sample 1, bleaching was donor limited, whereas bleaching for sample 2 was acceptor limited, which explains the significant difference in frame lengths. For details on the analysis from the reference lab, see ref. ^40^.

#### 2D histogram

A 2D histogram (Fig. 2a,e) of the apparent (uncorrected) stoichiometry, ${}^{\rm{i}}S_{\rm{app}}$, versus the apparent FRET efficiency, ${}^{\rm{i}}E_{\rm{app}}$, defined by equations (8) and (9), is generated, where

8 $$\,{}^{\rm{i}}S_{\rm{app}} = \frac {{I_{\rm{Aem|Dex}} + I_{\rm{Dem|Dex}}}}{{I_{\rm{Aem|Dex}} + I_{\rm{Dem|Dex}} + I_{\rm{Aem|Aex}}}}$$

9 $$\,{}^{\rm{i}}E_{\rm{app}} = \frac {I_{\rm{Aem|Dex}}}{{I_{\rm{Aem|Dex}} + I_{\rm{Dem|Dex}}}}$$

#### Background correction

Background *I*^(BG)^ is removed from each uncorrected intensity ^i^*I* separately, thus leading to the background-corrected intensities $\,{}^{\rm{ii}}I,\,{}^{\rm{ii}}S_{\rm{app}},\,{\mathrm{and}}\,{}^{\rm{ii}}E_{\rm{app}}$:

10 $$\begin{array}{*{20}{c}} {\,{}^{\rm{ii}}I_{\rm{Dem|Dex}} = \,{}^{\rm{i}}I_{\rm{Dem|Dex}} - I_{\rm{Dem|Dex}}^{\rm{(BG)}}} \cr {\,{}^{\rm{ii}}I_{\rm{Aem|Aex}} = \,{}^{\rm{i}}I_{\rm{Aem|Aex}} - I_{\rm{Aem|Aex}}^{\rm{(BG)}}} \cr {\,{}^{\rm{ii}}I_{\rm{Aem|Dex}} = \,{}^{\rm{i}}I_{\rm{Aem|Dex}} - I_{\rm{Aem|Dex}}^{\rm{(BG)}}} \end{array}$$

For confocal measurements, one can determine the background by averaging the photon count rate for all time bins that are below a certain threshold, which is defined, for example, by the maximum in the frequency-versus-intensity plot (the density of bursts should not be too high). Note that a previous measurement of only the buffer can uncover potential fluorescent contaminants, but may differ substantially from the background of the actual measurement. The background intensity is then subtracted from the intensity of each burst in each channel (equation (10)). Typical background values are 0.5–1 photon/ms (Fig. 2b).

For TIRF measurements, various trace-wise or global background corrections can be applied. The most common method defines background as the individual offset (time average) after photobleaching of both dyes in each trace. Other possibilities include selecting the darkest spots in the illuminated area and subtracting an average background time trace from the data, or using a local background, for example, with a mask around the particle. The latter two options have the advantage that possible (exponential) background bleaching is also corrected. We did not investigate the influence of the kind of background correction during this study, but a recent study showed that not all background estimators are suitable for samples with a high molecule surface coverage^41^.

To summarize, a correction of the background is very important but can be done very well in different ways.

#### Position-specific excitation correction (optional for TIRF)

The concurrent excitation profiles of both lasers are key for accurate measurements (Supplementary Fig. 5). Experimental variations across the field of view are accounted for by a position-specific normalization:

11 $${{\,}_{\mathrm{(profile)}}}^{\rm{ii}}I_{\rm{Aem|Aex}}\, =\, ^{\rm{ii}}I_{\rm{Aem|Aex}}\frac{{I_{\rm{D}}\left( {{{x}}\prime ,{{y}}\prime } \right)}}{{I_{\rm{A}}\left( {{{x}},{{y}}} \right)}}$$

where $I_{\rm{D}}({{x}}\prime ,{{y}}\prime )$ and $I_{\rm{A}}({{x}},{{y}})$ denote the excitation intensities at corresponding positions in the donor or acceptor image, respectively. Individual excitation profiles are determined as the mean image of a stack of images recorded across a sample chamber with dense dye coverage.

#### Leakage (*α*) and direct excitation (*δ*)

After the background correction, the leakage fraction of the donor emission into the acceptor detection channel and the fraction of the direct excitation of the acceptor by the donor-excitation laser are determined. The correction factor for leakage (*α*) is determined by equation (12), using the FRET efficiency of the donor-only population (“D only” in Fig. 2b,f). The correction factor for direct excitation (*β*) is determined by equation (13) from the stoichiometry of the acceptor-only population (“A only” in Fig. 2b,f).

12 $$\alpha = \frac{{\left\langle {\,{}^{\rm{ii}}E_{\rm{app}}^{\rm{(DO)}}} \right\rangle }}{{1 - \left\langle {\,{}^{\rm{ii}}E_{\rm{app}}^{\rm{(DO)}}} \right\rangle }}$$

13 $${\mathrm{\delta }} = \frac{{\left\langle {\,{}^{\rm{ii}}S_{\rm{app}}^{\rm{(AO)}}} \right\rangle }}{{1 - \left\langle {\,{}^{\rm{ii}}S_{\rm{app}}^{\rm{(AO)}}} \right\rangle }}$$

where ${}^{\rm{ii}}E_{\rm{app}}^{\rm{(DO)}}$ and ${}^{\rm{ii}}S_{\rm{app}}^{\rm{(AO)}}$ are calculated from the background-corrected intensities ^ii^*I* of the corresponding population, donor-only or acceptor-only, respectively. This correction, together with the previous background correction, results in the donor-only population being located at *E* = 0, *S* = 1 and the acceptor-only population at *S* = 0, $E = 0 \ldots 1$. The corrected acceptor fluorescence after donor excitation, $F_{\rm{A|D}}$, is given by equation (14), which yields the updated expressions for the FRET efficiency and stoichiometry, equations (15) and (16), respectively.

14 $$F_{\rm{A|D}} = \,{}^{\rm{ii}}I_{\rm{Aem|Dex}} - \alpha \,{}^{\rm{ii}}I_{\rm{Dem|Dex}} - \delta \,{}^{\rm{ii}}I_{\rm{Aem|Aex}}$$

15 $$\,{}^{\rm{iii}}E_{\rm{app}} = \frac {F_{\rm{A|D}}}{{F_{\rm{A|D}} + \,{}^{\rm{ii}}I_{\rm{Dem|Dex}}}}$$

16 $$\,{}^{\rm{iii}}S_{\rm{app}} = \frac {{F_{\rm{A|D}} + \,{}^{\rm{ii}}I_{\rm{Dem|Dex}}}}{{F_{\rm{A|D}} + \,{}^{\rm{ii}}I_{\rm{Dem|Dex}} + \,{}^{ii}I_{\rm{Aem|Aex}}}}$$

In principle, the leaked donor signal could be added back to the donor emission channel^42^. However, this would require precise knowledge about spectral detection efficiencies, which is not otherwise required, and has no effect on the final accuracy of the measurement. As the determination of *α* and *δ* influences the *γ* and *β* correction in the next step, both correction steps can be repeated in an iterative manner if required (e.g., if the *γ* and *β* factors deviate largely from 1).

#### *γ* and *β* correction factors

Differences in the excitation intensities and cross-section, as well as quantum yields and detection efficiencies, are accounted for by use of the correction factors *γ* and *β*, respectively. If the fluorescence quantum yields do not depend on efficiencies or if such dependence is negligible (homogeneous approximation), mean values of efficiencies $\left\langle {\,{}^{\rm{iii}}E_{\rm{app}}^{\rm{(DA)}}} \right\rangle$ and of stoichiometries $\left\langle {\,{}^{\rm{iii}}S_{\rm{app}}^{\rm{(DA)}}} \right\rangle$ are related by equation (17):

17 $$\,{}^{\rm{iii}}S_{\rm{app}}^{\rm{(DA)}} = \left( {1 + \gamma \beta + \left( {1 - \gamma } \right)\beta \,{}^{\rm{iii}}E_{\rm{app}}^{\rm{(DA)}}} \right)^{ - 1}$$

So, in the homogeneous approximation, *γ* and *β* correction factors can be determined by fitting of FRET populations to the histogram of $\,{}^{\rm{iii}}S_{\rm{app}}^{\rm{(DA)}}$ versus $\,{}^{\rm{iii}}E_{\rm{app}}^{\rm{(DA)}}$ with the line defined by equation (17). As this method relies on the analysis of $\,{}^{\rm{iii}}S_{\rm{app}}^{\rm{(DA)}}$ and $\,{}^{\rm{iii}}E_{\rm{app}}^{\rm{(DA)}}$ values obtained from multiple species, we term this method global *γ*-correction. Such a fit can be performed for all FRET populations together, for any of their subsets, and, in principle, for each single-species population separately (see below). Alternatively, a linear fit of inverse $\langle {\,{}^{\rm{iii}}S_{\rm{app}}^{\rm{(DA)}}} \rangle$ versus $\langle {\,{}^{\rm{iii}}E_{\rm{app}}^{\rm{(DA)}}} \rangle$ with *y*-intercept *a* and slope *b* can be performed.

In this case, $\beta = a + b - 1\,{\mathrm{and}}\,\gamma = \left( {a - 1} \right)/\left( {a + b - 1} \right)$.

Error propagation, however, is more straightforward if equation (17) is used. If there is a complex dependence between properties of dyes and efficiencies, the homogeneous approximation is no longer applicable. In this case, the relationship between $\,{}^{\rm{iii}}S_{\rm{app}}^{\rm{(DA)}}$ and $\,{}^{\rm{iii}}E_{\rm{app}}^{\rm{(DA)}}$ for different populations (or even subpopulations for the same single species) cannot be described by equation (17) with a single *γ* correction factor. Here, *γ* can be determined for a single species. We call this ‘single-species *γ*-correction’. This works only if the efficiency broadening is dominated by distance fluctuations. The reason for this assumption is the dependency of these correction factors on both the stoichiometry and the distance-dependent efficiency. In our study, global and local *γ*-correction yielded similar results. Therefore, the homogeneous approximation, with distance fluctuations as the main cause for efficiency broadening, can be assumed for samples 1 and 2. Systematic variation of the *γ* correction factor yields an error of about 10%.

Alternatively, determination of *γ* and *β* factors can be done trace-wise, as in, for example, msALEX experiments^43^, where the *γ* factor is determined as the ratio of the decrease in acceptor signal and the increase in donor signal after acceptor bleaching. We call such an alternative correction individual *γ*-correction^15^. The analysis of local distributions can provide valuable insights about properties of the studied system.

After *γ* and *β* correction, the corrected donor (acceptor) fluorescence after donor (acceptor) excitation $F_{\rm{D|D}}$ ($F_{\rm{A|A}}$) amounts to

18 $$F_{\rm{D|D}} = \gamma \,{}^{\rm{ii}}I_{\rm{Dem|Dex}}$$

19 $$F_{\rm{A|A}} = \frac{1}{\beta }\,{}^{\rm{ii}}I_{\rm{Aem|Aex}}$$

#### Fully corrected values

Application of all corrections leads to the estimates of real FRET efficiencies *E* and stoichiometries *S* from the background-corrected intensities ^ii^*I*. The explicit expressions of fully corrected FRET efficiency and stoichiometry are

20 $$E = \frac{{\left[ {\,{}^{\rm{ii}}I_{\rm{Aem|Dex}} - \alpha \,{}^{\rm{ii}}I_{\rm{Dem|Dex}} - \delta \,{}^{\rm{ii}}I_{\rm{Aem|Aex}}} \right]}}{{\gamma \left[ {\,{}^{\rm{ii}}I_{\rm{Dem|Dex}}} \right] + \left[ {\,{}^{\rm{ii}}I_{\rm{Aem|Dex}} - \alpha \,{}^{\rm{ii}}I_{\rm{Dem|Dex}} - \delta \,{}^{\rm{ii}}I_{\rm{Aem|Aex}}} \right]}}$$

21 $$S = \frac{{\gamma \left[ {\,{}^{\rm{ii}}I_{\rm{Dem|Dex}}} \right] + \left[ {\,{}^{\rm{ii}}I_{\rm{Aem|Dex}} - \alpha \,{}^{\rm{ii}}I_{\rm{Dem|Dex}} - \delta \,{}^{\rm{ii}}I_{\rm{Aem|Aex}}} \right]}}{{\gamma \left[ {\,{}^{\rm{ii}}I_{\rm{Dem|Dex}}} \right] + \left[ {\,{}^{\rm{ii}}I_{\rm{Aem|Dex}} - \alpha \,{}^{\rm{ii}}I_{\rm{Dem|Dex}} - \delta \,{}^{\rm{ii}}I_{\rm{Aem|Aex}}} \right] + 1/\beta \left[ {\,{}^{\rm{ii}}I_{\rm{Aem|Aex}}} \right]}}$$

Plots of the *E*-versus-*S* histogram are shown in Fig. 2d,h. Now, the FRET population should be symmetric to the line for *S* = 0.5. The donor-only population should still be located at *E* = 0, and the acceptor-only population should be at *S* = 0. Finally, the corrected FRET efficiency histogram is generated from events with a stoichiometry of 0.3 < *S* < 0.7 (histograms in Fig. 2). The expected value of the corrected FRET efficiencies *E* is deduced as the center of a Gaussian fit to the efficiency histogram. This is a good approximation for FRET efficiencies in the range from about 0.1 to 0.9. In theory, the shot-noise limited efficiencies follow a binomial distribution if the photon number per burst is constant. For extreme efficiencies or data with a small average number of photons per burst, the efficiency distribution can no longer be approximated with a Gaussian. In this case and also in the case of efficiency broadening due to distance fluctuations, a detailed analysis of the photon statistics can be useful^38,44–46^.

### Uncertainty in distance due to *R*_0_

According to Förster theory^1^, the FRET efficiency *E* and the distance *R* are related by equation (3). In this study, we focused on the comparison of *E* values across different labs in a blind study. Many excellent reviews have been published on how to determine the Förster radius *R*_0_^16,47,48^, and a complete discussion would be beyond the scope of the current study. In the following, we estimate and discuss the different sources of uncertainty in *R*_0_ by utilizing standard error propagation (see also Supplementary Note 6 and ref. ^26^). *R*_0_ is given by equation (7).

The 6th power of the Förster radius is proportional to the relative dipole orientation factor *κ*^2^, the donor quantum yield ${\mathrm{\Phi }}_{F,\rm{D}}$, the overlap integral *J*, and *n*^−4^, where *n* is the refractive index of the medium:

22 $$R_0^6\sim \kappa ^2 \cdot \Phi _{F,\rm{D}} \cdot J \cdot n^{ - 4}$$

For Fig. 5b, we used a total Förster radius related distance uncertainty of 7%, which is justified by the following estimate. Please note that the error in the dipole orientation factor is always specific for the investigated system, whereas the errors in the donor quantum yield, overlap integral and refractive index are more general, although their mean values do also depend on the environment.

*The refractive index*. Different values for the refractive index in FRET systems have been used historically, but ideally the refractive index of the donor–acceptor intervening medium *n*_im_ should be used. Some experimental studies suggest that the use of the refractive index of the solvent may be appropriate, but this is still open for discussion (see, e.g., the discussion in ref. ^49^).

23 $$R_0^6\left( n \right)\sim n_{\rm{im}}^{ - 4}$$

In the worst case, this value *n*_im_ might be anywhere between the refractive index of the solvent (*n*_water _= 1.33) and a refractive index for the dissolved molecule (*n* < *n*_oil_ = 1.52) (ref. ^50^), that is, *n*_water _< *n*_im_ < *n*_oil_. This would result in a maximum uncertainty of Δ*n*_im _< 0.085. As recommended by Clegg^51^, we used *n*_im _ = 1.40 to minimize this uncertainty (Supplementary Table 6). The distance uncertainty propagated from the uncertainty of the refractive indices can then be assumed to be

24 $${\mathrm{\Delta}}R_0\left( n \right) \approx \frac{4}{6}R_o\frac{{{\mathrm{\Delta }}n_{{\rm{im}}}}}{n} < 0.04 \cdot R_0$$

The donor quantum yield Φ_*F*,D_ is position dependent; therefore we measured the fluoresence lifetimes and quantum yields of the free dye Atto 550 and the 1-hi, 1-mid, and 1-lo donor-only labeled samples (Supplementary Table 2).

In agreement with the work of Sindbert et al.^37^, the uncertainty of the quantum yield is estimated at ΔΦ′_*F*,D_ = 5%, arising from the uncertainties of the Φ_*F*_ values of reference dyes and the precision of the absorption and fluorescence measurements. Thus, the distance uncertainty due to the quantum yield is estimated as

25 $${\mathrm{\Delta }}R_0\left( {\Phi _{F,\rm{D}}} \right) \approx \frac{{R_0}}{6}\frac{{{\mathrm{\Delta }}\Phi _{F,\rm{D}}}}{{\Phi _{F,\rm{D}}}} = 0.01 \cdot R_0$$

The overlap integral *J* was measured for the unbound dyes in solution (Atto 550 and Atto 647N), as well as for samples 1-lo and 1-mid. This resulted in a deviation of about 10% for *J* when we used the literature values for the extinction coefficients. All single-stranded labeled DNA samples used in this study were purified with HPLC columns providing a labeling efficiency of at least 95%. The labeling efficiencies of the single-stranded singly labeled DNA and of the double-stranded singly labeled DNA samples were determined by the ratio of the absorption maxima of the dye and the DNA and were all above 97%. This indicates an error of the assumed exctinction coefficient of less than 3%. Thus, the distance uncertainty due to the overlap spectra and a correct absolute acceptor extinction coefficient can be estimated by equation (26). However, the uncertainty in the acceptor extinction coefficient might be larger for other environments, such as when bound to a protein.

26 $${\mathrm{\Delta }}R_0({J}) \approx \frac{{R_0}}{6}\frac{{{\Delta J}}}{{J}} = 0.025 \cdot R_0$$

In addition to the above uncertainty estimation, the *J***-**related uncertainty can also be obtained through verification of the self-consistency of a *β*-factor network^9^. Finally, we found little uncertainty when we used the well-tested dye Atto 647N. Fluorescence spectra were measured on a Fluoromax4 spectrafluorimeter (Horiba, Germany). Absorbance spectra were recorded on a Cary5000 UV-VIS spectrometer (Agilent, USA) (Supplementary Fig. 6).

#### The *κ*^2^ factor and model assumptions

The uncertainty in the distance depends on the dye model used^22^. Several factors need to be considered, given the model assumptions of unrestricted dye rotation, equal sampling of the entire accessible volume, and the rate inequality *k*_rot_ >> *k*_FRET_ >> *k*_diff_ >> *k*_int_.

First, the use of *κ*^2^ = 2/3 is justified if *k*_rot_ >> *k*_FRET_, because then there is rotational averaging of the dipole orientation during energy transfer. *k*_rot_ is determined from the rotational correlation time *ρ*_1_ < 1 ns, and *k*_FRET_ is determined from the fluorescence lifetimes 1 ns < *τ*_fl _< 5 ns. Hence the condition *k*_rot_ >> *k*_FRET_ is not strictly fulfilled. We estimate the error this introduces into *κ*^2^ from the time-resolved anisotropies of donor and acceptor dyes. If the transfer rate is smaller than the fast component of the anisotropy decay (rotational correlation time) of donor and acceptor, then the combined anisotropy, *r*_C_, is given by the residual donor and acceptor anisotropies ($r_{\rm{D},\infty }$ and $r_{\rm{A},\infty }$, respectively):

27 $$r_{\rm{C}} = \sqrt {r_{\rm{A},\infty }} \sqrt {r_{\rm{D},\infty }}$$

In theory, the donor and the acceptor anisotropy should be determined at the time of energy transfer. If the transfer rate is much slower than the fast component of the anisotropy decay of donor and acceptor, the residual anisotropy can be used (Supplementary Fig. 7)^9^. Also, the steady-state anisotropy values can give an indication of the rotational freedom of the dyes on the relevant time scales, if the inherent effect by the fluorescence lifetimes is taken into account (refer to the Perrin equation, *r*(*τ*) = *r*_0_/(1 + (*τ*/*ϕ*)), where *r* is the observed anisotropy, *r*_0_ is the intrinsic anisotropy of the molecule, *τ* is the fluorescence lifetime, and *ϕ* is the rotational time constant; Supplementary Table 2 and Supplementary Fig. 8).

If the steady-state anisotropy and *r*_C_ are low (<0.2), one can assume (but not prove) sufficient isotropic coupling (rotational averaging), that is, *κ*^2^ = 2/3, with an uncertainty of about 5% (ref. ^9^):

28 $${\mathrm{\Delta }}R_0\left( {\kappa ^2,r_{\rm{C}} < 0.2} \right) \approx 0.05 \cdot R_0$$

#### Spatial sampling

In addition, it is assumed that both dyes remain in a fixed location for the duration of the donor lifetime, that is, *k*_FRET_ >> *k*_diff_, where *k*_diff_ is defined as the inverse of the diffusion time through the complete AV. Recently the diffusion coefficient for a tethered Alexa Fluor 488 dye was determined to be *D* = 10 Å^2^/ns (ref. ^30^). Therefore, *k*_diff_ is smaller than *k*_FRET_. For short distances (<5 Å) the rates become similar, but the effect on the interdye distance distribution within the donor’s lifetime is small, as has been observed in time-resolved experiments. We also assumed that, in the experiment, the efficiencies are averaged over all possible interdye positions. This is the case when *k*_diff _>> *k*_int_, which is a very good assumption for TIRF experiments with *k*_int_ > 100 ms, and also for confocal experiments with *k*_int_ values around 1 ms.

#### Overall uncertainty in *R*_0_

Time-resolved anisotropy measurements of samples 1 and 2 resulted in combined anisotropies less than 0.1. Therefore, we assumed isotropic coupling to obtain *R*_MP_. The *R*_MP_ values matched the model distances very well, further supporting these assumptions. Finally, an experimental study of *κ*^2^ distributions also yielded typical errors of 5% (ref. ^37^).

The overall uncertainty for the Förster radius would then result in

29 $${\mathrm{\Delta }}R_0\left( {n^{ - 4},\Phi _{F,\rm{D}},J,\kappa ^2} \right) = \sqrt {{\mathrm{\Delta }}R_0(n)^2 + {\mathrm{\Delta }}R_0\left( {\Phi _{F,\rm{D}}} \right)^2 + {\mathrm{\Delta }}R_0(J)^2 + {\mathrm{\Delta }}R_0(\kappa ^2)^2} \lesssim 0.07 \cdot R_0$$

The absolute values determined for this study are summarized in Supplementary Table 6. Please note that the photophysical properties of dyes vary in different buffers and when the dyes are attached to different biomolecules. Therefore, all four quantities that contribute to the uncertainty in *R*_0_ should be measured for the system under investigation. When supplier values or values from other studies are applied, the uncertainty can be much larger. The values specified here could be further evaluated and tested in another blind study.

#### Comparing distinct dye pairs

Even though time-resolved fluorescence anisotropy can show whether dye rotation is fast, the possibility of dyes interacting with the DNA cannot be fully excluded. Thus, it is not clear whether the dye molecule is completely free to sample the computed AV (free diffusion), or whether there are sites of attraction (preferred regions) or sites of repulsion (disallowed regions). To validate the model assumption of a freely rotating and diffusing dye, we define the ratio *R*_rel_ for two apparent distances measured with the same dye pair (e.g., when comparing the low- to the mid-distance):

30 $$R_{\rm{rel}} = \frac{{R_{\left\langle E \right\rangle ,{\rm{lo}}}}}{{R_{\left\langle E \right\rangle ,{\rm{mid}}}}} = \frac{{R_{0,{\rm{lo}}}}}{{R_{0,{\rm{mid}}}}}\root {6} \of {{\frac{{1/E_{\rm{lo}} - 1}}{{1/E_{\rm{mid}} - 1}}}} = \root {6} \of {{\frac{{\kappa _{\rm{lo}}^2\Phi _{\rm{D,lo}}J_{\rm{lo}}n_{\rm{mid}}^4}}{{\kappa _{\rm{mid}}^2\Phi _{\rm{D,mid}}J_{\rm{mid}}n_{\rm{lo}}^4}}}}\root {6} \of {{\frac{{1/E_{\rm{lo}} - 1}}{{1/E_{\rm{mid}} - 1}}}} = f \cdot \root {6} \of {{\frac{{1/E_{\rm{lo}} - 1}}{{1/E_{\rm{mid}} - 1}}}}$$

For comparison of the other apparent distances, the ratio is adapted accordingly. Computed values relative to the mid-distance are shown in Supplementary Table 4. Note that *R*_rel_ values are (quasi) independent of *R*_0_ for the following reasons: first, the donor positions in the lo, mid, and hi constructs are kept constant between samples 1, 2, 3 and 4, respectively. Therefore, the following assumptions can be made: (i) the ratios of the donor quantum yields are identical; (ii) the ratios of the spectral overlaps *J* for the lo, mid, and hi samples of one and the same dye pair should be the same; (iii) for a given geometry (Fig. 1) the refractive indices *n*_im_ of the medium between the dyes should also be very similar; and (iv) the ratios of the orientation factors *κ*² should be nearly equal, as the measured donor anisotropies are low for the lo, mid, and hi positions. Second, the acceptor extinction coefficients cancel each other out, as the acceptor is at the same position for the lo, mid, and hi constructs within a sample. Thus, the different dye pairs and the model used in this study should all give similar values for *R*_rel_. Therefore, we compared the *R*_rel_ values for different dye pairs to determine whether for a particular dye pair the model assumptions are in agreement with the experimental data. Given our relative error in the determined distance of at most 2.8% (Fig. 5a), this is actually the case for all dye pairs investigated.

### Reporting Summary

Further information on research design is available in the Nature Research Reporting Summary linked to this article.

### Code availability

All custom code used herein is available from the authors upon reasonable request.

### Data availability

All data are available from the corresponding authors upon reasonable request. The raw data for Fig. 2 are available at Zenodo (10.5281/zenodo.1249497).

## Methods

Methods, including statements of data availability and any associated accession codes and references, are available at 10.1038/s41592-018-0085-0.

## Supplementary Information

### Integrated supplementary information

Supplementary Figure 1 DNA sample and utilized dyes. Left: DNA model with dye accessible volumes of the donor (blue) and acceptor (red) that were used in this study, indicating lo-, mid- and hi-FRET samples. Right: Structural formula of the dyes used in this study. Based on dyes from Molecular Probes / Thermo Fisher Scientific (Waltham, USA) and Atto-tec (Siegen, D).

Supplementary Figure 2 FRET efficiencies of all labs for all measured samples as indicated. FRET efficiencies of all labs for all measured samples as indicated. Sample 1 to 4 (see Supplementary Table 1 and Supplementary Note 1) are color coded (red, blue, green, yellow) for all data points from intensity-based techniques. For a table of *R*_*E*_ and *R*_*MP*_ and sample size for these measurements see Supplementary Table 4. Ensemble lifetime, single molecule lifetime and phasor approach derived data is shown in black. The FRET efficiencies (means and s.d.) for these measurements (depicted in black, sample size n) are: *E*_1*a*_ = 0.21±0.05 (n = 6); *E*_1*b*_ = 0.51±0.08 (n = 6); *E*_2*a*_ = 0.25±0.06 (n = 4); *E*_2*b*_ = 0.59±0.07 (n = 4); *E*_3*a*_ = 0.10±0.04 (n = 3); *E*_3*b*_ = 0.26±0.03 (n = 3); *E*_4*a*_ = 0.12±0.10 (n = 3); *E*_1*a*_ = 0.42±0.02 (n = 3). The left figure depicts all measurements from the main study, the right figure depicts all measurements from the later measurements of two additional samples (1-hi, 2-hi).

Supplementary Figure 3 Schematics of a typical confocal setup with alternating laser excitation and pulsed interleaved excitation. Schematics of a typical confocal setup with alternating laser excitation / pulsed interleaved excitation and color-sensitive detection. The most important elements are specified: Objective (O), dichroic mirror (DM), pinhole (P), spectral filter (F), avalanche photo diode (APD) and electronic micro- or picosecond synchronization of laser pulses and single photon counting (Sync). Elements used for the correction factors in Table 2 (main text) were: F34-641 Laser clean-up filter z 640/10 (right after Laser 640 nm); DM_1_: F43-537 laser beam splitter z 532 RDC ; DM_2_: F53-534 Dual Line beam splitter z 532/633; DM_3_: F33-647 laser- laser beam splitter 640 DCXR; F_G_: F37-582 Brightline HC 582/75; F_R_: F47-700 ET Bandpass 700/75; Objective: Cfi plan apo VC 60xWI, NA1.2; Detectors: MPD Picoquant (green), tau-SPAD, Picoquant (red); Pinholes: 100 µm; ; Laser power at sample: ≈ 100 µW; Beam diameter ≈ 2 mm; Diffusion time of Atto550 and Atto647N around 0.42 ms and 0.50 ms, respectively. For details on all used setups and analysis software, see Supplementary Note 8.

Supplementary Figure 4 Schematic designs of an objective-type and a prism-type TIRF setup. **a**, Objective. **b**, Prism. Green and red lasers are used to excite donor and acceptor dyes, respectively. M, mirror. L, lens. DM, dichroic mirror. Obj, objective. AD, achromatic doublet lens. Sl, tunable slit. F, filters. Det, detector (e.g. electron multiplying charge-coupled device camera, EMCCD). The inset shows a side view of the objective with the out-of-plane (45°) mirror below. SC, sample chamber. Ir, iris. St, translation stage, Pr, prism. The dashed black line in (a) indicates the *on-axis* path to the objective, in contrast to the displayed *off-axis* path for TIR illumination. Elements used for the correction factors in Table 2 (main text) were: Dichroic before objective: F53-534 (AHF), Dichroics in detection: F33-726 and F33-644 (AHF). Band pass filters in detection: BP F39-572 and BP F37-677 (AHF). SI: SP40 (Owis), Objective: CFI Apo TIRF 100x, NA 1.49 (Nikon). Camera: EMCCD, iXonUltra, Andor. Lasers: 532nm, Compass 215M (Coherent) and 635nm, Lasiris (Stoker Yale). Note that we have used a Dichroic in the fluorescence excitation and emission path that reflects the higher wavelength, but this does not have any effect on the FRET efficiency measurement and related determination of correction factors. For details on all used setups and analysis software, see Supplementary Note 8.

Supplementary Figure 5 Correcting for differences in the excitation intensity in TIRF microscopy. Accounting for the differences in the excitation intensity profiles of the green and red laser across the field of view. The individual excitation profiles are determined as the mean image of a stack of images recorded while moving across a dense layer of dyes. In contrast to the uncorrected case (“before”), a position specific normalization creates narrower and more symmetric SE-populations (“after”). The standard corrections described in the main text are performed subsequently.

Supplementary Figure 6Computation of the spectral overlap integral *J*.Computation of the spectral overlap integral J for the FRET pair Atto550-Atto647N in sample 1. Normalized donor fluorescence and acceptor absorption spectra normalized to the maximum (left scale). Spectral overlap density *j*(*λ*)(right scale) to compute the spectral overlap integral *J*[cm^−1^M^−1^nm^4^]with $J = \mathop {\smallint }\limits_0^\infty {\mathrm{j}}\left( \lambda \right){\mathrm{d}}\lambda$ and $j(\lambda ) = \bar F_D\left( \lambda \right)\varepsilon _A\left( \lambda \right)\lambda ^4$. The extinction coefficient *ε*_*A*_ of Atto647N was assumed to be 150000M^−1^cm^−1^ at the maximum as provided by the manufacturer. The donor fluorescence and the acceptor absorption spectra were recorded in two laboratories in at least three independent experiments. Spectra with a flat baseline were selected. The computation was performed once.

Supplementary Figure 7 Time-resolved anisotropies and FRET. The time-resolved anisotropies of dyes bound to a larger object (e.g. DNA or protein) normally consist of a fast decay from rotational relaxation of the dipole (left) and of a slow decay from translational relaxation (right). *τ*_*ET*_ = 1/*k*_*FRET*_: time of energy transfer; *r*_*A*,∞_: residual anisotropy of dye A. (Figure from ref. 1). The data exemplarily shown is from a single measurement.^1^ Hellenkamp, B., Wortmann, P., Kandzia, F., Zacharias, M. & Hugel, T. Multidomain Structure and Correlated Dynamics Determined by Self-Consistent FRET Networks. Nat. Meth. 14, 174-180 (2017).

Supplementary Figure 8 Visualizations of different averages for efficiencies according to different fluorophore dynamics. (a) Dynamic average, which applies in the case of the fluorophore movements being faster than the rate of energy transfer. There the rate of energy transfer has to be calculated taking into account the average over all possible distances and orientations. (b) Intermediate case, called the isotropic average, where the orientational variation of the fluorophores is faster than the rate of energy transfer while the positional variation is slower (c) Static case, where the fluorophore movements are much slower than the rate of energy transfer. In this case each distance and respective fluorophore orientation has to be taken into account with its individual transfer efficiency. These efficiencies then are averaged by the measurement process. (Figure from ref. ^2^). 2 *Wozniak, A. K., Schröder, G. F., Grubmüller, H., Seidel, C. A. M. & Oesterhelt, F. Single-Molecule FRET Measures Bends and Kinks in DNA. Proc. Natl Acad. Sci. USA 105, 18337-18342 (2008).*

### Supplementary information

Supplementary Text and Figures Supplementary Figures 1–8, Supplementary Tables 1–6, and Supplementary Notes 1–8

Reporting Summary
