# Supplementary figures and images for: Precision and accuracy of single-molecule FRET measurements—a multi-laboratory benchmark study
Source: Nat Methods. 2018 Aug 31;15(9):669–76. doi: 10.1038/s41592-018-0085-0 (PMC6121742; doi:10.1038/s41592-018-0085-0)

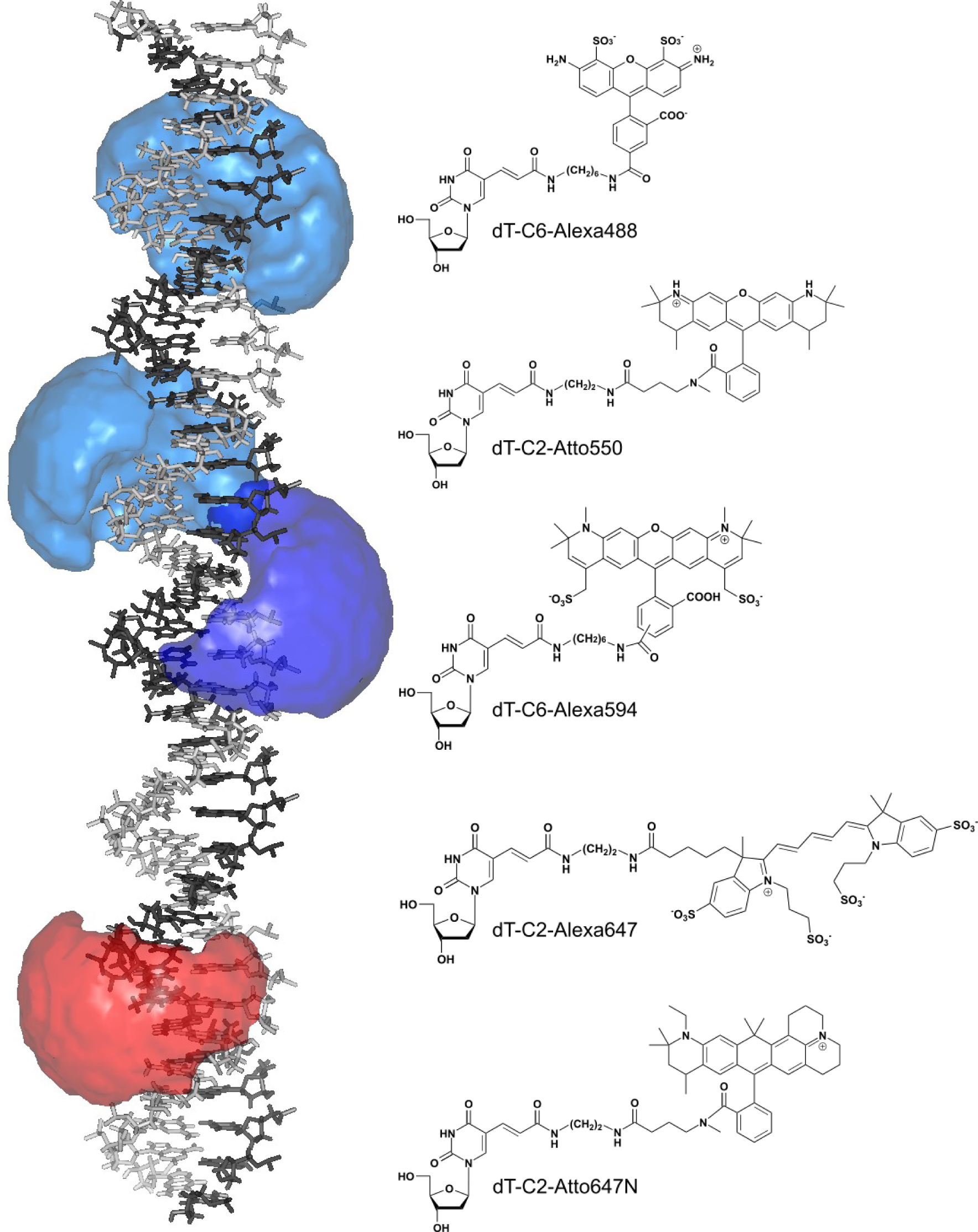

Supplement: DNA sample and utilized dyes. — Left: DNA model with dye accessible volumes of the donor (blue) and acceptor (red) that were used in this study, indicating lo-, mid- and hi-FRET samples. Right: Structural formula of the dyes used in this study. Based on dyes from Molecular Probes / Thermo Fisher Scientific (Waltham, USA) and Atto-tec (Siegen, D). [file 41592_2018_85_Fig6_ESM.jpg]

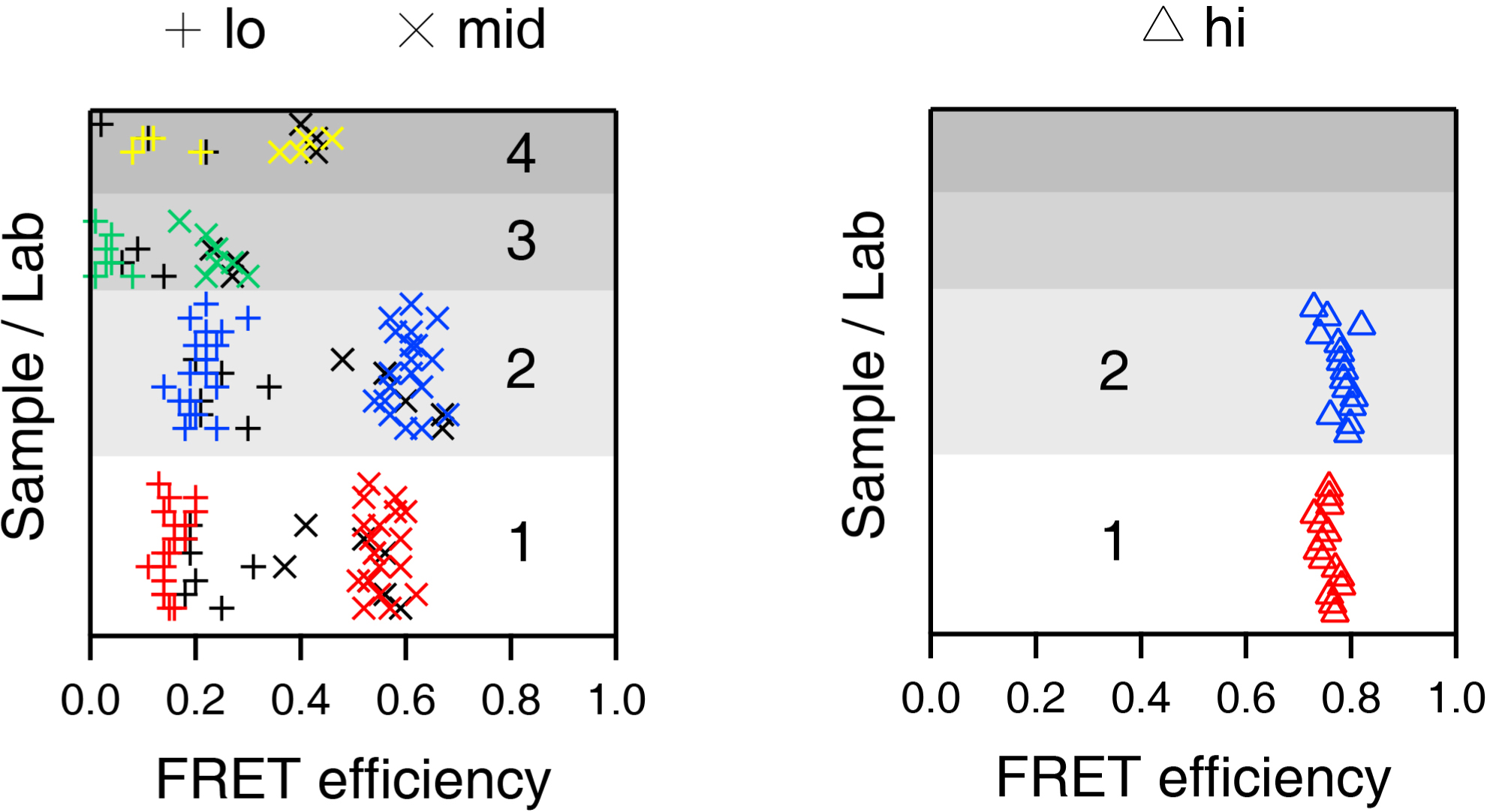

Supplement: FRET efficiencies of all labs for all measured samples as indicated. — Sample 1 to 4 (see Supplementary Table 1 and Supplementary Note 1) are color coded (red, blue, green, yellow) for all data points from intensity-based techniques. For a table of RE and RMP and sample size for these measurements see Supplementary Table 4. Ensemble lifetime, single molecule lifetime and phasor approach derived data is shown in black. The FRET efficiencies (means and s.d.) for these measurements (depicted in black, sample size n) are: E1a = 0.21±0.05 (n = 6); E1b = 0.51±0.08 (n = 6); E2a = 0.25±0.06 (n = 4); E2b = 0.59±0.07 (n = 4); E3a = 0.10±0.04 (n = 3); E3b = 0.26±0.03 (n = 3); E4a = 0.12±0.10 (n = 3); E1a = 0.42±0.02 (n = 3). The left figure depicts all measurements from the main study, the right figure depicts all measurements from the later measurements of two additional samples (1-hi, 2-hi). [file 41592_2018_85_Fig7_ESM.jpg]

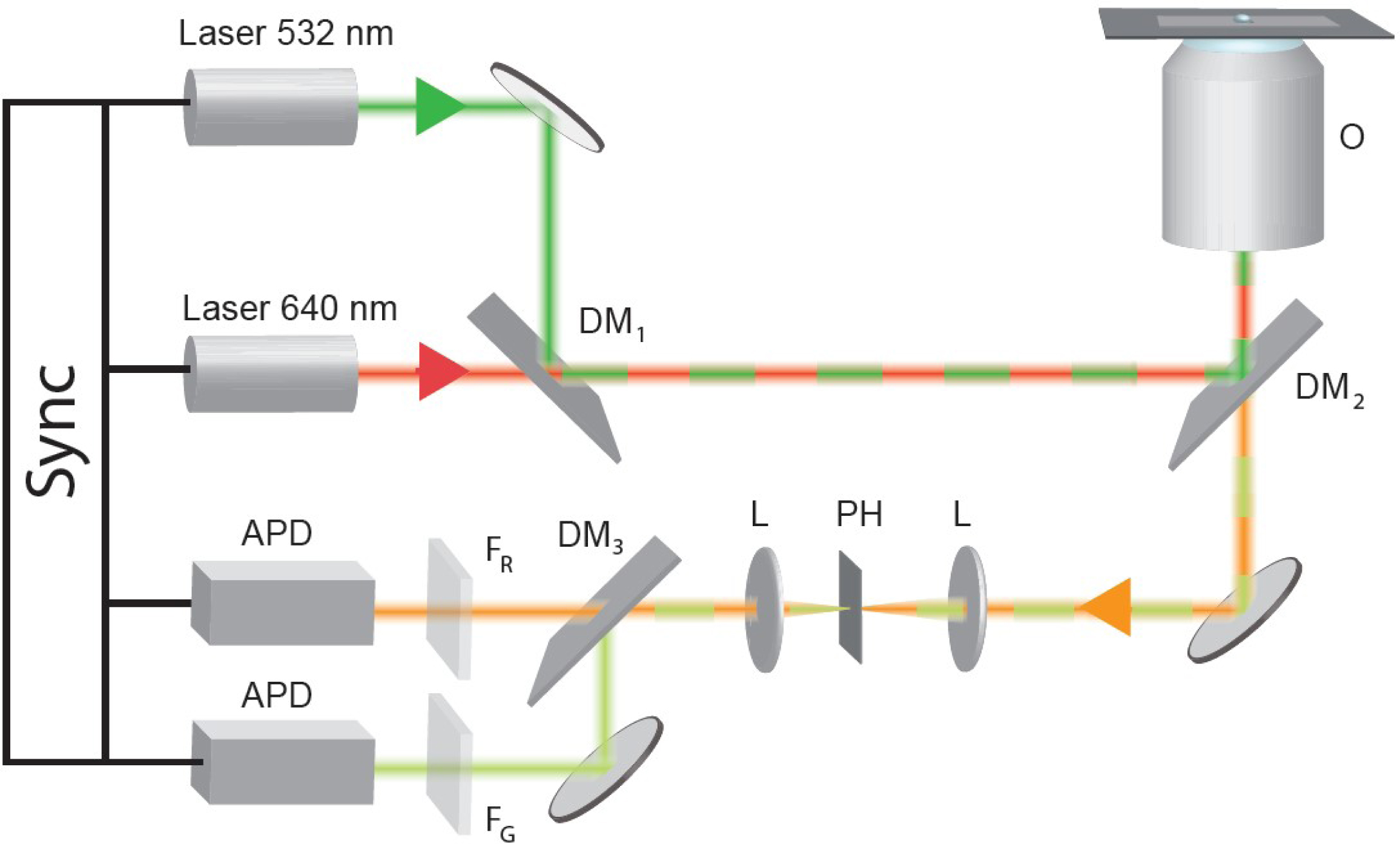

Supplement: Schematics of a typical confocal setup with alternating laser excitation and pulsed interleaved excitation. — Schematics of a typical confocal setup with alternating laser excitation / pulsed interleaved excitation and color-sensitive detection. The most important elements are specified: Objective (O), dichroic mirror (DM), pinhole (P), spectral filter (F), avalanche photo diode (APD) and electronic micro- or picosecond synchronization of laser pulses and single photon counting (Sync). Elements used for the correction factors in Table 2 (main text) were: F34-641 Laser clean-up filter z 640/10 (right after Laser 640 nm); DM1: F43-537 laser beam splitter z 532 RDC ; DM2: F53-534 Dual Line beam splitter z 532/633; DM3: F33-647 laser- laser beam splitter 640 DCXR; FG: F37-582 Brightline HC 582/75; FR: F47-700 ET Bandpass 700/75; Objective: Cfi plan apo VC 60xWI, NA1.2; Detectors: MPD Picoquant (green), tau-SPAD, Picoquant (red); Pinholes: 100 µm; ; Laser power at sample: ≈ 100 µW; Beam diameter ≈ 2 mm; Diffusion time of Atto550 and Atto647N around 0.42 ms and 0.50 ms, respectively. For details on all used setups and analysis software, see Supplementary Note 8. [file 41592_2018_85_Fig8_ESM.jpg]

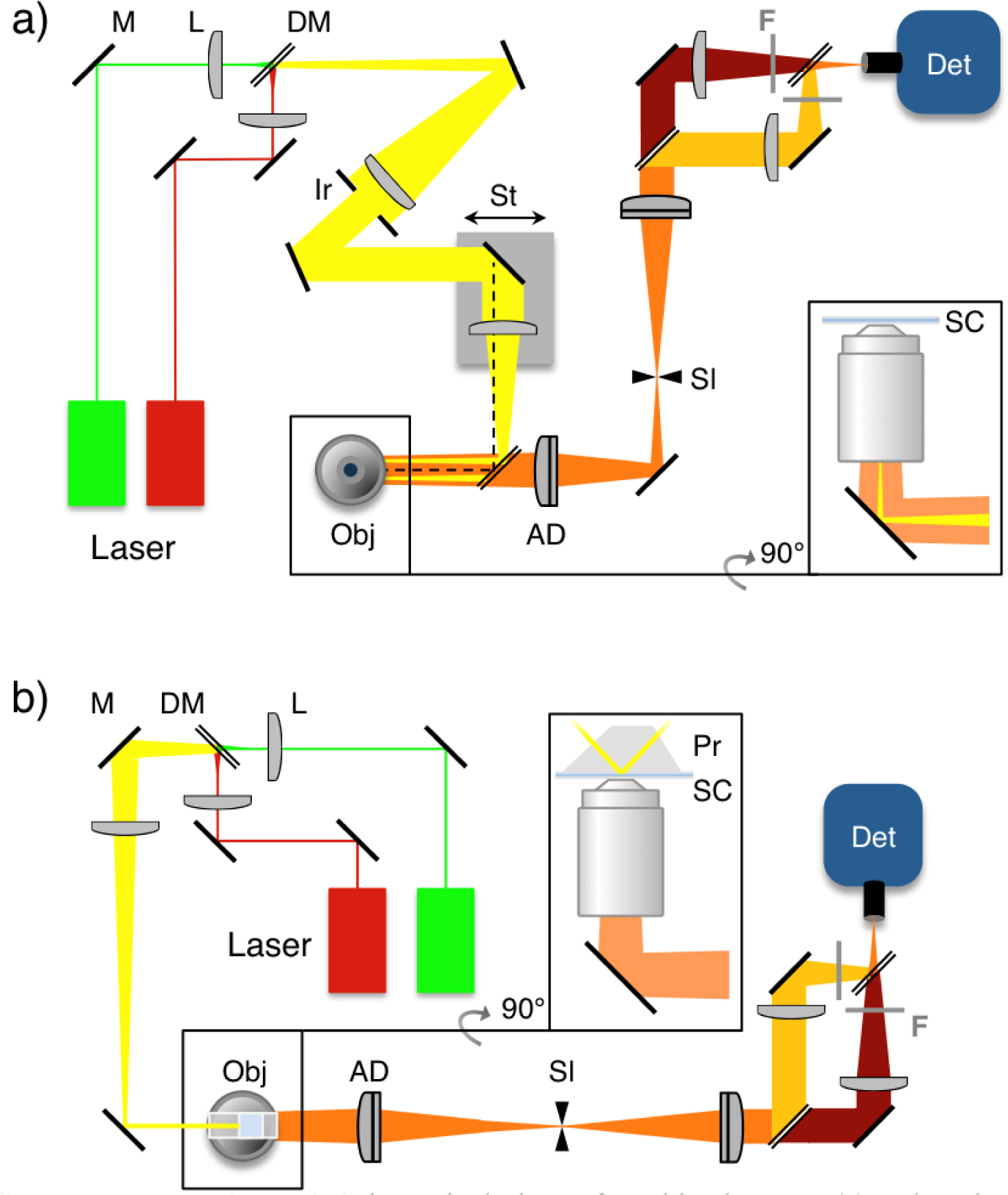

Supplement: Schematic designs of an objective-type and a prism-type TIRF setup. — a, Objective. b, Prism. Green and red lasers are used to excite donor and acceptor dyes, respectively. M, mirror. L, lens. DM, dichroic mirror. Obj, objective. AD, achromatic doublet lens. Sl, tunable slit. F, filters. Det, detector (e.g. electron multiplying charge-coupled device camera, EMCCD). The inset shows a side view of the objective with the out-of-plane (45°) mirror below. SC, sample chamber. Ir, iris. St, translation stage, Pr, prism. The dashed black line in (a) indicates the on-axis path to the objective, in contrast to the displayed off-axis path for TIR illumination. Elements used for the correction factors in Table 2 (main text) were: Dichroic before objective: F53-534 (AHF), Dichroics in detection: F33-726 and F33-644 (AHF). Band pass filters in detection: BP F39-572 and BP F37-677 (AHF). SI: SP40 (Owis), Objective: CFI Apo TIRF 100x, NA 1.49 (Nikon). Camera: EMCCD, iXonUltra, Andor. Lasers: 532nm, Compass 215M (Coherent) and 635nm, Lasiris (Stoker Yale). Note that we have used a Dichroic in the fluorescence excitation and emission path that reflects the higher wavelength, but this does not have any effect on the FRET efficiency measurement and related determination of correction factors. For details on all used setups and analysis software, see Supplementary Note 8. [file 41592_2018_85_Fig9_ESM.jpg]

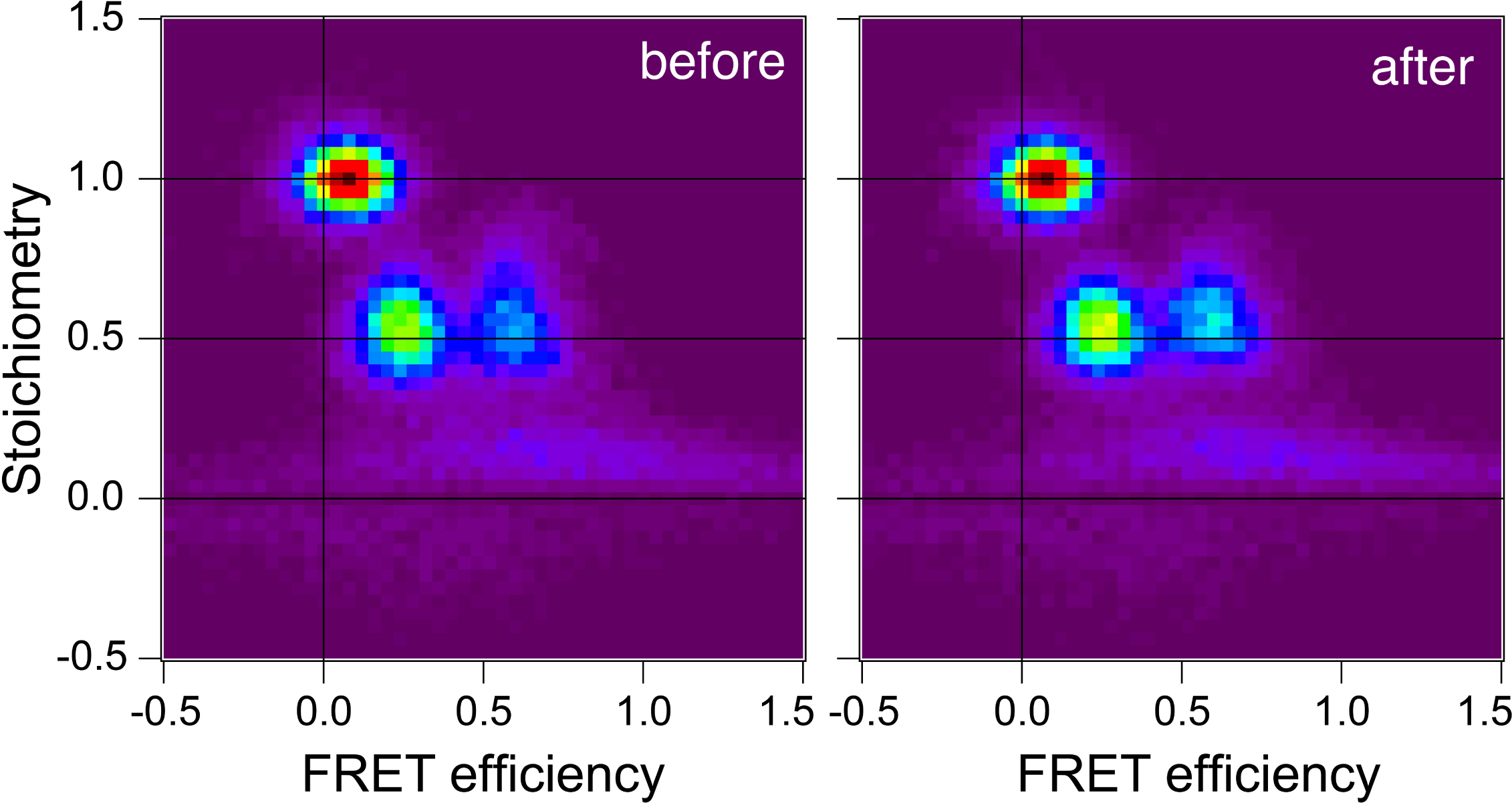

Supplement: Correcting for differences in the excitation intensity in TIRF microscopy. — Accounting for the differences in the excitation intensity profiles of the green and red laser across the field of view. The individual excitation profiles are determined as the mean image of a stack of images recorded while moving across a dense layer of dyes. In contrast to the uncorrected case (“before”), a position specific normalization creates narrower and more symmetric SE-populations (“after”). The standard corrections described in the main text are performed subsequently. [file 41592_2018_85_Fig10_ESM.jpg]

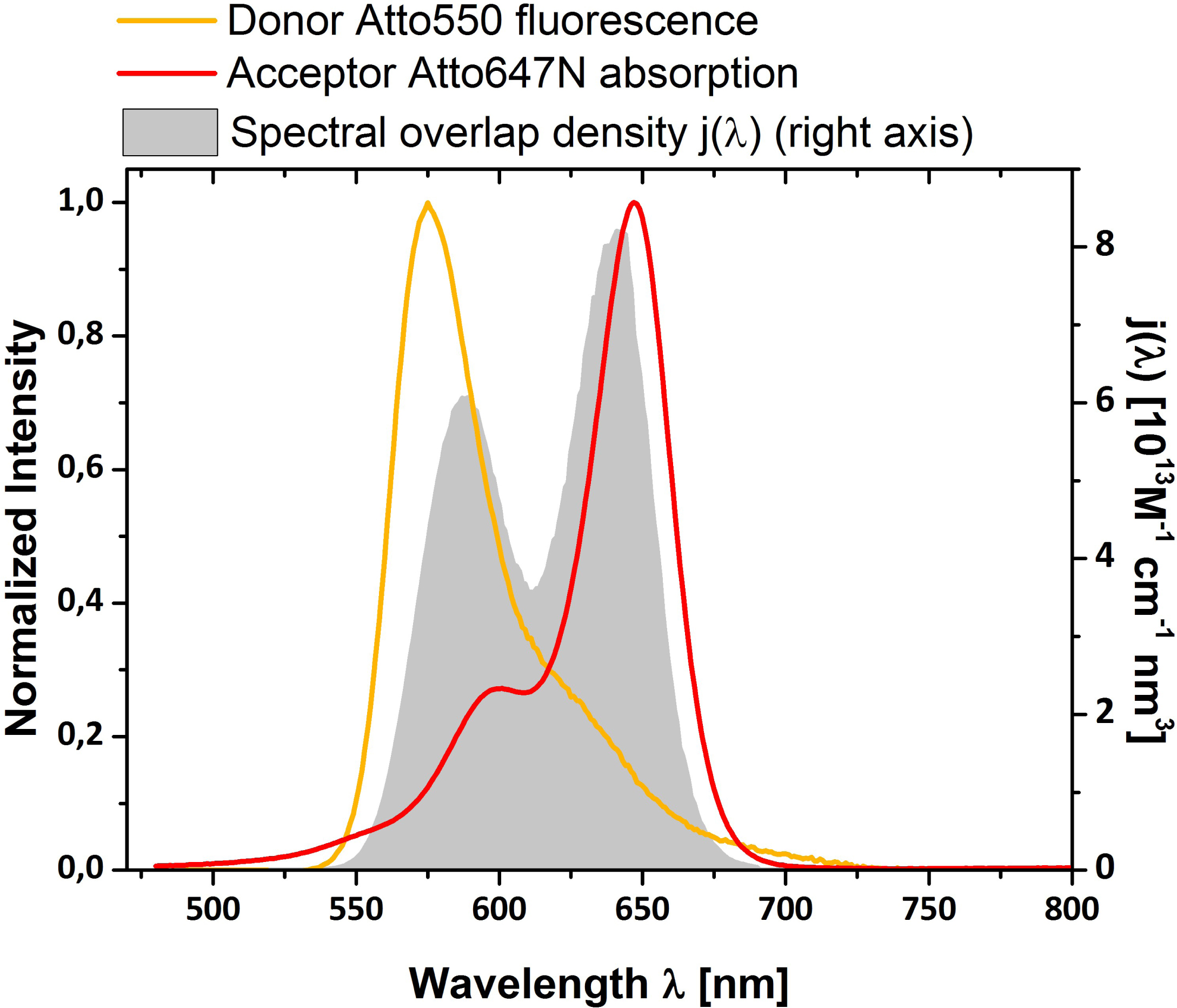

Supplement: Computation of the spectral overlap integral J. — Computation of the spectral overlap integral J for the FRET pair Atto550-Atto647N in sample 1. Normalized donor fluorescence and acceptor absorption spectra normalized to the maximum (left scale). Spectral overlap density j(λ)(right scale) to compute the spectral overlap integral J[cm−1M−1nm4]with $J = \mathop {\smallint }\limits_0^\infty {\mathrm{j}}\left( \lambda \right){\mathrm{d}}\lambda$J= ∫0∞jλdλ and $j(\lambda ) = \bar F_D\left( \lambda \right)\varepsilon _A\left( \lambda \right)\lambda ^4$j(λ)=F¯DλεAλλ4. The extinction coefficient εA of Atto647N was assumed to be 150000M−1cm−1 at the maximum as provided by the manufacturer. The donor fluorescence and the acceptor absorption spectra were recorded in two laboratories in at least three independent experiments. Spectra with a flat baseline were selected. The computation was performed once. [file 41592_2018_85_Fig11_ESM.jpg]

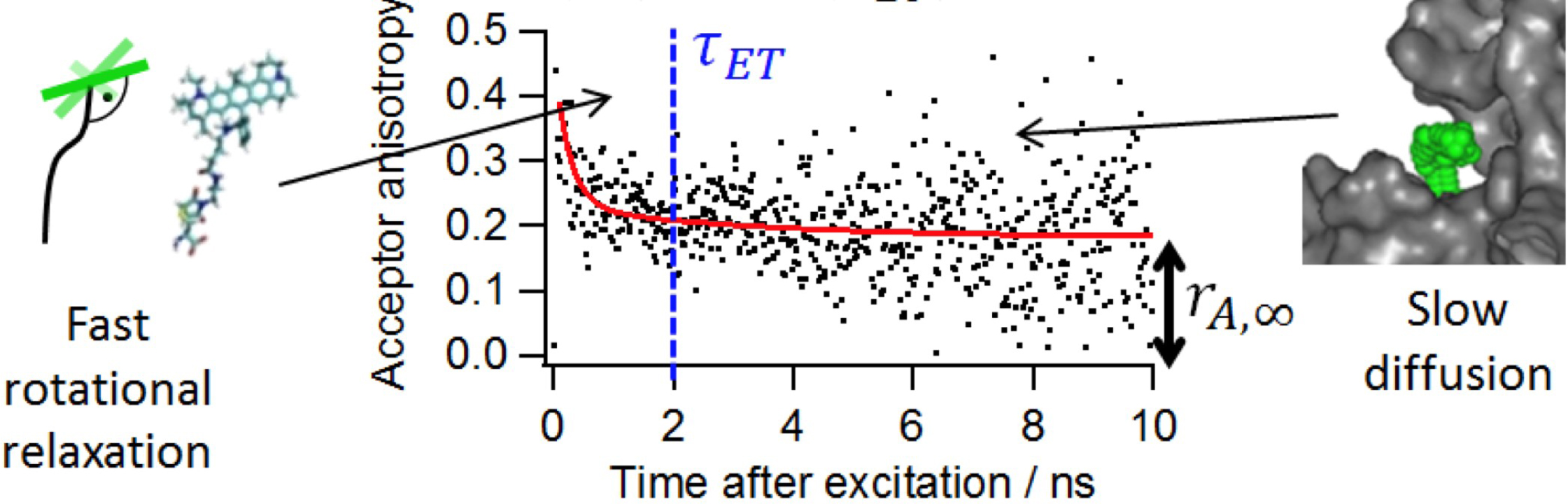

Supplement: Time-resolved anisotropies and FRET. — The time-resolved anisotropies of dyes bound to a larger object (e.g. DNA or protein) normally consist of a fast decay from rotational relaxation of the dipole (left) and of a slow decay from translational relaxation (right). τET = 1/kFRET: time of energy transfer; rA,∞: residual anisotropy of dye A. (Figure from ref. 1). The data exemplarily shown is from a single measurement.1 Hellenkamp, B., Wortmann, P., Kandzia, F., Zacharias, M. & Hugel, T. Multidomain Structure and Correlated Dynamics Determined by Self-Consistent FRET Networks. Nat. Meth. 14, 174-180 (2017). [file 41592_2018_85_Fig12_ESM.jpg]

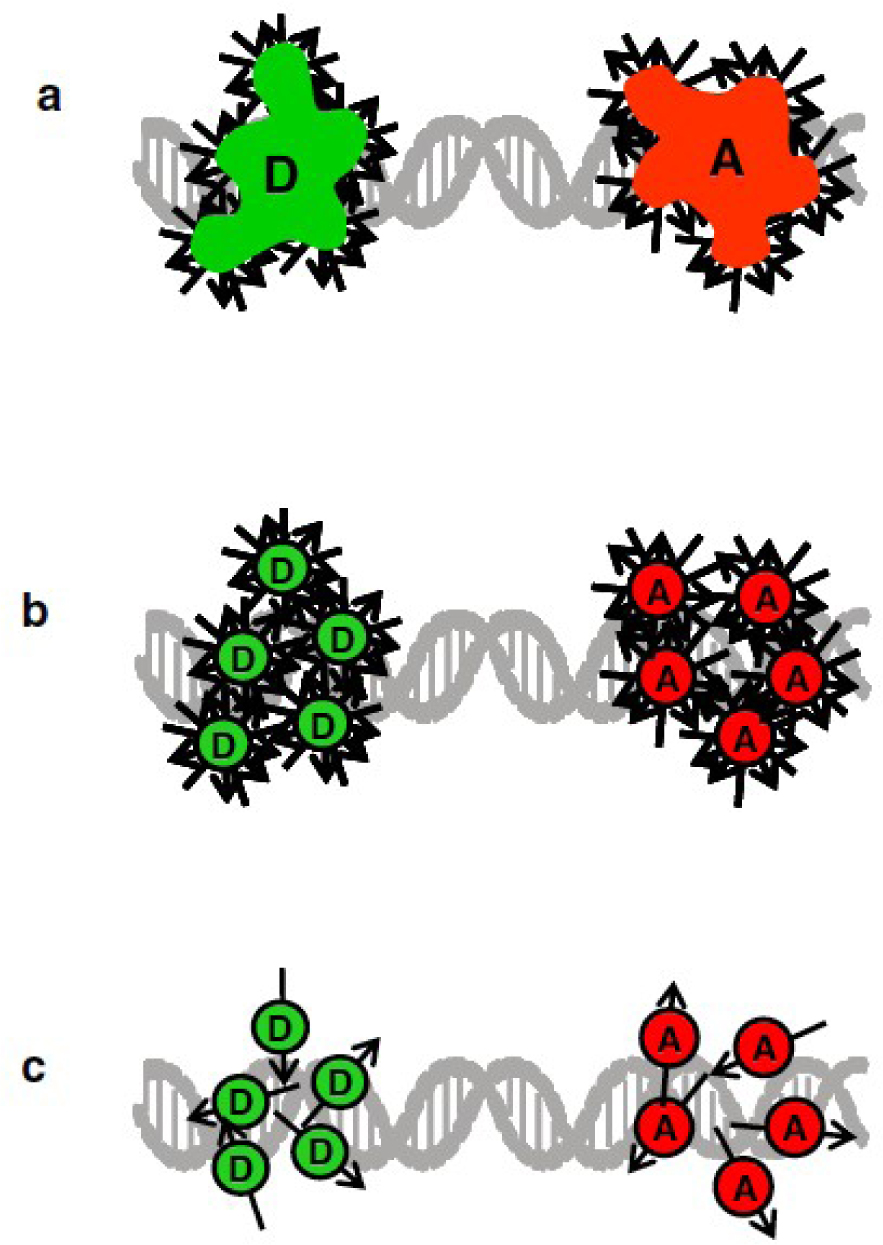

Supplement: Visualizations of different averages for efficiencies according to different fluorophore dynamics. — (a) Dynamic average, which applies in the case of the fluorophore movements being faster than the rate of energy transfer. There the rate of energy transfer has to be calculated taking into account the average over all possible distances and orientations. (b) Intermediate case, called the isotropic average, where the orientational variation of the fluorophores is faster than the rate of energy transfer while the positional variation is slower (c) Static case, where the fluorophore movements are much slower than the rate of energy transfer. In this case each distance and respective fluorophore orientation has to be taken into account with its individual transfer efficiency. These efficiencies then are averaged by the measurement process. (Figure from ref. 2). 2 Wozniak, A. K., Schröder, G. F., Grubmüller, H., Seidel, C. A. M. & Oesterhelt, F. Single-Molecule FRET Measures Bends and Kinks in DNA. Proc. Natl Acad. Sci. USA 105, 18337-18342 (2008). [file 41592_2018_85_Fig13_ESM.jpg]
