# Supplementary material for: Precision and accuracy of single-molecule FRET measurements—a multi-laboratory benchmark study
Source: Nat Methods. 2018 Aug 31;15(9):669–76. doi: 10.1038/s41592-018-0085-0 (PMC6121742; doi:10.1038/s41592-018-0085-0)
Supplement: Supplementary file 9 — Supplementary Figures 1–8, Supplementary Tables 1–6, and Supplementary Notes 1–8 [file 41592_2018_85_MOESM1_ESM.pdf]

nature methods

SUPPLEMENTARY INFORMATION
https://doi.org/10.1038/s41592-018-0085-0

In the format provided by the authors and unedited.

Precision and accuracy of single-molecule FRET measurements—a multi-laboratory benchmark study

Björn Hellenkamp[1,2,33], Sonja Schmid[1,3,33], Olga Doroshenko[4], Oleg Opanasyuk[4], Ralf Kühnemuth[4], Soheila Rezaei Adariani[5], Benjamin Ambrose[6], Mikayel Aznauryan[7], Anders Barth[8], Victoria Birkedal[7], Mark E. Bowen[9], Hongtao Chen[10], Thorben Cordes[11,12], Tobias Eilert[13], Carel Fijen[14], Christian Gebhardt[12], Markus Götz[1], Giorgos Gouridis[11,12], Enrico Gratton[10], Taekjip Ha[15], Pengyu Hao[16], Christian A. Hanke[4], Andreas Hartmann[17], Jelle Hendrix[18,19], Lasse L. Hildebrandt[7], Verena Hirschfeld[20], Johannes Hohlbein[14,21], Boyang Hua[15], Christian G. Hübner[20], Eleni Kallis[13], Achillefs N. Kapanidis[22], Jae-Yeol Kim[23], Georg Krainer[17,24], Don C. Lamb[8], Nam Ki Lee[23], Edward A. Lemke[25,26,27], Brié Levesque[9], Marcia Levitus[28], James J. McCann[9], Nikolaus Naredi-Rainer[8], Daniel Nettels[29], Thuy Ngo[15], Ruoyi Qiu[16], Nicole C. Robb[22], Carlheinz Röcker[13], Hugo Sanabria[5], Michael Schlierf[17], Tim Schröder[30], Benjamin Schuler[29], Henning Seidel[20], Lisa Streit[13], Johann Thurn[1], Philip Tinnefeld[30,31], Swati Tyagi[27], Niels Vandenberk[18], Andrés Manuel Vera[30], Keith R. Weninger[16], Bettina Wünsch[31], Inna S. Yanez-Orozco[5], Jens Michaelis[13]*, Claus A. M. Seidel[4]*, Timothy D. Craggs[6,22]* and Thorsten Hugel[1,32]*

[1]Institute of Physical Chemistry, University of Freiburg, Freiburg im Breisgau, Germany. [2]Engineering and Applied Sciences, Columbia University, New York, NY, USA. [3]Department of Bionanoscience, Kavli Institute of Nanoscience Delft, Delft University of Technology, Delft, the Netherlands. [4]Molecular Physical Chemistry, Heinrich-Heine-Universität Düsseldorf, Düsseldorf, Germany. [5]Department of Physics and Astronomy, Clemson University, Clemson, SC, USA. [6]Department of Chemistry, University of Sheffield, Sheffield, UK. [7]Interdisciplinary Nanoscience Center (iNANO) and Department of Chemistry, Aarhus University, Aarhus, Denmark. [8]Physical Chemistry, Department of Chemistry, Nanosystems Initiative Munich (NIM), Center for Integrated Protein Science Munich (CiPSM) and Center for Nanoscience (CeNS), Ludwig-Maximilians-Universität München, Munich, Germany. [9]Department of Physiology & Biophysics, Stony Brook University, Stony Brook, NY, USA. [10]Department of Biomedical Engineering, University of California, Irvine, Irvine, CA, USA. [11]Molecular Microscopy Research Group, Zernike Institute for Advanced Materials, University of Groningen, Groningen, the Netherlands. [12]Physical and Synthetic Biology, Faculty of Biology, Ludwig-Maximilians-Universität München, Planegg-Martinsried, Germany. [13]Institute for Biophysics, Ulm University, Ulm, Germany. [14]Laboratory of Biophysics, Wageningen University & Research, Wageningen, the Netherlands. [15]Department of Biomedical Engineering, John Hopkins University, Baltimore, MD, USA. [16]Department of Physics, North Carolina State University, Raleigh, NC, USA. [17]B CUBE—Center for Molecular Bioengineering, TU Dresden, Dresden, Germany. [18]Laboratory for Photochemistry and Spectroscopy, Department of Chemistry, University of Leuven, Leuven, Belgium. [19]Dynamic Bioimaging Lab, Advanced Optical Microscopy Center and Biomedical Research Institute, Hasselt University, Hasselt, Belgium. [20]Institute of Physics, University of Lübeck, Lübeck, Germany. [21]Microspectroscopy Research Facility Wageningen, Wageningen University & Research, Wageningen, the Netherlands. [22]Gene Machines Group, Clarendon Laboratory, Department of Physics, University of Oxford, Oxford, UK. [23]School of Chemistry, Seoul National University, Seoul, South Korea. [24]Molecular Biophysics, Technische Universität Kaiserslautern (TUK), Kaiserslautern, Germany. [25]Departments of Biology and Chemistry, Pharmacy and Geosciences, Johannes Gutenberg-University Mainz, Mainz, Germany. [26]Institute of Molecular Biology (IMB), Mainz, Germany. [27]Structural and Computational Biology Unit, European Molecular Biology Laboratory (EMBL), Heidelberg, Germany. [28]School of Molecular Sciences and The Biodesign Institute, Arizona State University, Tempe, AZ, USA. [29]Department of Biochemistry, University of Zurich, Zurich, Switzerland. [30]Department of Chemistry, Ludwig-Maximilians-Universität München, München, Germany. [31]Institute of Physical & Theoretical Chemistry, Braunschweig Integrated Centre of Systems Biology (BRICS), and Laboratory for Emerging Nanometrology (LENA), Braunschweig University of Technology, Braunschweig, Germany. [32]BIOSS Centre for Biological Signalling Studies, University of Freiburg, Freiburg im Breisgau, Germany. [33]These authors contributed equally: Björn Hellenkamp, Sonja Schmid. *e-mail: jens.michaelis@uni-ulm.de; cseidel@hhu.de; t.craggs@sheffield.ac.uk; thorsten.hugel@pc.uni-freiburg.de

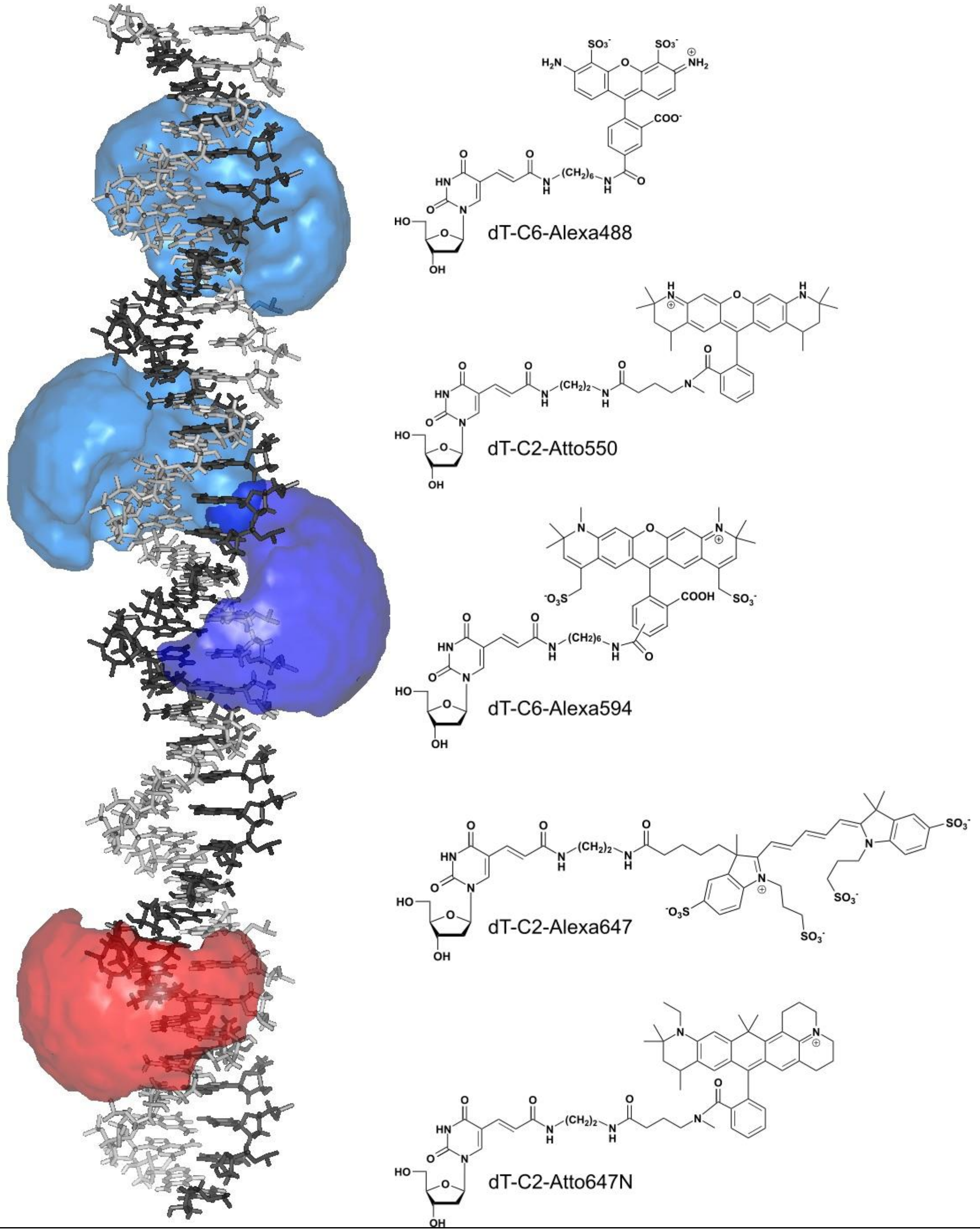

Supplementary Figure 1

DNA sample and utilized dyes.

Left: DNA model with dye accessible volumes of the donor (blue) and acceptor (red) that were used in this study, indicating lo-, mid- and hi-FRET samples. Right: Structural formula of the dyes used in this study. Based on dyes from Molecular Probes / Thermo Fisher Scientific (Waltham, USA) and Atto-tec (Siegen, D).

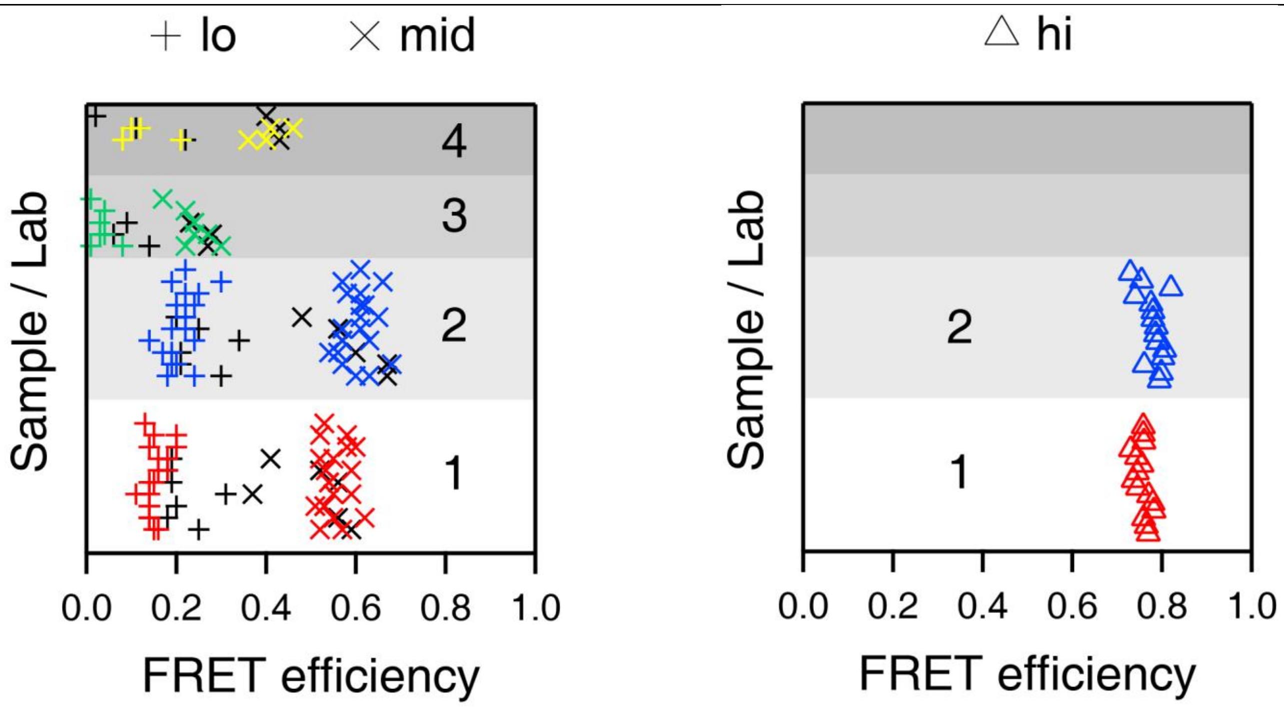

Supplementary Figure 2

FRET efficiencies of all labs for all measured samples as indicated.

FRET efficiencies of all labs for all measured samples as indicated. Sample 1 to 4 (see Supplementary Table 1 and Supplementary Note 1) are color coded (red, blue, green, yellow) for all data points from intensity-based techniques. For a table of $R_{\langle E \rangle}$ and R_{MP} and sample size for these measurements see Supplementary Table 4. Ensemble lifetime, single molecule lifetime and phasor approach derived data is shown in black. The FRET efficiencies (means and s.d.) for these measurements (depicted in black, sample size n) are: $E_{1a} = 0.21 \pm 0.05$ $(\mathrm{n} = 6)$; $E_{1b} = 0.51 \pm 0.08$ $(\mathrm{n} = 6)$; $E_{2a} = 0.25 \pm 0.06$ $(\mathrm{n} = 4)$; $E_{2b} = 0.59 \pm 0.07$ $(\mathrm{n} = 4)$; $E_{3a} = 0.10 \pm 0.04$ $(\mathrm{n} = 3)$; $E_{3b} = 0.26 \pm 0.03$ $(\mathrm{n} = 3)$; $E_{4a} = 0.12 \pm 0.10$ $(\mathrm{n} = 3)$; $E_{1a} = 0.42 \pm 0.02$ $(\mathrm{n} = 3)$. The left figure depicts all measurements from the main study, the right figure depicts all measurements from the later measurements of two additional samples (1-hi, 2-hi).

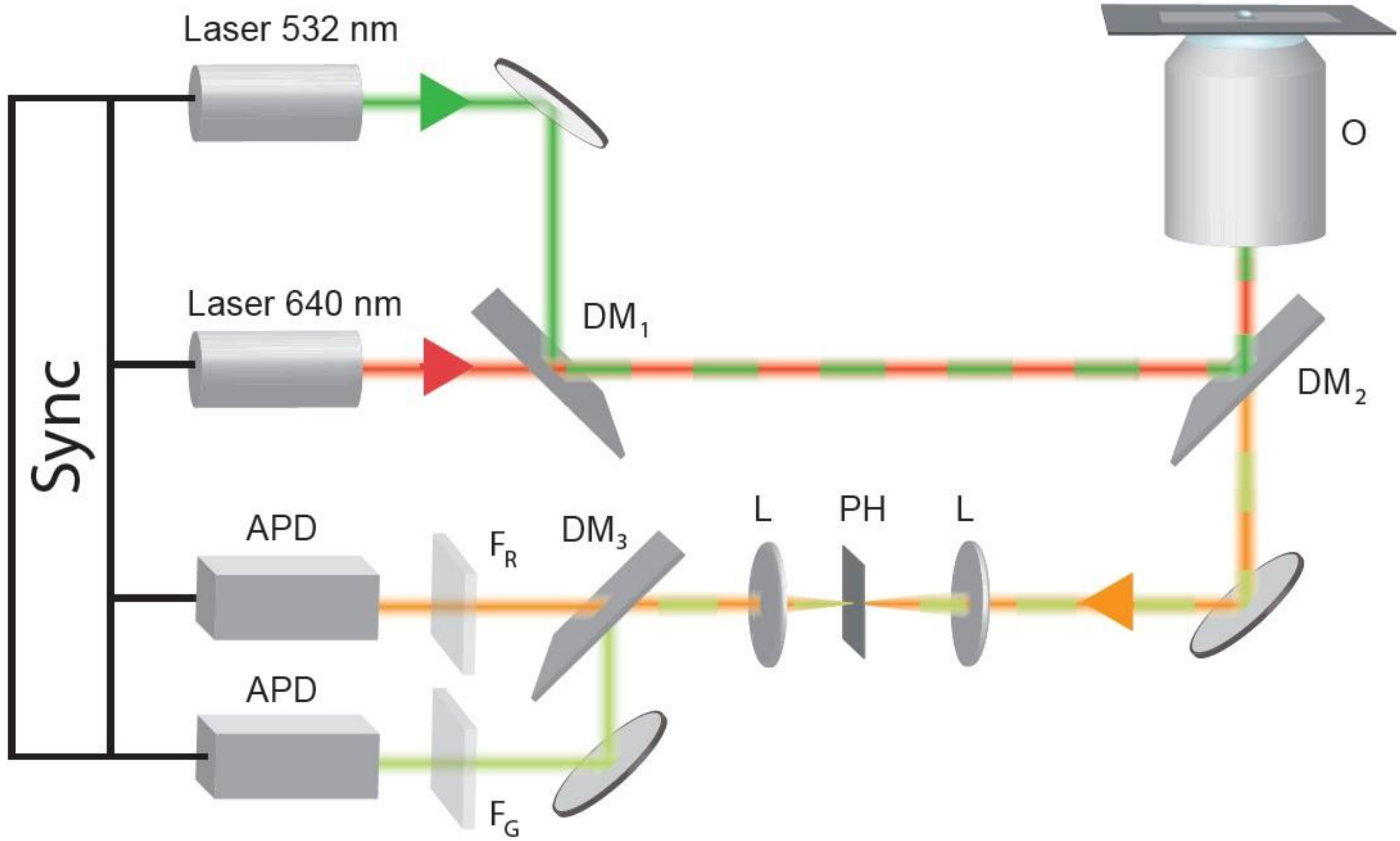

Supplementary Figure 3

Schematics of a typical confocal setup with alternating laser excitation / pulsed interleaved.

Schematics of a typical confocal setup with alternating laser excitation / pulsed interleaved excitation and color-sensitive detection. The most important elements are specified: Objective (O), dichroic mirror (DM), pinhole (P), spectral filter (F), avalanche photo diode (APD) and electronic micro- or picosecond synchronization of laser pulses and single photon counting (Sync).

Elements used for the correction factors in Table 2 (main text) were: F34-641 Laser clean-up filter z 640/10 (right after Laser 640 nm); DM_1: F43-537 laser beam splitter z 532 RDC ; DM_2: F53-534 Dual Line beam splitter z 532/633; DM_3: F33-647 laser- laser beam splitter 640 DCXR; F_G: F37-582 Brightline HC 582/75; F_R: F47-700 ET Bandpass 700/75; Objective: Cfi plan apo VC 60xWI, NA1.2; Detectors: MPD Picoquant (green), tau-SPAD, Picoquant (red); Pinholes: 100 µm; ; Laser power at sample: ≈ 100 µW; Beam diameter ≈ 2 mm; Diffusion time of Atto550 and Atto647N around 0.42 ms and 0.50 ms, respectively. For details on all used setups and analysis software, see Supplementary Note 8.

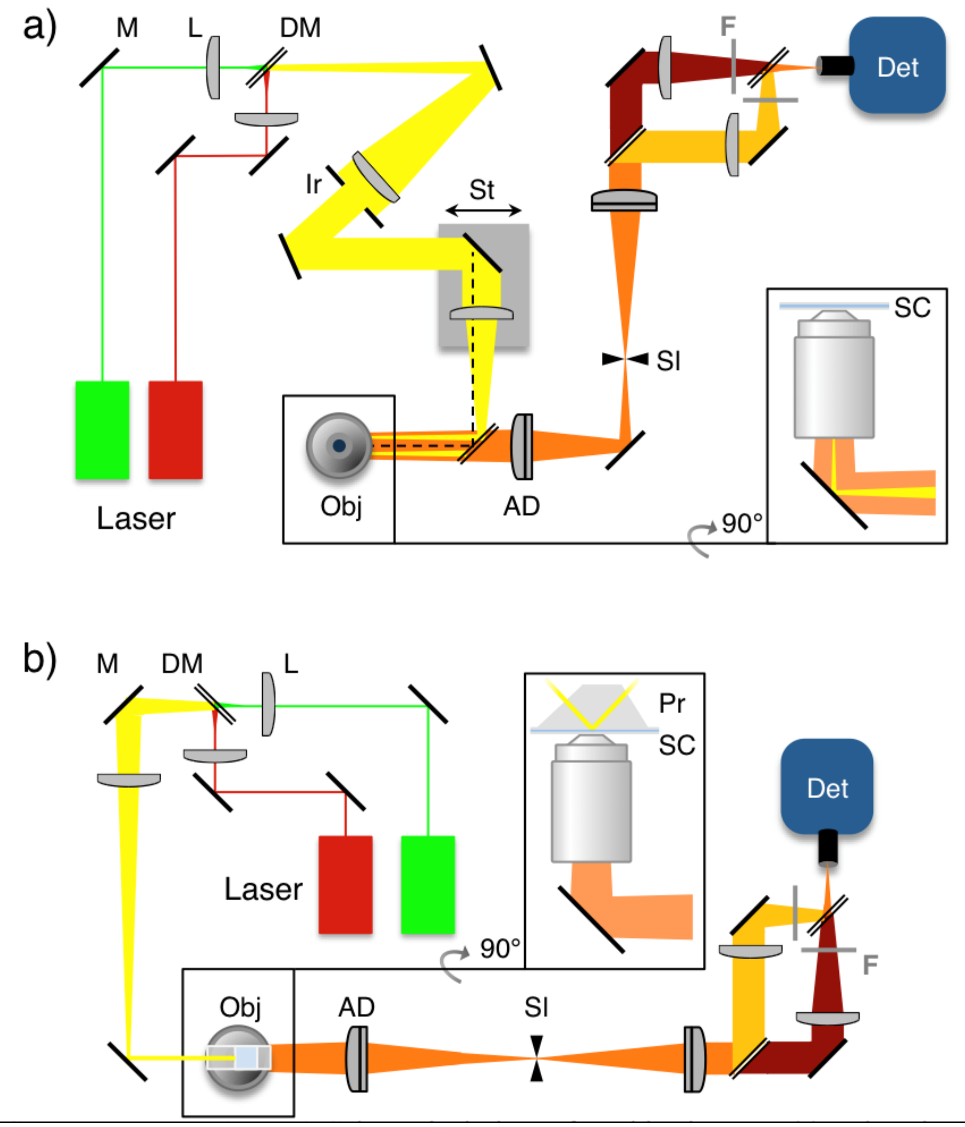

Supplementary Figure 4

Schematic designs of an objective-type (a) and a prism-type TIRF setup (b).

Green and red lasers are used to excite donor and acceptor dyes, respectively. M, mirror. L, lens. DM, dichroic mirror. Obj, objective. AD, achromatic doublet lens. Sl, tunable slit. F, filters. Det, detector (e.g. electron multiplying charge-coupled device camera, EMCCD). The inset shows a side view of the objective with the out-of-plane (45°) mirror below. SC, sample chamber. Ir, iris. St, translation stage, Pr, prism. The dashed black line in (a) indicates the *on-axis* path to the objective, in contrast to the displayed *off-axis* path for TIR illumination. Elements used for the correction factors in Table 2 (main text) were: Dichroic before objective: F53-534 (AHF), Dichroics in detection: F33-726 and F33-644 (AHF). Band pass filters in detection: BP F39-572 and BP F37-677 (AHF). SI: SP40 (Owis), Objective: CFI Apo TIRF 100x, NA 1.49 (Nikon). Camera: EMCCD, iXonUltra, Andor. Lasers: 532nm, Compass 215M (Coherent) and 635nm, Lasiris (Stoker Yale). Note that we have used a Dichroic in the fluorescence excitation and emission path that reflects the higher wavelength, but this does not have any effect on the FRET efficiency measurement and related determination of correction factors. For details on all used setups and analysis software, see Supplementary Note 8.

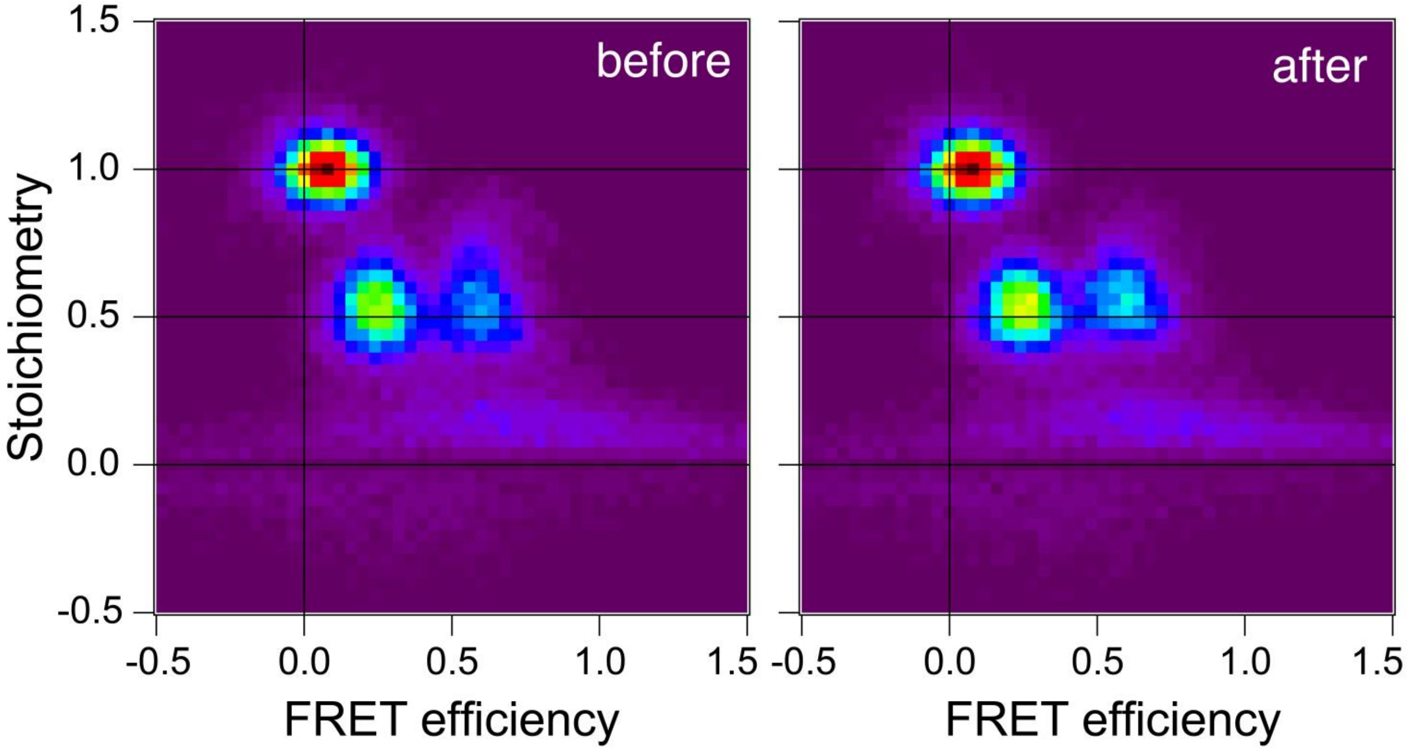

Supplementary Figure 5

Correcting for differences in the excitation intensity in TIRF microscopy.

Accounting for the differences in the excitation intensity profiles of the green and red laser across the field of view. The individual excitation profiles are determined as the mean image of a stack of images recorded while moving across a dense layer of dyes. In contrast to the uncorrected case ("before"), a position specific normalization creates narrower and more symmetric SE-populations ("after"). The standard corrections described in the main text are performed subsequently.

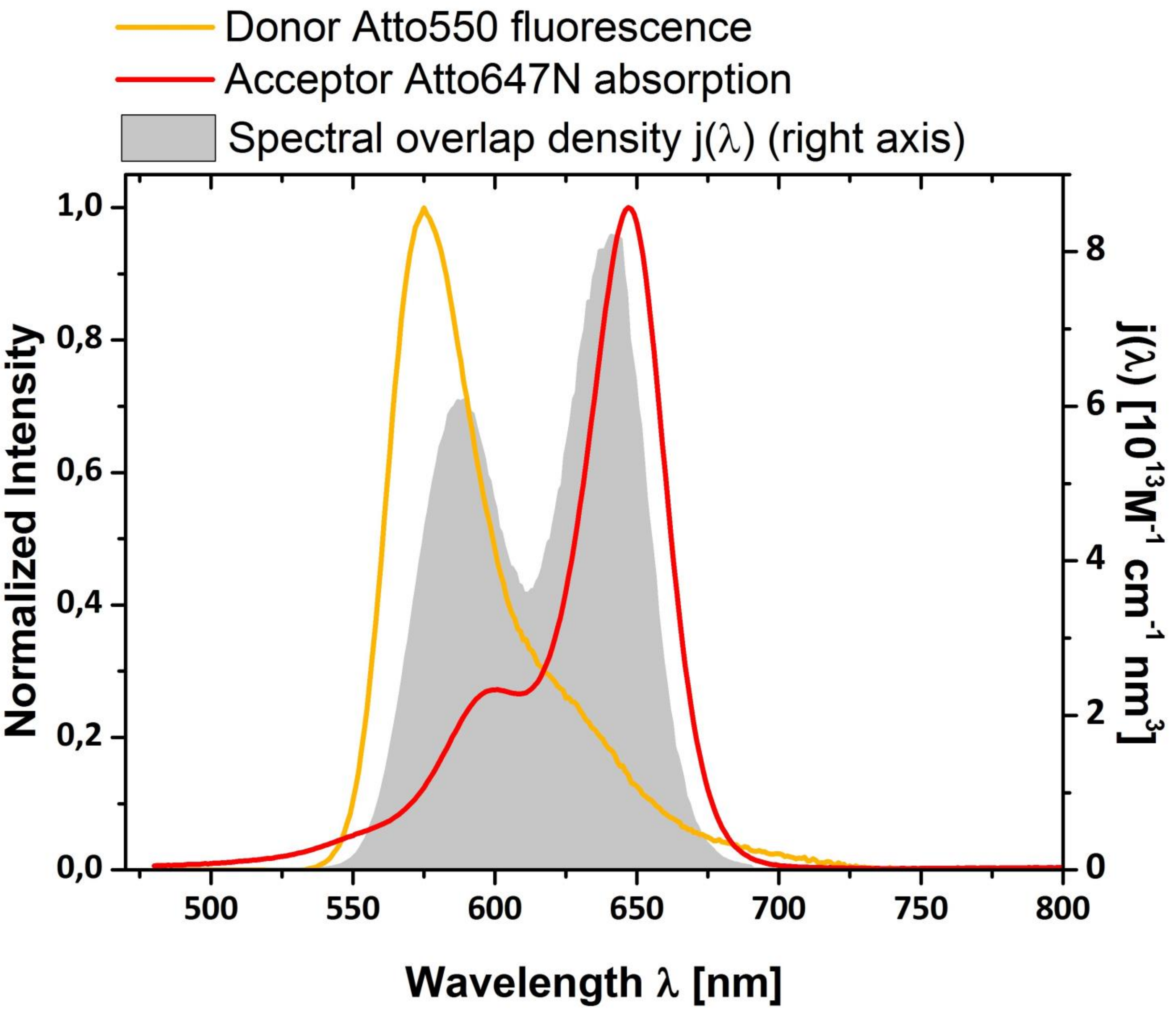

Supplementary Figure 6

Computation of the spectral overlap integral J

Computation of the spectral overlap integral J for the FRET pair Atto550-Atto647N in sample 1. Normalized donor fluorescence and acceptor absorption spectra normalized to the maximum (left scale). Spectral overlap density $j(\lambda)$ (right scale) to compute the spectral overlap integral J $[\mathrm{cm}^{-1}\mathrm{M}^{-1}\mathrm{nm}^{4}]$ with $J = \int_0^\infty \mathrm{j}(\lambda)\,\mathrm{d}\lambda$ and $j(\lambda) = \bar{F}_D(\lambda)\varepsilon_A(\lambda)\lambda^4$. The extinction coefficient ε_A of Atto647N was assumed to be $150000\ \mathrm{M}^{-1}\mathrm{cm}^{-1}$ at the maximum as provided by the manufacturer. The donor fluorescence and the acceptor absorption spectra were recorded in two laboratories in at least three independent experiments. Spectra with a flat baseline were selected. The computation was performed once.

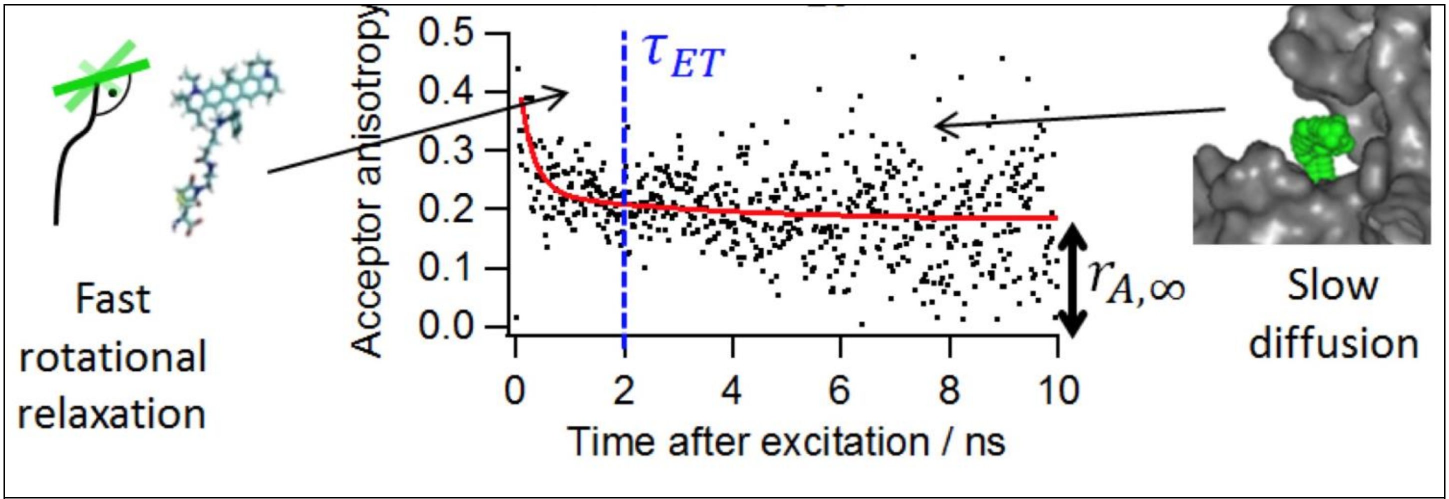

Supplementary Figure 7

Time-resolved anisotropies and FRET

The time-resolved anisotropies of dyes bound to a larger object (e.g. DNA or protein) normally consist of a fast decay from rotational relaxation of the dipole (left) and of a slow decay from translational relaxation (right). $\tau_{ET} = 1/k_{FRET}$: time of energy transfer; $r_{A,\infty}$: residual anisotropy of dye A. (Figure from ref. [1]). The data exemplarily shown is from a single measurement.

[1] *Hellenkamp, B., Wortmann, P., Kandzia, F., Zacharias, M. & Hugel, T. Multidomain Structure and Correlated Dynamics Determined by Self-Consistent FRET Networks. Nat. Meth. 14, 174-180 (2017).*

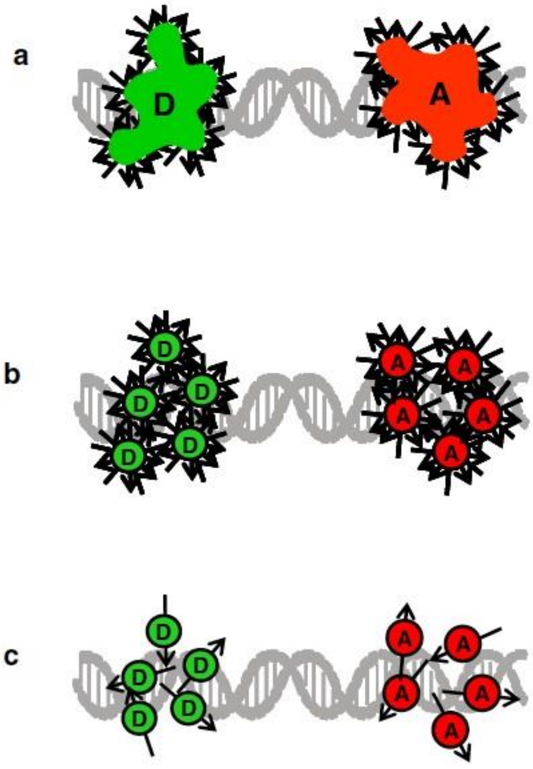

Supplementary Figure 8

Visualizations of different averages for efficiencies according to different fluorophore dynamics.

(a) Dynamic average, which applies in the case of the fluorophore movements being faster than the rate of energy transfer. There the rate of energy transfer has to be calculated taking into account the average over all possible distances and orientations. (b) Intermediate case, called the isotropic average, where the orientational variation of the fluorophores is faster than the rate of energy transfer while the positional variation is slower (c) Static case, where the fluorophore movements are much slower than the rate of energy transfer. In this case each distance and respective fluorophore orientation has to be taken into account with its individual transfer efficiency. These efficiencies then are averaged by the measurement process. (Figure from ref. [2]).

[2] *Wozniak, A. K., Schröder, G. F., Grubmüller, H., Seidel, C. A. M. & Oesterhelt, F. Single-Molecule FRET Measures Bends and Kinks in DNA. Proc. Natl. Acad. Sci. USA 105, 18337-18342 (2008).*

Supplementary Material:

Precision and accuracy of single-molecule FRET measurements – a worldwide benchmark study

Björn Hellenkamp[1a,°], Sonja Schmid[1a,°], Olga Doroshenko[20], Oleg Opanasyuk[20], Ralf Kühnemuth[20], Soheila Rezaei Adariani[15], Benjamin Ambrose[2], Mikayel Aznauryan[9], Anders Barth[21], Victoria Birkedal[9], Mark E. Bowen[11], Hongtao Chen[26], Thorben Cordes[14,25], Tobias Eilert[19], Carel Fijen[7], Christian Gebhardt[25], Markus Götz[1a], Giorgos Gouridis[14,25], Enrico Gratton[26], Taekjip Ha[22], Pengyu Hao[12], Christian A. Hanke[20], Andreas Hartmann[17], Jelle Hendrix[5,6], Lasse L. Hildebrandt[9], Verena Hirschfeld[16], Johannes Hohlbein[7,8], Boyang Hua[22], Christian G. Hübner[16], Eleni Kallis[19], Achillefs N. Kapanidis[10], Jae-Yeol Kim[23], Georg Krainer[17,18], Don C. Lamb[21], Nam Ki Lee[23], Edward A. Lemke[3a,b,c], Brié Levesque[11], Marcia Levitus[24], James J. McCann[11], Nikolaus Naredi-Rainer[21], Daniel Nettels[4], Thuy Ngo[22], Ruoyi Qiu[12], Nicole C. Robb[10], Carlheinz Röcker[19], Hugo Sanabria[15], Michael Schlierf[17], Tim Schröder[27], Benjamin Schuler[4], Henning Seidel[16], Lisa Streit[19], Johann Thurn[1a], Philip Tinnefeld[13,27], Swati Tyagi[3c], Niels Vandenberk[5], Andrés Manuel Vera[27], Keith R. Weninger[12], Bettina Wünsch[13], Inna S. Yanez-Orozco[15], Jens Michaelis[19,*], Claus A.M. Seidel[20,*], Timothy D. Craggs[2,10,*], Thorsten Hugel[1a,b,*]

Supplementary Information	Page
**Supplementary Table 1**<br>Samples	3
**Supplementary Table 2**<br>Spectroscopic parameters of the dyes in samples 1 and 2.	4
**Supplementary Table 3**<br>Typical correction factors	6
**Supplementary Table 4**<br>Resulting efficiencies and (relative) distances	6
**Supplementary Table 5**<br>Nomenclature and definitions	7
**Supplementary Table 6**<br>Typical dye parameters	8
**Supplementary Note 1:**<br>Further samples	9
**Supplementary Note 2**<br>Time-resolved experiments	10
**Supplementary Note 3**<br>AV simulations to compute donor acceptor distances	15
**Supplementary Note 4**<br>FRET efficiency measurements and distance determinations in more complex systems, e.g. proteins	16
**Supplementary Note 5**<br>$R_{\langle E \rangle}$ to R_{MP} conversion	17

**Supplementary Note 6**<br>Error propagation	20
**Supplementary Note 7**<br>MD simulations	21
**Supplementary Note 8**<br>Setup and data analysis details	25
**Bibliography**	35

Supplementary Table 1. The main focus in the manuscript are the 1 and 2 samples. The so-called donor strand (D-strand) is labeled with donor dye and acceptor strand (A-strand) with acceptor dye. The labeling sites of the donor and acceptor are shown in green and in red on the sequence respectively. See Supplementary Note 1 for further samples.

Name	Base position (Linker), strand	Dyes (Donor/Acceptor)	Sequence
**1-lo**	T 31(C2), D-strand<br>T 31(C2), A-strand	Atto550 NHS Ester/<br>Atto647N NHS	5′- GAG CTG AAA GTG TCG AGT TTG TTT GAG TGT TTG TCT GG - 3′<br>3′- CTC GAC TTT CAC AGC TCA AAC AAA CTC ACA AAC AGA CC - 5′ - biotin
**1-mid**	T 23(C2), D-strand<br>T 31(C2), A-strand	Atto550 NHS Ester/<br>Atto647N NHS	5′- GAG CTG AAA GTG TCG AGT TTG TTT GAG TGT TTG TCT GG - 3′<br>3′- CTC GAC TTT CAC AGC TCA AAC AAA CTC ACA AAC AGA CC - 5′ - biotin
**1-hi**	T 19(C2), D-strand<br>T 31(C2), A-strand	Atto550 NHS Ester/<br>Atto647N NHS	5′- GAG CTG AAA GTG TCG AGT TTG TTT GAG TGT TTG TCT GG - 3′<br>3′- CTC GAC TTT CAC AGC TCA AAC AAA CTC ACA AAC AGA CC - 5′ - biotin
**2-lo:**	T 31(C2), D-strand<br>T 31(C2), A-strand	Atto550 NHS Ester/<br>Alexa647 NHS Ester	5′- GAG CTG AAA GTG TCG AGT TTG TTT GAG TGT TTG TCT GG - 3′<br>3′- CTC GAC TTT CAC AGC TCA AAC AAA CTC ACA AAC AGA CC - 5′ - biotin
**2-mid:**	T 23(C2), D-strand<br>T 31(C2), A-strand	Atto550 NHS Ester/<br>Alexa647 NHS Ester	5′- GAG CTG AAA GTG TCG AGT TTG TTT GAG TGT TTG TCT GG - 3′<br>3′- CTC GAC TTT CAC AGC TCA AAC AAA CTC ACA AAC AGA CC - 5′ - biotin
**2-hi:**	T 19(C2), D-strand<br>T 31(C2), A-strand	Atto550 NHS Ester/<br>Alexa647 NHS Ester	5′- GAG CTG AAA GTG TCG AGT TTG TTT GAG TGT TTG TCT GG - 3′<br>3′- CTC GAC TTT CAC AGC TCA AAC AAA CTC ACA AAC AGA CC - 5′ - biotin

Supplementary Table 2: Spectroscopic parameters of the dyes in samples 1 and 2. Residual anisotropy r_∞, combined anisotropy r_c, fluorescence quantum yields of donor and acceptor $\Phi_{F,D}$ and $\Phi_{F,A}$, respectively (determined according to the procedure detailed in the online methods), and species average fluorescence lifetimes $\langle\tau\rangle_x$ for the samples 1 (Atto550 /Atto647N) and the samples 2 (Atto550 /Alexa647). All measurements were done in 20mM $MgCl_2$,5mM NaCl,5mM TRIS at pH 7.5 measurement buffer.

**Sample 1**	**1-lo (Atto550)**	**1-lo (Atto647N)**	**1-mid (Atto550)**	**1-mid (Atto647N)**	**1-hi (Atto550)**	**1-hi (Atto647N)**
Base position (Linker), strand	T 31,(C2), D-strand	T 31(C2), A-strand	T 23(C2), D-strand	T 31(C2), A-strand	T 19 (C2), D-strand	T 31(C2), A-strand
Residual anisotropy $r_{A,\infty}$ or $r_{D,\infty}$ [a]	0.08	0.07	0.11	0.07	0.13	0.07
Combined anisotropy r_c	0.07		0.09		0.10	
Steady state anisotropy r_S	0.11	0.09	0.13	0.09	0.15	0.09
Lifetime $\langle\tau\rangle_x$ / ns (SD: 2%) [b]	3.76	3.62	3.81	3.62	3.74	3.62
Fluorescence quantum yield $\Phi_{F,D}$ or $\Phi_{F,A}$	0.76±0.015 [c]	0.65 [d]	0.77±0.015 [c]	0.65 [d]	0.76±0.015 [c]	0.65 [d]
fraction bright a_b [g]	≈ 1 [e]	≈ 1 [e]	≈ 1 [e]	≈ 1 [e]	≈ 1 [e]	≈ 1 [e]
R_0 [Å]	62.6					
$\langle\Phi_{F,D}\rangle$	0.765±0.015					
ε_A [$M^{-1}cm^{-1}$]	150000					
J [cm^{-1} nm^4 M^{-1}]	$5.180\cdot10^{15}$					
n_{im}	1.40					
κ^2	2/3					
**Sample 2**	**2-lo (Atto550)**	**2-lo (Alexa647)**	**2-mid (Atto550)**	**2-mid (Alexa647)**	**2-hi (Atto550)**	**2-hi (Alexa647)**
Base position (Linker), strand	T 31, (C2), D-strand	T 31(C2), A-strand	T 23(C2), D-strand	T 31(C2), A-strand	T 19 (C2), D-strand	T 31(C2), A-strand
Residual anisotropy $r_{A,\infty}$ or $r_{D,,\infty}$ [a]	0.08	0.05	0.11	0.05	0.13	0.05
Combined anisotropy r_c	0.06		0.07		0.10	
Steady state anisotropy r_S	0.11	0.15	0.14	0.14	0.15	0.14
Lifetime $\langle\tau\rangle_x$ /ns (SD: 2%) [b]	3.76	1.19	3.81	1.19	3.74	1.19
Fluorescence quantum yield $\Phi_{F,D}$ or $\Phi_{F,A}$ [g]	0.77±0.015 [c]	0.39±0.015 [d]	0.77±0.015 [c]	0.39±0.015 [d]	0.77±0.015 [c]	0.39±0.015 [d]

fraction bright a_b [g]	≈ 1 [e]	0.75±0.02 [f]	≈ 1 [e]	0.85±0.02 [f]	≈ 1 [e]	0.86±0.02 [f]
R_0 [Å]	68.0					
$\langle \Phi_{F,D} \rangle$	0.765					
ε_A [$M^{-1}cm^{-1}$]	270000					
J [cm^{-1} nm^4 M^{-1}]	$8.502 \cdot 10^{15}$					
n_{im}	1.40					
κ^2	2/3					

[a] The depolarization time of all species are given together with their amplitudes in *Supplementary Table N2.2.*
[b] The fluorescence lifetimes of all species are given together with their species fractions in *Supplementary Table N2.1.*
[c] Measured relative to Rhodamine 6G in a steady state spectrometer in air-saturated Ethanol with $\Phi_{F,Rh6G}$= 0.95. In the same measurement we obtained for the free dye $\Phi_{F,Atto550}$ = 0.8±0.014 and $\tau_{Atto550}$= 3.60 ns ±2 % , which corresponds to the values given by the manufacturer.
[d] The following reference values for the free dyes in solution were used from the manufacturers to scale the fluorescence quantum via the fluorescence lifetime of the free dye:
$\Phi_{F,Atto647N}$ = 0.65; $\tau_{Atto647N}$= 3.5 ns; $\Phi_{F,Alexa647}$ = 0.33±0.015; $\tau_{Alexa647}$= 1.0 ns. The parameters of Alexa647 agree nicely with the values for free Cy5 $\Phi_{F,Cy5}$= 0.32±0.015 and $\Phi_{F,Cy5}$= 0.38±0.015 Cy5-labelled dsDNA [1]
A fluorescence lifetime analysis to relate the quantum yield Φ'_F and lifetime τ' specified by the manufacturer to the measured lifetime τ and quantum yield Φ_F:

$$\Phi_F = \Phi'_F \cdot \tau/\tau'$$

Here, we assume that the manufacturer Φ'_F is correct, that the radiative constant is unchanged and that the lifetime decay is monoexponential. For many dyes in distinct environments, this might not be the case.
[e] The excitation irradiance is usually low enough (especially in TIRF experiments) to avoid the population of dark states (triplet and radical states). The fraction of bright species a_b can be determined by fluorescence correlation spectroscopy.
[f] Cyanine dyes exhibit saturation effects due to cis-trans isomerization [2]. Moreover, Widengren et al [3] have shown that that the fraction of bright trans state a_b depends slightly on the FRET efficiency. In this study, we determined a_b by fluorescence correlation analysis of the FRET-sensitized acceptor signal in a confocal setup.
[g] Note that the correction factor γ was experimentally determined in this work. For completeness, we want to point out that the definition of γ in Online Methods section 1 can be used to compute the ratio of the detection efficiencies $g_{R|A}/g_{G|D}$ to check the detection performance of the setup provided the effective fluorescence quantum yields ${}^{eff}\Phi_F$ are known. Therefore we list the steady fluorescence quantum yields Φ_F and the fraction of bright species a_b.

Supplementary Table 3: Typical correction factors for sample 1 (Atto550-Atto647N) at given setups (reference lab). For the instrumental details of the setups see Supplementary Figures 3 and 4.

**Factor**	**Experiment type**	
	**confocal**	**TIRF**
α	0.11	0.07
β	1.80	0.85
γ	1.20	1.14
δ	0.11	0.065

Supplementary Table 4: Summary of resulting mean efficiencies $\langle E\rangle$, apparent distance $R_{\langle E\rangle}$, mean position distance R_{MP} and corresponding model distances $R_{\langle E\rangle}^{(model)}$ (Supplementary Note 3) and dynamic model distances $R_{\langle E\rangle}^{(dynamic\ model)}$ (Supplementary Note 7) and the experimental ratio $R_{rel} = R_{\langle E\rangle}^{(i)}/R_{\langle E\rangle}^{(mid)}$ and the model $R_{rel}^{(model)} = R_{\langle E\rangle}^{(model/i)} \ / \ R_{\langle E\rangle}^{(model,mid)}$ for all intensity based measurements. The errors (standard deviations) report on the precision of the measurements and not their accuracy. Literature values differ mainly because the refractive index of water is often assumed, while we used $n_{im} = 1.40$ here (see Online Methods, Section 4.1). Note that these errors only include the statistical variations of the FRET efficiencies, but do not include the error in the Förster radii, thus these errors represent the precision of the measurement, but not the accuracy. Including the knowledge of the dye attachment positions, a static structure of the DNA and this particular dye model, we computed also model values as described in Supplementary Note 3, which are also given here.

**Sample**	N	$\langle E\rangle$	R_0 **[Å]**	$R_{\langle E\rangle}$ **[Å]**	$R_{\langle E\rangle}^{(model)}$ **[Å]**	$R_{\langle E\rangle}^{(dynamic\ model)}$ **[Å]**	R_{rel}	$R_{rel}^{(model)}$	R_{MP} **[Å]**	$R_{MP}^{(model)}$ **[Å]**
1-lo	19	0.15±0.02		83.4±2.5	83.5±2.4	83.9	1.38	1.42	85.4±2.7	84.2±2.1
1-mid	19	0.56±0.03	62.6±4.0	60.3±1.3	58.7±1.6	60.3	1	1	58.2±1.7	55.8±2.3
1-hi	13	0.76±0.015		51.8±0.7	51.6±2.9	51.9	0.86	0.88	47.0±1.0	46.6 ±3.2
2-lo	19	0.21±0.04		85.4±3.4	83.9±2.2	84.2	1.34	1.41	86.9±3.7	84.2±2.4
2-mid	19	0.60±0.05	68.0±5.0	63.7±2.3	59.6±1.3	61.0	1	1	61.3±2.9	55.8±2.6
2-hi	13	0.78±0.025		55.0±1.3	52.3±1.9	52.6	0.86	0.88	50.1±1.8	46.6±1.8
3-lo	7	0.04±0.02	49.3[a]	89.5±12.3	82.4±2.4	83.1	1.49	1.46	85.7±5.3	84.0±2.1
3-mid	7	0.24±0.04		60.1±2.3	56.4±1.6	58.4	1	1	61.1±2.9	55.7±2.3
4-lo	4	0.13±0.06	57.0[a]	79.6±6.2	82.6±2.4	83.5	1.31	1.43	82.9±6.8	83.8±2.1
4-mid	4	0.41±0.04		60.7±1.7	57.6±1.6	59.5	1	1	60.4±2.3	55.5±2.3

[a] The R_0 for these samples have been taken from the literature and converted from a refractive index of n_{im} = 1.33 to n_{im} = 1.40:
Sample 3: R_0 = 49.3 Å from ref. [4]
Sample 4: R_0 = 57.0 Å from ref. [5]

Supplementary Table 5: Nomenclature and definitions. Since the nomenclature for FRET-based experiments is not consistent, we propose and use the following terms in this manuscript.

Central Definitions:		
$E = \frac{F_{A\|D}}{F_{D\|D} + F_{A\|D}}$	FRET efficiency	(1)
$S = \frac{F_{D\|D} + F_{A\|D}}{F_{D\|D} + F_{A\|D} + F_{A\|A}}$	Stoichiometry	(2)
$E = \frac{1}{1 + R_{DA}^6/R_0^6}$	FRET efficiency for a single donor acceptor distance R_{DA}	(3)
$\langle E\rangle = \frac{1}{nm}\sum_{\boldsymbol{i=1}}^{\boldsymbol{n}}\sum_{\boldsymbol{j=1}}^{\boldsymbol{m}}\frac{1}{1 + \left\|\boldsymbol{R}_{A(j)} - \boldsymbol{R}_{D(i)}\right\|^{\boldsymbol{6}}/R_0^6}$	Mean FRET efficiency for a discrete distribution of donor acceptor distances with the position vectors $\boldsymbol{R}_{D(i)}$ and $\boldsymbol{R}_{A(j)}$	(4)
$R_{\langle E\rangle} \equiv R(\langle E\rangle) = R_0(\langle E\rangle^{-1} - 1)^{1/6}$	The apparent donor acceptor distance is computed from the average FRET efficiency for a distance distribution. It is a FRET averaged quantity which was also referred to as FRET-averaged distance $\langle R_{DA}\rangle_{\mathrm{E}}$ (ref [1]).	(5)
$R_{MP} = \left\|\langle\boldsymbol{R}_{D(i)}\rangle - \langle\boldsymbol{R}_{A(j)}\rangle\right\| = \left\|\frac{1}{n}\sum_{i=1}^{n}\boldsymbol{R}_{D(i)} - \frac{1}{m}\sum_{j=1}^{m}\boldsymbol{R}_{A(j)}\right\|$	Distance between the mean dye positions with the position vectors $\langle\boldsymbol{R}_{D(i)}\rangle$ and $\langle\boldsymbol{R}_{A(j)}\rangle$	(6)
Subscripts:		
D or *A*	Concerning donor or acceptor	
A|D	Acceptor fluorescence given donor excitation, *D|D,A|A* accordingly	
Aem|Dex	Intensity in the acceptor channel given donor excitation, *Dem|Dex*, *Aem|Aex*, accordingly	
app	apparent, i.e. including systematic, experimental offsets	
Superscripts:		
BG	Background	
DO/ AO	Donor-only species/ Acceptor-only species	
DA	FRET species	
i -iii	Indicates (i) the uncorrected intensity; (ii) intensity after BG correction; (iii) intensity after BG, alpha and delta corrections	
Correction Factors:		
$\alpha = \frac{g_{R\|D}}{g_{G\|D}} = \frac{\langle\, {}^{ii}E_{app}^{(DO)}\rangle}{1 - \langle\, {}^{ii}E_{app}^{(DO)}\rangle}$	Leakage of D fluorescence into A channel	
$\beta = \frac{\sigma_{A\|R}}{\sigma_{D\|G}}\frac{I_{Aex}}{I_{Dex}}$	Normalization of excitation intensities, *I,* and cross-sections, σ, of A and D	
$\gamma = \frac{g_{R\|A}}{g_{G\|D}}\frac{{}^{eff}\Phi_{F,A}}{{}^{eff}\Phi_{F,D}}$	Normalization of effective fluorescence quantum yields, ${}^{eff}\Phi_F = a_b \cdot \Phi_F$, and detection efficiencies, *g,* of A and D. a_b is the fraction of molecules in the bright state and Φ_F is the fluorescence quantum yield without photophysical (saturation) effects.	

$\delta = \frac{\sigma_{A\|G}}{\sigma_{A\|R}} \frac{I_{Dex}}{I_{Aex}} = \frac{\langle {}^{ii}S_{app}^{(AO)} \rangle}{1 - \langle {}^{ii}S_{app}^{(AO)} \rangle}$	Direct acceptor excitation by the donor excitation laser (lower wavelength)
Primary Quantities:	
I	Experimentally observed intensity
F	Corrected fluorescence intensity
τ	Fluorescence lifetime [ns]
$\Phi_{F,A}$ or $\Phi_{F,D}$	Fluorescence quantum yield of A and D, respectively
r	Fluorescence anisotropy
R	Inter-dye distance [Å]
$\frac{R_0}{\text{Å}} = 0.2108 \sqrt[6]{\left(\frac{\Phi_{F,D}\kappa^2}{n_{im}^4}\right)\frac{J}{M^{-1}cm^{-1}nm^4}}$	Förster radius [Å], for a given J in units below (7)
$\kappa^2 = (\cos\theta_{AD} - 3\cos\theta_D \ \cos\theta_A)^2$	Dipole orientation factor
$J = \int_0^\infty \overline{F}_D(\lambda)\, \varepsilon_A(\lambda)\lambda^4 d\lambda$	Spectral overlap integral [$cm^{-1}M^{-1}nm^4$] (see Supplementray Figure 6)
$\overline{F}_D(\lambda)$ with $\int_0^\infty \overline{F}_D(\lambda)\, d\lambda = 1$	Normalized spectral radiant intensity of the excited donor [nm^{-1}], defined as the derivative of the emission intensity F with respect to the wavelength.
$\varepsilon_A(\lambda)$	Extinction coefficient of A [M^{-1} cm^{-1}]
n_{im}	Refractive index of the medium in-between the dyes
$g_{R\|A}$ or $g_{G\|D}$	Detection efficiency of the red detector (R) if only acceptor was excited or green detector (G) if donor was excited. Analogous for others.
$\sigma_{A/G}$	Excitation cross-section for acceptor when excited with green laser. Analogous for the others.

Supplementary Table 6: Typical parameters for sample 1 and sample 2 that define R_0 (Seidel lab). For their determination see Online Methods section 4.

dye pairs	κ^2	n_{im}	$\Phi_{F,D}$	ε_A [$M^{-1}cm^{-1}$]	J [$cm^{-1}M^{-1}nm^4$]	R_0 [Å]
Atto550-Atto647N	2/3	1.40	0.765	150000	$5.180 \cdot 10^{15}$	62.6
Atto550-Alexa647	2/3	1.40	0.765	270000	$8.502 \cdot 10^{15}$	68.0

Supplementary Note 1: Further samples

Name	Base position (Linker), strand	Dyes (Donor/ Acceptor)	Sequence
3-lo	T 31(C6), D-strand<br>T 31(C2), A-strand	Alexa488 Tetrafluorophenyl ester/<br>Atto647N NHS	5'- GAG CTG AAA GTG TCG AGT TTG TTT GAG TGT **T**TG TCT GG-3'<br>3'- CTC GAC T**T**T CAC AGC TCA AAC AAA CTC ACA AAC AGA CC-5'<br>- biotin
3-mid:	T 23(C6), D-strand<br>T 31(C2), A-strand	Alexa488 Tetrafluorophenyl ester/<br>Atto647N NHS	5'- GAG CTG AAA GTG TCG AGT TTG T**T**T GAG TGT TTG TCT GG-3'<br>3'- CTC GAC T**T**T CAC AGC TCA AAC AAA CTC ACA AAC AGA CC-5'<br>-biotin
4-lo:	T 31(C6), D-strand<br>T 31(C6), A-strand	Alexa488 Tetrafluorophenyl ester/<br>Alexa594 NHS	5'- GAG CTG AAA GTG TCG AGT TTG TTT GAG TGT **T**TG TCT GG-3'<br>3'- CTC GAC T**T**T CAC AGC TCA AAC AAA CTC ACA AAC AGA CC-5'<br>- biotin
4-mid:	T 23(C6), D-strand<br>T 31(C6), A-strand	Alexa488 Tetrafluorophenyl Ester/<br>Alexa594 NHS	5'- GAG CTG AAA GTG TCG AGT TTG T**T**T GAG TGT TTG TCT GG-3'<br>3'- CTC GAC T**T**T CAC AGC TCA AAC AAA CTC ACA AAC AGA CC-5'<br>- biotin

Even for samples 3 and 4 the precision of the hi-samples, where all individual FRET efficiencies were in a sensitive range of the specific dye pairs, is very good (2 - 4 %). Moreover, the experimental and model values of the low- and hi-samples agree very well with each other (the deviations range between 2 and 10 %). This suggests that we do not have dye artifacts for all four FRET pairs. The results obtained for the different FRET pairs will be important in the future to judge key aspects of different fluorophore properties.

NHS: N-hydroxysuccimidylester (mixed isomers according to the manufacturer)
TFP: (tetrafluorophenyl) ester (pure isomer according to the manufacturer)

Supplementary Note 2: Time-resolved experiments

Global fit of the time-resolved polarized and magic angle fluorescence ensemble data

All polarization resolved fluorescence decay curves ($F_{VV}(t), F_{VH}(t)$) with the Polarizer / Analyzer settings (Vertical, V /Horizontal, H) of singly labeled molecules were studied in ensemble experiments in the Seidel lab by high-precision time correlated single-photon counting and were fitted jointly with corresponding magic angle (M) fluorescence decay $f_{VM}(t)$ =($f(t)$). To reduce the number of parameters in the fits we used the so called homogenous approximation [6]. We assumed that de-excitation and depolarization of dyes are independent, i.e. in each donor de-excitation state dyes are characterized by the same set of depolarization times. For this case we can write model functions for the decay of the excited state population $f(t)$ and the fluorescence anisotropy $r(t)$:

$$f_{VV}(t) = f(t)[1 + 2r(t)] \quad (2.1)$$
$$f_{VH}(t) = f(t)[1 - r(t)] \quad (2.2)$$

with $f(t) = \sum_i x_i \; e^{-t/\tau_i}$ and $r(t) = \sum_j b^{(j)} e^{-t/\rho_j}$

Here, τ is the fluorescence lifetime and ρ is the depolarization times. x_i is (with $\sum_i x^{(i)} = 1$) the species fraction of molecules having the lifetime τ_i and the factor b_j is fraction of molecules having the depolarization time ρ_j where the fundamental anisotropy r_0 is given by $\sum_j b^{(j)} = r_0$ and the residual anisotropy is given by $b^{(3)} = r_\infty$. A maximum of three species for i and j were necessary to obtain satisfactory fits judged by χ^2_r.

To fit real experimental decays IRF, background and amplitudes of the VV, VH signals are accounted as:

$$F_{VV}(t) = F_0 \cdot IRF_{VV}(t) \otimes f_{VV}(t) + B_{VV} \quad (2.3)$$
$$F_{VH}(t) = g_{VV/VH} F_0 \cdot IRF_{VH}(t) \otimes f_{VH}(t) + B_{VH} \quad (2.4)$$

Where $g_{VV/VH}$ is a correction factor for a polarization dependent detection efficiency, F_0 - amplitude scaling factor, $IRF_{VV}(t)$, $IRF_{VH}(t)$ - instrument response functions and B_{VV}, B_{VH} - background values. The "$\otimes$" sign designates circular convolution.

The fit results for fluorescent signal in parallel and perpendicular polarization planes with respect to the vertically polarized excitation light with their rotation correlation times and amplitudes for D-only and A-only labeled DNA are presented in Supplementary Tables N2.1 and N2.2. The measured data and fitted curves with their weighted residuals are presented in the first column of the Supplementary Figure N2.1. Typical magic angle fluorescence decays are shown in the right column of the Supplementary Figure N2.1.

As expected [1], the amplitude b_1 for the fast depolarization motion with ρ_1 is approximately a factor 2 larger for dyes where the transition dipole moment is more perpendicular to the linker (disc case: Alexa 488 and Alexa594) than for dyes with a more parallel the transition dipole moment (cone case: Atto550, Atto647N and Alexa647) (compare Supplementary Figure 1 and Supplementary Table N2.2). Note that some dyes (e.g. Alexa488 and Alexa594) depolarize especially fast, because they have a large fraction of the fastest depolarization time such that $k_{rot} >> k_{FRET}$ might be satisfied

for even higher FRET efficiencies. The depolarization of these dyes is best described by a disc model [2].

Supplementary Table N2.1: Fluorescence lifetimes τ_i and their amplitudes x_i for all studied Donor-only (DO) and Acceptor-only samples (AO). The quality of the fit was judged by χ^2_r. [a,b]

Base position (Linker), strand	Dye [c]	Sample (DO or AO)	τ_1 [ns] (x_1)	τ_2 [ns] (x_2)	τ_3 [ns] (x_3)	$\langle\tau\rangle_x$ [ns]	χ^2_r [d]
T 31(C2), D-strand	Atto550	1-lo (DO), 2-lo (DO)	0.74 (0.03)	3.47 (0.59)	4.44 (0.38)	3.76	1.03
T 23(C2), D-strand	Atto550	1-mid (DO), 2-mid (DO)	0.86 (0.03)	3.54 (0.63)	4.59 (0.34)	3.81	1.02
T 19(C2), D-strand	Atto550	1-hi (DO), 2-hi (DO)	0.50 (0.04)	3.02 (0.30)	4.31 (0.66)	3.74	1.05
T 31(C2), A-strand	Atto647N	1-(AO), 3-(AO)	0.24 (0.04)	3.40 (0.52)	4.19 (0.44)	3.62	1.03
T 31 (C2), A-strand	Alexa647	2-(AO)	0.66 (0.05)	1.18 (0.90)	1.90 (0.05)	1.19	1.00
T 31(C6), D-strand	Alexa488	3-lo (DO)	0.36 (0.03)	2.37 (0.05)	4.11 (0.92)	3.91	1.03
T 23(C6), D-strand	Alexa488	3-mid (DO)	0.37 (0.04)	2.70 (0.04)	4.13 (0.92)	3.92	1.08
T 31 (C6), A-strand	Alexa594	4-(AO)	0.31 (0.03)	3.76 (0.64)	4.55 (0.33)	3.91	0.99

[a] in 20mM $MgCl_2$, 5mM NaCl, 5mM TRIS at pH 7.5 measurement buffer.

[b] typical errors: average lifetime: τ: ± 0.02 ns. Three lifetime: shortest lifetime τ_1 ± 20% (with x_1~ 15%), τ_2 ± 10% (with x_2 ~ 25%), τ_3 ± 3% (with x_3 ~ 15%).

[c] Spectral settings:

Atto550 (fluor. max 574 nm): excitation wavelength 552 nm, emission wavelength 580 nm (bandpass 5.4 nm).

Atto647N (fluor. max 664 nm): excitation wavelength 635 nm, emission wavelength 665 nm (bandpass 9.2 nm).

Alexa647 (fluor. max 665 nm): excitation wavelength 635 nm, emission wavelength 665 nm (bandpass 8.1 nm).

Alexa488 (fluor. max 525 nm): excitation wavelength 485 nm, emission wavelength 520 nm (bandpass 9.2 nm).

Alexa594 (fluor. max 617 nm): excitation wavelength 590 nm, emission wavelength 617 nm (bandpass 8.1 nm).

Note that the fluorescence lifetime analysis exhibited signatures of solvent relaxation. Therefore, we use wide bandpasses.

[d] χ^2_r was computed from a non-linear least squares fit of the corresponding model function to TCSPC data. Thus, χ^2_r refers to a single data set.

Supplementary Table N2.2: Rotation correlation times ρ_i with correspondent amplitudes b_i for Donor-only (DO) ($r_0 = 0.38$) and Acceptor-only (AO) ($r_0 = 0.38$) samples. The 1σ confidence range for the longest correlation time is indicated in square brackets. The fit model is described by eqs 2.1-2.2 with the model functions eqs. 2.3-2.4. The quality of the fit was judged by χ^2_r.

Base position (Linker),strand	dye	Sample (DO or AO)	ρ_1 [ns] (b_1)	ρ_2 [ns] (b_2)	ρ_3 [ns] (1σ conf.) ($b_3 = r_\infty$) [b]	χ^2_r [c]
T 31(C2), D-strand	Atto550	1-lo (DO), 2-lo (DO)	0.63 (0.14)	3.08 (0.16)	174 [97-540] (0.08)	1.04
T 23(C2), D-strand	Atto550	1-mid (DO), 2-mid (DO)	0.58 (0.12)	2.76 (0.15)	63 [50-86] (0.11)	1.01
T 19(C2), D-strand	Atto550	1-hi (DO), 2-hi (DO)	0.47 (0.10)	2.49 (0.16)	29.99 (0.13)	1.03
T 31(C2), A-strand	Atto647N	1-(AO), 3-(AO)	0.41 (0.14)	2.05 (0.17)	46 [37-62] (0.07)	1.01
T 31 (C2), A-strand	Alexa647 [a]	2 -(AO)	0.32 (0.09)	1.14 (0.23)	1e5 [125-∞] (0.06)	0.99
T 31(C6), D-strand	Alexa488	3-lo (DO)	0.25 (0.22)	1.31 (0.12)	31 [24-42] (0.04)	1.03
T 23(C6), D-strand	Alexa488	3-mid (DO)	0.26 (0.22)	1.43 (0.12)	37 [31-46] (0.04)	1.04
T 31 (C6), D-strand	Alexa594	4-(AO)	0.46 (0.22)	1.88 (0.09)	77 [58-121] (0.07)	1.02

[a] Only VV, VH depolarization curves used for fitting in this case.

[b] Due to fluctuations in the G-factor determinations we have small systematic errors; i.e. $\rho_3 > 20$ ns and not the fitted value

[c] χ^2_r was computed from a non-linear least squares fit of the corresponding model function to. Thus, χ^2_r refers to a single data set.

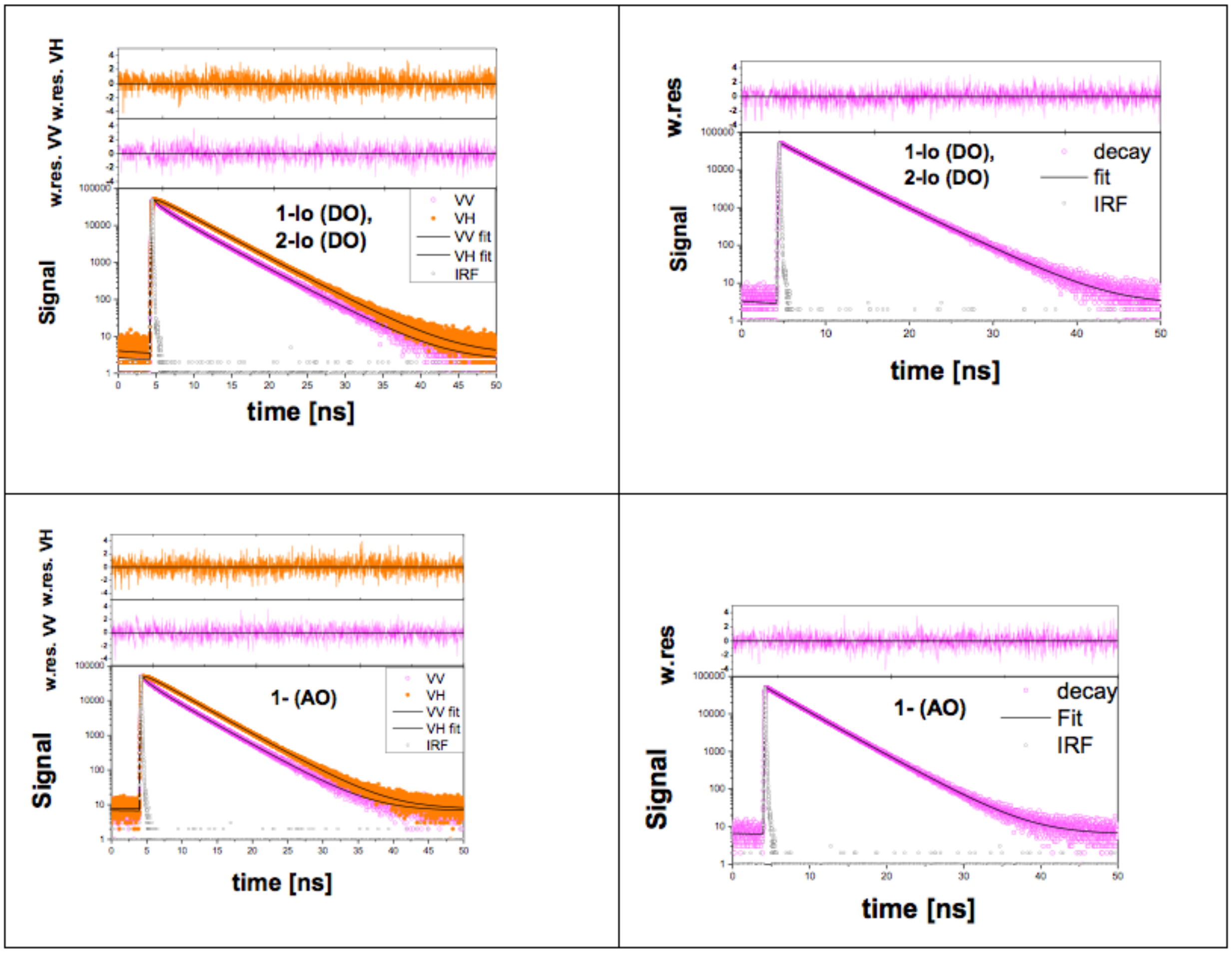

Supplementary Figure N2.1: Left pannels: Typical fluorescent signal in parallel and perpendicular polarization channels (magenta and orange) with the corresponding fits (black) for 1-lo (DO), 2-lo (DO), 1-(AO) samples with weighted residuals on the top. Right panels: the corresponding magic angle fluorescence decay curves with weighted residuals on the top. The fit results are displayed in Supplementary Tables N2.1 and N2.2 using fit procedure described by eqs 2.1-2.2 with the model functions eqs 2.3-2.4. The quality of the non-linear fit of the corresponding model function to TCSPC data was judged by χ^2_r. Thus, χ^2_r is a measure for the goodness of fit to a single data set.

Species average lifetime determination

Magic angle fluorescence decays were described with three fluorescence lifetimes τ_I and the species fractions x_i and thus species averaged lifetime $\langle\tau\rangle_x$ was calculated as:

$$\langle\tau\rangle_x = x_1\tau_1 + x_2\tau_2 + x_3\tau_3 \quad (2.5)$$

Experimental deviations obtained during lifetime-based experiments

The lifetime-based measurements had a significantly lower precision and accuracy than intensity-based measurements. The following factors might be responsible for the observed deviations in the fluorescence lifetime-based FRET experiments:

1. The precision propagates differently for intensity based and time-resolved techniques. In intensity-based FRET measurements the relative error of a normalized donor-acceptor

distance changes less with increasing distance. The precision of time-resolved FRET measurements is highest for small DA distances (the minimum is $R_{DA}/R_0 \approx 0.7$) which were not the focus of this study [7].

2. Time-resolved techniques strongly depend on a representative (chemically equivalent) Donor-only reference sample that is crucial for resolving large distances accurately.
3. If in the ensemble measurements the FRET sample contains also molecules, which are labeled with a donor, it becomes very difficult to resolve species with low FRET efficiencies.
4. The accuracy of time-resolved FRET measurements depends on an appropriate fit model. It is crucial to consider the heterogeneity of the donor lifetimes and dye-linker distributions (eq. 27 in ref. [6]). Moreover, the analysis model should allow fitting a variable fraction of donor-only species, which was needed for all provided samples. Altogether, this results in complex fit models which are not widely used in the FRET community because they are difficult to implement in commercial software. In contrast, some groups analyzed the donor decays by a simple series of exponentials, which results in a systematic shift of the obtained FRET parameters.

Thus, all four effects in time-resolved FRET measurements contributed to the fact that the precision and accuracy of the distances recovered especially for all lo-samples was markedly lower than that of intensity-based methods.

In contrast, the FRET efficiencies and inter-dye distances of the 1-hi and 2-hi samples were recovered very accurately by the Seidel lab as predicted by Ref [6] (Fig. 11), because the effects 1-3 do not apply anymore.

Supplementary Note 3: AV simulations to compute donor acceptor distances

The model for the double-stranded B-DNA is generated using the Nucleic Acid Builder version 04/17/2017 for Amber [8] (see Figure 1, main text). For modelling the dye molecule, we use a geometrical approach that considers sterically allowed dye positions within the linker length from the attachment point with equal probability. This defines the accessible volume (AV)[9]. The dye molecules are modeled as ellipsoids (approximated by three radii; AV3-Model) and AVs are generated using the FPS software[10]. For the distance computation a dye pair specific Förster Radius is used; i.e., it is assumed that within the AV the dye molecule samples all positions isotropically, however, for a single excitation it is at a fixed position. Moreover, it is assumed that dye rotation is so fast, that all possible orientations are sampled during the fluorescence lifetime and thus the factor $\langle\kappa^2\rangle = 2/3$ (isotropic coupling).

The boundary tolerance (called 'allowed sphere' in the FPS software) is used to ignore small residues that are fixed in the PDB-model, but flexible in solution. The larger this value, the larger the structural parts that are ignored for the AV generation. The labelling position is the C7 of the thymine (the C-atom of the thymine's methyl group). All mean geometric dyes parameters are estimated with ChemDraw software (see Supplementary Table N3.1). Further used parameters are: Boundary tolerance 0.5, accessible volume grid (rel.) 0.2; Min. grid [Å] 0.4, Search nodes: 3 and E samples: 200.

Supplementary Table N3.1: Recommended dye parameters for the AV simulations with AV3-model.

	**linker length [Å]**	**linker width [Å]**	**R1 [Å]**	**R2 [Å]**	**R3 [Å]**
dT-C6-Alexa488	20.5	4.5	5.0	4.5	1.5
dT-C2-Atto550	20.4	4.5	7.1	5.0	1.5
dT-C6-Alexa594	20.0	4.5	8.1	3.2	2.6
dT-C2-Alexa647	21.0	4.5	11.0	4.7	1.5
dt-C2-Atto647N	20.4	4.5	7.2	4.5	1.5

Error estimation. For each sample the distances between mean dye positions (R_{MP}^{model}) and expected experimentally observed apparent distance $R_{\langle E\rangle}^{model}$ are calculated (see Supplementary *Table 4*). The error for the model distances is estimated by varying the linker lengths (from 10 to 21 Å), linker width (from 4.0 to 5.0 Å), the dye model (single sphere with the radius 6 Å (AV1 model) and ellipsoid with three radii R1=7.1 Å, R2=4.5 Å and R3=1.8 Å (AV3 model) and the boundary tolerance between dye and DNA (0.5 and 1.5 Å). The standard deviation of all DA distances computed by FPS was used as error.

Supplementary Note 4: FRET efficiency measurements and distance determinations in more complex systems, e.g. proteins.

The described determination of FRET efficiencies and their transformation into distances is fully generalizable to more complex systems like protein samples. However, uncertainties can arise from insufficient dynamic averaging of dye position and orientation, which might be caused by static or dynamic site specific dye quenching as well as transient interactions between the dye molecule and its local environment. This can (and should) be tested for each dye pair by measuring the time-resolved anisotropies of donor-acceptor labelled samples. As a result of such tests, dye pairs with insufficient dynamic averaging (a combined anisotropy of donor and acceptor > 0.2) can either be removed from the analysis[11] or described using different dye models[12-14] (see also Online Methods). If this is done, the described error analysis is also fully transferable to protein systems. Note that the determined distance uncertainties (Fig. 5 and Online Methods) already include an estimated error for insufficient dynamic averaging.

Significant challenges arise from the need to label proteins with both donor and acceptor dyes. In systems where *intermolecular* distances are required, e.g. between different polypeptides (exchangeable homo dimers or hetero dimers), or between a protein and its bound DNA substrate, a single unique site on the protein for dye attachment is sufficient. In the case of homo dimers, the samples with two donors or two acceptors (about 25 % each) that will remain after the exchange, can be selected out following the ALEX procedure described in the main text. A single unique reaction site is often achieved using a unique reactive cysteine residue coupling to a maleimide-derivative of the chosen dye. However, this requires that other native, reactive cysteines are mutated (often to serine) and that the resulting 'cys-lite' protein remains active. For proteins with native reactive cysteines that cannot be removed by mutagenesis, incorporation of an unnatural amino acid carrying a completely orthogonal chemistry for dye attachment, or incorporation of specific peptide tags that can be site specifically labelled using enzymatic dye transfer reactions[15] are possible strategies.

Intramolecular FRET measurements can be more challenging, given the need to put both the donor and acceptor on the same molecule. The stochastic labelling of double cysteine mutants, leads to at least four labelled populations (AD, DA, DD, AA). A key strength of the presented ALEX method is the ability to separate the FRET species (DA, AD) from donor-only (DD) or acceptor-only (AA) labelled species. The difference between mixed populations of donors attached at two different positions (i.e. DA vs AD) can be an issue for the width of the measured FRET distribution but becomes smaller with increasing linker lengths and presumably depends on the spatial separation of the dyes. For linkers comparable to the ones used in this study the standard deviation was previously determined to be ~0.8 Å (Ref[6], Fig. 13). For some systems the differential reactivity of the two cysteines can be exploited to enable a biased labelling of the system[16]. Even a moderate (threefold) difference in local reactivity can yield highly specific double-labeling with sequential addition of the maleimide-dye derivatives. Alternatively, a combination with unnatural amino acids with mutually orthogonal reactivities can be incorporated[17] (for a review see ref[18]).

Future work will involve a comparative blind study using protein samples. This will be an even larger study and the next step towards having FRET-based structures in the PDB. Yet it is important to note that the current study is essential as the pre-requisite to a future protein study, as it e.g. presents all the procedures unified across the field for the first time.

Supplementary Note 5: $R_{\langle E\rangle}$ to R_{MP} conversion

For the conversion between $R_{\langle E\rangle}$ and R_{MP} we distinguish two cases, a known and unknown environment of the dye molecule:
Case 1, the local environment of the dye molecule is known. Here, we use average (apparent) distances from different data-sources, i.e., $R_{\langle E\rangle}$ from experiment, and R_{MP} from coarse-grained-structural modeling to generate conversion functions. Typically, we use coarse-grained simulations to approximate accessible volumes (AVs) for biomolecules [10]. These AVs are translated / rotated and the average apparent DA distance ($R_{\langle E\rangle}$ and the distance R_{MP} are calculated, which introduces noise. The resulting conversion tables are approximated by third order polynomials. Here we used the AVs for the samples 1,2,3,4 low and high FRET correspondingly. For the polynomial $R_{MP} = (a_0 + a_1 R_{\langle E\rangle} + a_2 R_{\langle E\rangle}^2 + a_3 R_{\langle E\rangle}^3)$ the coefficients are given in the Supplementary Table N5.1 for all FRET pairs.
Note that the conversion functions are specific for the chosen dye pair because they depend on the Förster Radius of the FRET pair and the used dye parameters for the AV simulation. The differences between AVs in different molecular environments (DNA or protein) become smaller the less restricted the dye is. The offset of the conversion function depends on the size of the dye spheres and the linker lengths.

Supplementary Table N5.1. Conversion polynomial for $R_{MP} = (a_0 + a_1 R_{\langle E\rangle} + a_2 R_{\langle E\rangle}^2 + a_3 R_{\langle E\rangle}^3)$ using the dye pair specific R_0 and specific AVs of the samples 1, 2, 3 and 4, respectively. Note that these polynomials are only valid for this specific geometry and dyes.

FRET pair	R_0 [Å]	a_0 [Å]	a_1	a_2 [Å]$^{-1}$	a_3 [Å]$^{-2}$
**Atto550-Atto647N**	62.6	-41.8	2.13	$-0.92\cdot10^{-2}$	$2.40\cdot10^{-5}$
**Atto550-Alexa647**	68.0	-40.3	1.99	$-0.74\cdot10^{-2}$	$1.67\cdot10^{-5}$
**Alexa488-Atto647N**	49.3	-53.8	2.83	$-1.89\cdot10^{-2}$	$6.17\cdot10^{-5}$
**Alexa488-Alexa594**	57.0	-43.9	2.24	$-1.02\cdot10^{-2}$	$2.48\cdot10^{-5}$

Case 2, the local environment of the dye molecule is not known. Here, we make the most general assumption that the AV can be approximated by a sphere with its radius estimated from the size of the dye and linker length (slightly smaller than the dye and linker length). This allows us to estimate the conversion of $R_{\langle E\rangle}$ to R_{MP} by performing Monte Carlo simulations[19]. In the Monte Carlo simulation, we place 10,000 positions uniformly distributed within a sphere of 18 Å radius for both dyes. For a given distance of the mean position of these spheres (R_{MP}) we calculate the respective FRET efficiencies via the Förster formula with an orientation factor of $\kappa^2 = 2/3$. The mean of these values is an unbiased estimate for the expected value of the FRET efficiency. We vary the mean position distance from 0.5 R_0 to 1.5 R_0 and fit the resulting means with a third order polynomial (coefficients a_0, a_1, a_2 and a_3). For convenience the conversion coefficients for a large range of used Förster radii were determined and given here in Supplementary Table N5.2.
The equivalence of both approaches is demonstrated in Supplementary Figure N5.1 for the dye pair Atto550-Atto647N.

Supplementary Table N5.2.: Coefficients for the conversion polynomials $R_{\langle E\rangle}$ to R_{MP} for case 2. These are valid for the specified Förster radius R_0.

R_0 [Å]	a_0 [Å]	a_1	a_2[Å]$^{-1}$	a_3[Å]$^{-2}$
50	-33.6	1.65	$3.25\cdot10^{-3}$	$-7.26\cdot10^{-5}$
51	-34.0	1.70	$1.55\cdot10^{-3}$	$-5.68\cdot10^{-5}$
52	-33.0	1.64	$2.25\cdot10^{-3}$	$-5.85\cdot10^{-5}$
53	-31.7	1.57	$2.93\cdot10^{-3}$	$-5.91\cdot10^{-5}$
54	-32.8	1.64	$1.27\cdot10^{-3}$	$-4.65\cdot10^{-5}$
55	-32.0	1.61	$1.43\cdot10^{-3}$	$-4.46\cdot10^{-5}$
56	-30.0	1.51	$2.78\cdot10^{-3}$	$-4.98\cdot10^{-5}$
57	-30.0	1.51	$2.19\cdot10^{-3}$	$-4.39\cdot10^{-5}$
58	-30.0	1.52	$1.73\cdot10^{-3}$	$-3.91\cdot10^{-5}$
59	-27.7	1.42	$3.03\cdot10^{-3}$	$-4.37\cdot10^{-5}$
60	-29.1	1.49	$1.55\cdot10^{-3}$	$-3.42\cdot10^{-5}$
61	-27.5	1.42	$2.36\cdot10^{-3}$	$-3.67\cdot10^{-5}$
62	-26.5	1.37	$2.77\cdot10^{-3}$	$-3.74\cdot10^{-5}$
63	-27.3	1.42	$1.67\cdot10^{-3}$	$-2.99\cdot10^{-5}$
64	-25.6	1.35	$2.56\cdot10^{-3}$	$-3.28\cdot10^{-5}$
65	-26.1	1.38	$1.88\cdot10^{-3}$	$-2.83\cdot10^{-5}$
66	-26.4	1.39	$1.42\cdot10^{-3}$	$-2.50\cdot10^{-5}$
67	-24.9	1.33	$2.07\cdot10^{-3}$	$-2.69\cdot10^{-5}$
68	-24.1	1.30	$2.27\cdot10^{-3}$	$-2.68\cdot10^{-5}$
69	-24.2	1.31	$1.90\cdot10^{-3}$	$-2.39\cdot10^{-5}$
70	-23.3	1.28	$2.29\cdot10^{-3}$	$-2.49\cdot10^{-5}$
71	-24.4	1.33	$1.30\cdot10^{-3}$	$-1.95\cdot10^{-5}$
72	-23.4	1.29	$1.80\cdot10^{-3}$	$-2.11\cdot10^{-5}$
73	-22.6	1.27	$1.86\cdot10^{-3}$	$-2.04\cdot10^{-5}$
74	-23.0	1.28	$1.53\cdot10^{-3}$	$-1.84\cdot10^{-5}$
75	-22.4	1.26	$1.62\cdot10^{-3}$	$-1.80\cdot10^{-5}$
76	-21.8	1.24	$1.70\cdot10^{-3}$	$-1.77\cdot10^{-5}$
77	-21.6	1.24	$1.58\cdot10^{-3}$	$-1.66\cdot10^{-5}$
78	-21.8	1.25	$1.28\cdot10^{-3}$	$-1.47\cdot10^{-5}$
79	-21.2	1.23	$1.44\cdot10^{-3}$	$-1.49\cdot10^{-5}$
80	-21.2	1.24	$1.32\cdot10^{-3}$	$-1.41\cdot10^{-5}$
81	-20.5	1.22	$1.43\cdot10^{-3}$	$-1.39\cdot10^{-5}$
82	-20.3	1.21	$1.40\cdot10^{-3}$	$-1.34\cdot10^{-5}$
83	-20.5	1.22	$1.17\cdot10^{-3}$	$-1.20\cdot10^{-5}$
84	-19.8	1.20	$1.36\cdot10^{-3}$	$-1.24\cdot10^{-5}$
85	-20.3	1.22	$0.96\cdot10^{-3}$	$-1.05\cdot10^{-5}$
86	-19.5	1.19	$1.20\cdot10^{-3}$	$-1.10\cdot10^{-5}$
87	-19.1	1.18	$1.22\cdot10^{-3}$	$-1.08\cdot10^{-5}$
88	-19.0	1.18	$1.15\cdot10^{-3}$	$-1.02\cdot10^{-5}$
89	-19.0	1.18	$1.04\cdot10^{-3}$	$-0.95\cdot10^{-5}$
90	-18.8	1.18	$0.99\cdot10^{-3}$	$-0.91\cdot10^{-5}$

We tested that the polynomials derived using case 1 or case 2 yield almost identical conversion functions (Supplementary Figure N5.1).

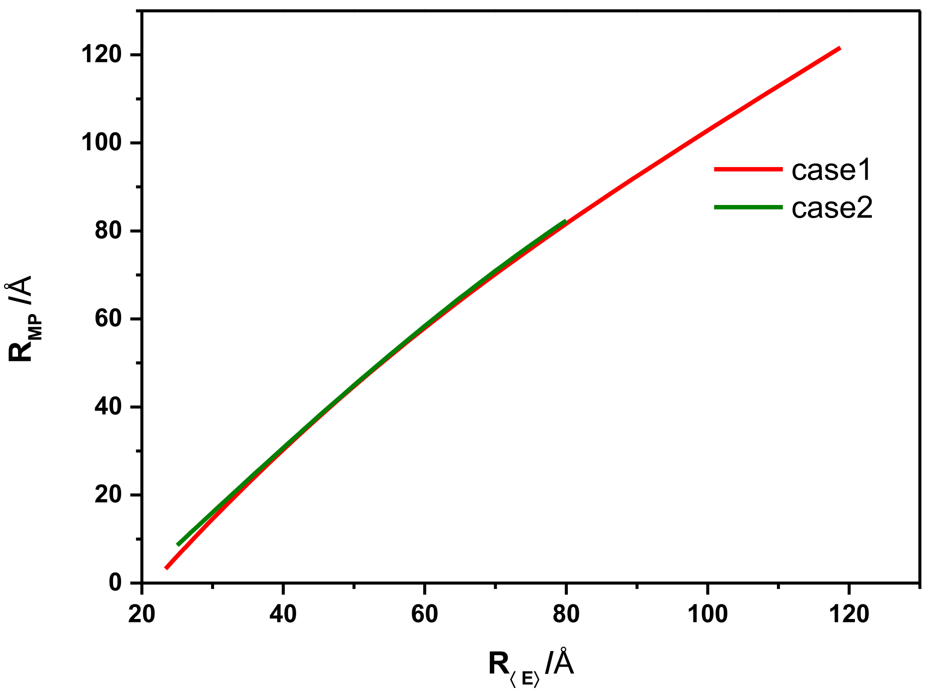

Supplementary Figure N5.1. Polynomial $R_{MP} = a_0 + a_1 R_{\langle E\rangle} + a_2 R_{\langle E\rangle}^2 + a_3 R_{\langle E\rangle}^3$ for the dye pair Atto550-Atto647N with AVs for DNA (case 1: $R_0 = 62.6$Å) and with the approximation by a sphere (case 2: $R_0 = 62.0$ Å). Interpolated points in steps of 1 Å.

Supplementary Note 6: Error propagation

Based on the measurements of sample 1-lo and 1-hi, we performed an error propagation using ΔE = 0.033 (which was the precision for these two best investigated samples). *Figure 5* follows from the following distance uncertainty:

$$\Delta R(R_0, \Delta R_0, \Delta E|R) = \sqrt{\left(\frac{\partial R(R_0,E)}{\partial R_0}\cdot \Delta R_0\right)^2 + \left(\frac{\partial R(R_0,E)}{\partial E}\cdot \Delta E\right)^2} = \sqrt{\left(\frac{R}{R_0}\cdot \Delta R_0\right)^2 + \left(\frac{1}{6}\left(1+\left(\frac{R}{R_0}\right)^6\right)^2\left(\frac{R}{R_0}\right)^{-5} R_0\cdot \Delta E\right)^2} \quad (6.1)$$

In the following, we performed more detailed error propagation with disentangled error sources. We estimate the uncertainties of all quantities separately and propagate them towards an uncertainty in the distance. The overall uncertainty in the distance is given by:

$$\Delta R\left(R_0, \Delta R_0, \gamma, \Delta\gamma, \langle F\rangle, \Delta I^{(BG)}_{Dem|Dex}, \Delta I^{(BG)}_{Aem|Dex}, \Delta\beta, \Delta\alpha \middle| R\right) = \sqrt{\Delta R_{R0}^2 + \Delta R_\gamma^2 + \Delta R_{bgD}^2 + \Delta R_{bgA}^2 + \Delta R_\alpha^2 + \Delta R_\delta^2}$$

(6.2)

with the following error contribution for the Förster radius:

$$\Delta R_{R0}(R) = R\frac{\Delta R_0}{R_0} \quad (6.3)$$

And the following error contribution for the gamma factor:

$$\Delta R_\gamma(R) = \frac{R}{6}\cdot\frac{\Delta\gamma}{\gamma} \quad (6.4)$$

And the following error contribution for the background in the donor channel after donor excitation:

$$\Delta R_{bgD}(R) = \frac{R}{6}\left[\gamma\left(1+\left(\frac{R_0}{R}\right)^6\right) + \alpha\left(1+\left(\frac{R}{R_0}\right)^6\right)\right]\cdot\frac{\Delta I^{(BG)}_{Dem|Dex}}{\langle F\rangle} \quad (6.5)$$

And the following error contribution for the background in the acceptor channel after donor:

$$\Delta R_{bgA}(r) = -\frac{R}{6}\left(1+\left(\frac{R}{R_0}\right)^6\right)\cdot\frac{\Delta I^{(BG)}_{Aem|Dex}}{\langle F\rangle} \quad (6.6)$$

And the following error contribution for the direct excitation factor of the acceptor with the green laser:

$$\Delta R_\delta(R) = -\frac{R}{6}\beta\left(1+\left(\frac{R}{R_0}\right)^6\right)\cdot\Delta\delta \quad (6.7)$$

And the following error contribution for the leakage factor of donor fluorescence in the acceptor channel:

$$\Delta R_\alpha(R) = -\frac{R}{6}\frac{1}{\gamma}\left(\frac{R}{R_0}\right)^6\cdot\Delta\alpha \quad (6.8)$$

Please note that for determination of background we set $\gamma = \beta = 1$ and $\alpha = \delta = 0$.This represents the ideal values. Further parameters and uncertainties are taken from the reference lab: $\Delta\gamma/\gamma = 0.1$, $\langle F\rangle = 50$, $\Delta I^{(BG)}_{Dem|Dex}$=1, $\Delta I^{(BG)}_{Aem|Dex}$= 1, $\Delta\delta/\delta = 0.1$, $\Delta\alpha/\alpha = 0.1$, $\Delta R_0 = 0.07$. See Online Methods, Section 1 and 3 for the nomenclature and details on the Förster radius. $\langle F\rangle$ is the average sum of the corrected donor and acceptor fluorescence.

The above error analysis is based on R_{DA} and may be further propagated to the apparent donor-acceptor distance $R_{\langle E\rangle}$ and the distance between the mean positions of the dyes, R_{MP}, when the above model assumption of a freely rotating and diffusing dye is applied. This becomes very involved and does not show significant deviations from Figure 5 in the main text.

Supplementary Note 7: MD simulations

While the analysis in this paper used a static model for the double-stranded DNA structure, there is plenty of experimental and theoretical evidence that DNA is not completely rigid [20-23]. Therefore, we performed Molecular dynamics (MD) simulations of the DNA molecule to explore its rigidity using the latest force fields which were reported to be consistent with experimental observables of the conformational flexibility of dsDNA.

The all-atom MD simulations were performed with the Amber16 suite of programs [24] using the bsc1 force field [8]. The initial structure of the B-DNA molecule, which was generated by 3D-DART (see main text), was placed in an octahedral box of TIP3P water molecules [25], such that the distance between the edge of the water box and the closest DNA atom was at least 11 Å. $MgCl_2$ and NaCl were added to achieve concentrations of 20 mM and 10 mM, respectively. For Na^+ and Cl^-, the parameters by Joung and Cheatham [26] were used, while for Mg^{2+} the parameters by Li et al. [27] were used.

Each system was then prepared based on a protocol used earlier [28]. The simulation system was minimized by 200 steps of steepest descent and subsequently 50 steps of conjugate gradient minimization. The minimized system was heated from 100 to 300 K over 50 ps, and subsequently the solvent density was adjusted for 150 ps by NPT-MD simulation. During the previous two steps, harmonic force restraints were applied on all solute atoms with force constants of 5 kcal mol^{-1} $Å^{-2}$. These harmonic force restraints were gradually reduced to 1 kcal mol^{-1} $Å^{-2}$ during 250 ps of NVT-MD simulation. This step was followed by 50 ps of NVT-MD simulation without positional restraints. Subsequently, we performed five independent MD simulations of 250 ns length each. The time step for all MD simulations was 2 fs. Coordinates were extracted from the simulations every 20 ps. The traces of the RMSD as a function of calculation time show that the calculations have converged (Supplementary Figure N.7.1).

We used the FPS program [10] to calculate FRET efficiencies for the structural ensemble of the MD simulation, i.e. for each structure the AVs with the spatial dye (D and A) distributions is calculated and the average FRET efficiency is computed.

The ensembles from the MD simulations suggest that the DNA is not completely rigid, but exhibits some bending motion (Supplementary Figure N.7.2). The obtained distributions of FRET efficiencies show that the ensembles from the MD simulations yield comparable, but slightly lower mean FRET efficiencies and thus longer distances than for the static model (Supplementary Figure N.7.3, Supplementary Table N.7.1).

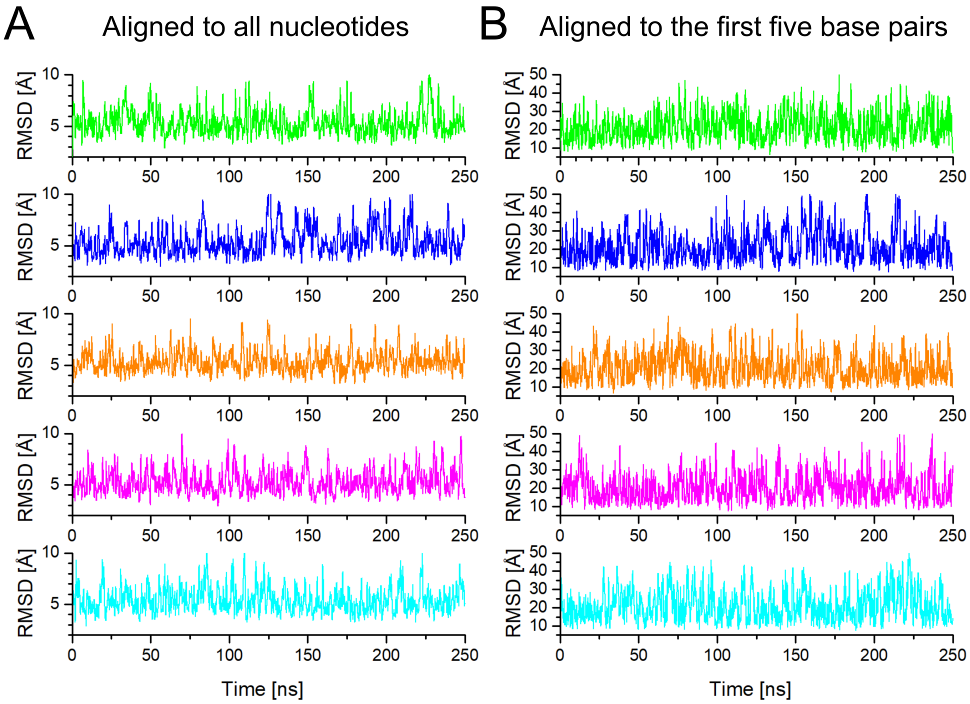

Supplementary Figure N.7.1: RMSD calculated over the simulation time for the five simulations performed. The RMSD was calculated with respect to the straight DNA molecules, which served as starting structure, considering all atoms for the calculation after aligning the structures to the straight DNA using all atoms (A) and only the first five base pairs (B). For better visibility, the lines were smoothed with a sliding average window of length 200 ps.

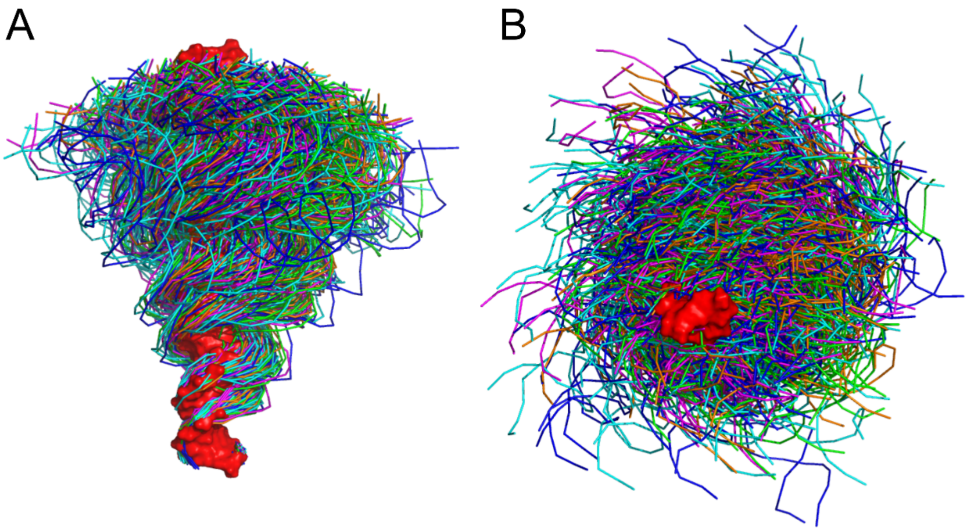

Supplementary Figure N.7.2: Structural ensembles from the MD simulations. The starting structure (red surface representation) was overlaid with conformations extracted from the MD simulations using the first 5 base pairs of the DNA. The five independent MD simulations are shown as differently colored ribbons (green, blue, orange, magenta, and cyan). For visibility, snapshots extracted every 2 ns were used for this representation. A: Side view; B: Top view.

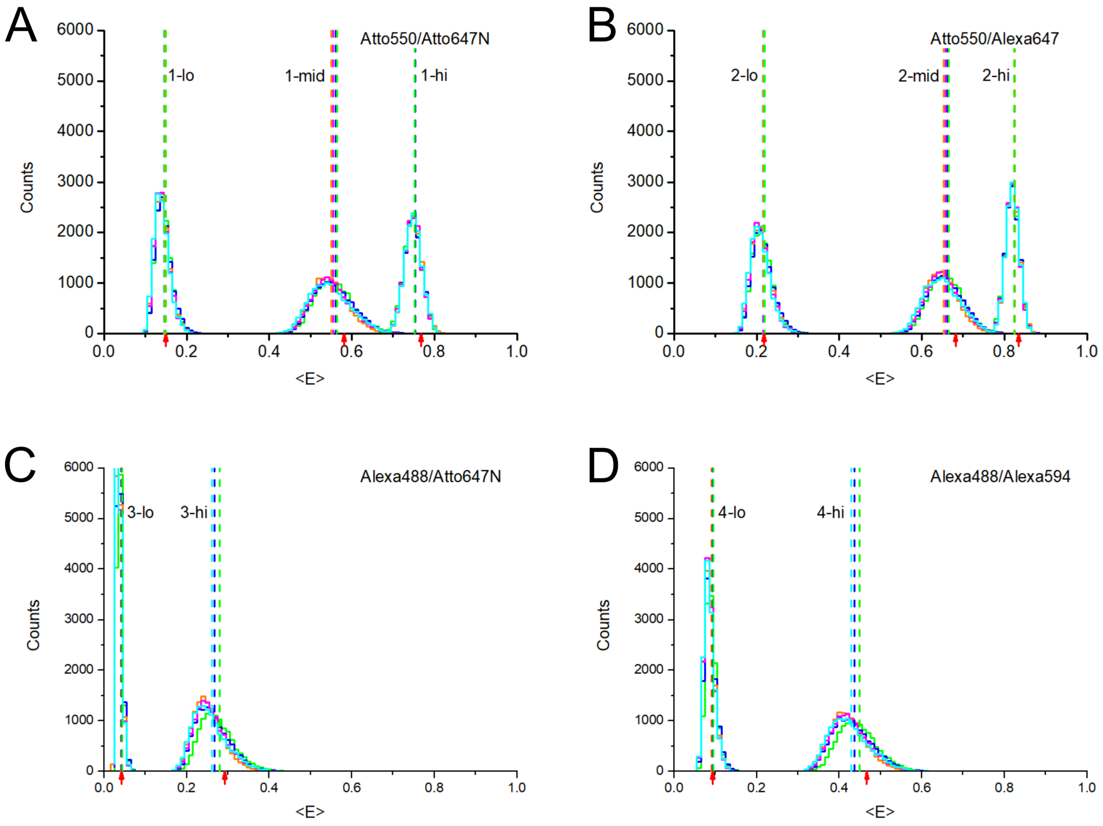

Supplementary Figure N.7.3: Distribution of FRET efficiencies calculated for the conformation extracted from the MD simulations for the four samples (A: Atto550/Atto647N, B: Atto550/Alexa647, C: Alexa488/Atto647N, D: Alexa488/Alexa594). The five colors (green, blue, orange, magenta, and cyan) correspond to the five independent MD simulations performed. Vertical dashed lines indicate the mean of the distribution, while the red arrows below the X-axis indicate the values calculated for the starting structure.

Supplementary Table N.7.1: Comparison of FRET efficiencies $\langle E\rangle_{dyn}$ and corresponding DA distances $R_{\langle E\rangle}^{(dyn)}$ calculated from the five MD simulations to the values from experiments ($\langle E\rangle_{exp}$, $R_{\langle E\rangle}^{(exp)}$)and the static model with $\langle E\rangle_{static}$.

Sample	$\langle E\rangle_{dyn}$ [a]	$\langle E\rangle_{static}$ [b]	$\langle E\rangle_{exp}$ [c]	$R_{\langle E\rangle}^{(dyn)}$ [Å] [d]	$R_{\langle E\rangle}^{(exp)}$ [Å] [e]
**Atto550/Atto647N**					
1-lo	0.15	0.15	0.15 ± 0.02	83.9	83.4 ± 2.5
1-mid	0.56	0.58	0.56 ± 0.03	60.3	60.3 ± 1.3
1-hi	0.75	0.77	0.76 ± 0.02	51.9	51.7 ± 0.9
**Atto550/Alexa647**					
2-lo	0.22	0.22	0.21 ± 0.04	84.2	85.4 ± 3.4
2-mid	0.66	0.68	0.60 ± 0.05	61.0	63.7 ± 2.3
2-hi	0.82	0.83	0.78 ± 0.03	52.6	55.1 ± 1.6
**Alexa488/Atto647N**					
3-lo	0.04	0.04	0.04 ± 0.02	83.1	89.5 ± 12.3
3-mid	0.27	0.29	0.24 ± 0.04	58.4	60.1 ± 2.3
**Alexa488/Alexa594**					
4-lo	0.09	0.09	0.13 ± 0.06	83.5	79.6 ± 6.2
4-mid	0.44	0.47	0.41 ± 0.04	59.5	60.7 ± 1.7

[a] Calculated as average over the five simulations (the standard deviation is in all cases below 0.008).
[b] Calculated for the static starting structure.
[c] From measurements (cf. Supplementary Table 4).
[d] Calculated from $\langle E\rangle_{dyn}$ using Formula (5) in the main text. The SD is not significant (determined by an error propagation from [a].
[e] Calculated from experiments (cf. Supplementary Table 4).

Supplementary Note 8: Description of setups and analysis software
(in alphabetic order)

Birkedal lab (TIRF)

Single-molecule FRET experiments were performed on surface-immobilized molecules using a prism-based total internal reflection microscope with a set up similar to Supplementary Figure 4b. About 5 pM labeled molecules were immobilized inside a coverslide chamber and imaged using alternating laser excitation with 532 and 648 nm diode lasers (Cobolt) or with 514 and 630 nm lasers (Coherent). Fluorescence from the donor and acceptor fluorophores was spatially separated using a wedge mirror (Chroma Technology Corp.) and detected with two color channels onto an EMCCD camera (iXON 3 897, Andor). Details of the experimental setup and immobilization procedures are published elsewhere[29].

Movies were recorded with a 200 ms integration time per frame and analyzed using the iSMS software[30]. The newest version of the software is available at www.isms.au.dk.

Bowen lab (TIRF)

Samples were imaged using a prism-based Total Internal Reflection Fluorescence microscope constructed on an IX71 base with a 60x, 1.2 NA water-immersion objective (Olympus, Center Valley, PA)[31]. Alternating laser excitation, with mechanical shutters (Uniblitz, Rochester, NY), was used to confirm the presence of both a donor and acceptor dye in all molecules used for analysis. Samples were excited with: a laser diode at 635 nm (Newport Corporation, Irvine, CA) for Alexa 647 and Atto 647N; a diode pumped solid-state laser at 532 nm (Newport Corporation, Irvine, CA) for Atto 550; or a laser diode at 473 nm (Photop Technologies Inc. Chasworth, Ca) for Alexa 488. Emission from donor and acceptor was separated using an Optosplit ratiometric image splitter (Cairn Research Ltd, Faversham UK). For experiments with Atto 550 and Alexa 647 (or Atto 647N), we used a 645 nm dichroic mirror with a 585/70 band pass filter for the donor channel and a 670/30 band pass filter for the acceptor channel. For experiments with Alexa 488 and Atto 647N, we used a 593 nm dichroic mirror with a 550/100 band pass filter for the donor channel and a 700/75 band pass filter for the acceptor channel (all filters from Chroma, Bellows Falls, VT). The replicate images were relayed to a single iXon DU-897 EMCCD camera (Andor Technologies, Belfast, UK) at a frame rate of 10 Hz.

Data was processed in home written MATLAB scripts to cross-correlate the replicate images and extract time traces for diffraction limited spots with intensity above baseline[32]. Single molecules were verified by selecting only events showing single step photobleaching to baseline. The γ correction was individually calculated for each selected molecule based upon the relative changes in intensities before and after the photobleaching event.

Cordes Lab (confocal)

Measurements were performed on a confocal setup, as shown in Supplementary Figure 3, but with the addition of a fiber coupling between the laser combining dichroic mirror DM1 and the second dichroic mirror DM2 to improve and clean the beam profile[33].

Elements used were: ZET532/10x (Chroma/AHF) and ZET640/10x (Chroma/AHF) laser clean-up filter (right after Laser 532 and 640 nm); polarization maintaining single-mode fiber P3-488PM-FC-2 (Thorlabs); DM2: Dual line beam splitter ZT532/640rpc (Chroma/AHF); DM3: laser-laser beam splitter H643 LPXR (AHF); FG: BrightLine HC 582/75 (Semrock/AHF) ; FR: Longpass 647 LP Edge Basic (Semroch/AHF); Objective: Super achromat objective UPLSAPO60XW (Olympus) ; Detectors: SPAD SPCM-AQRH-64 (Excelitas); Pinholes: 50 µm; Laser power at sample: ≈ 60 µW for 532 nm and ≈ 25 µW for 640 nm; Beam diameter ≈ 12 mm.

Laser-APD synchronization and readout is performed with NI-Card PCI-6602 (National Instruments) and a LabView (LabVIEW 2009) based home written software[33].

Analysis was also done with a LabView based home written software.

Craggs lab (confocal)

Setup is similar to Supplementary Figure 3, with the following specifications: Lasers used are 515 nm and 635 nm – LuxX plus, precoupled (No DM_1). DM_2 is a Chroma ZT532/640rpc excitation dichroic. The objective O is a Olympus x60 objective UPLSAPO 60XO (WD = 0.17 mm). The lense L is a Edmond Optics 49793 (50 mm focal length). The pinhole PH is a 20 µm (Newport PNH-20). The dichroic DM_3 is a 640 nm longpass (Chroma NC395323 – T640lpxr). The F_G is a Semrock: FF01-582/75-25. The F_R a Semrock: FF01-679/41-25. The APD an Excelitas SPCM-AQRH-14.

The following description is from Bennet et al.[34]:

smFRET data were acquired using a custom built confocal microscope and alternating laser excitation. Two dioide lasers (515 nm and 635 nm – LuxX plus) were directly modulated (100 us, duty cycle 45%) and combined into an optical fibre. The output beam was collimated and then cropped to 2.5 mm diameter by an iris. The beam was directed into the back of the objective (Olympus UPLSAPO 60× NA = 1.35 oil immersion) using a dichroic mirror (Chroma ZT532/640 rpc 3 mm) with the fluorescence emission collected by the same objective, focussed onto a 20 um pinhole and then split (dichroic mirror: Chroma NC395323 – T640lpxr) for detection by two avalanche photodiodes (SPCM-AQRH-14 and SPCM-NIR-14, Excilitas). Photon arrival times were recorded by a national instruments card (PCIe-6353), with the acquisition controlled using custom software (LabView 7.1).

Gratton lab (confocal)

The measurements were done on a modified Olympus FV1000 laser scanning confocal microscope. It is similar to Supplementary Figure 3. Our excitation source was a 20 MHz supercontinuum laser (SC390, Fianium Inc). A filter-wheel with eight interference bandpass filters was used to select desired excitation wavelengths. In our measurements, one set data was collected with the excitation from 530-550nm and the emission from 560-620nm. The other set data was collected with the excitation from 483-493nm and the emission from 505-525nm. An Olympus60× water objective (Olympus UPlanSApo, NA=1.2) was used to focus the laser beam and collect the emission signal.

The internal PMT was modified to send the signal out to a FastFLIM system (ISS Inc), which was synchronized with the 20MHz frequency output from the laser.

Data was then collected and analyzed by SimFCS (available from http://www.lfd.uci.edu/).

Ha lab (TIRF)

Measurements were done on a setup similar to Supplement Figure 4. Instead of using lenses and dichroic mirrors in the excitation pathway to combine the lasers, half-wave plates and a polarizing beam splitter cube were used. The collected fluorescence then passes a slit as shown, and is split with a dichroic and redirected through a lens using only mirrors, so that the two images are put beside each other on the camera chip.

Dichroics in detection: FF640-FDi01-25×36 (Semrock). Filters in detection: BLP02-561R-25 (F1, Semrock) and ZET633TopNotch (F2, Chroma). Objective: water immersion, 60 ×/1.2 NA (Olympus). Camera: EMCCD (iXon 897, Andor). Lasers: 532 nm (Compass 315M, Coherent) and 633 nm (06-MLD, Cobolt).[35]

Data were analyzed by custom-made MatLab codes.

Hendrix lab (confocal)

Our multi-parameter fluorescence detection setup equipped with pulsed interleaved excitation is conceptually identical to the confocal microscope presented in Supplementary Figure 3. Emission from a pulsed 483-nm laser diode (LDH-P-C-470, Picoquant, Berlin, Germany) was cleaned up (Chroma ET485/20x, F49-482, AHF Analysentechnik, Tübingen, Germany), emission from a 635-nm laser diode (LDH-P-C-635B, Picoquant) was cleaned up (Chroma z635/10x, Picoquant) and both lasers were alternated at 26.67 MHz (PDL 828 Sepia2, Picoquant), delayed ~18-ns with respect to each other and combined via a 483-nm-reflecting dichroic mirror in a single-mode optical fiber (coupler: 60FC-4-RGBV11-47, fiber: PMC-400Si-2.6-NA012-3-APC-150-P, Schäfter und Kirchhoff GmbH, Hamburg, Germany). After collimation (60FC-L-4-RGBV11-47, SuK GmbH), the linear polarization was cleaned up (Codixx VIS-600-BC-W01, F22-601, AHF) and the light was reflected on a 3-mm thick excitation polychroic mirror (Chroma zt470-488/640rpc, F58-PQ08, AHF) upward and into the back port of the microscope (IX70, Olympus Belgium NV, Berchem, Belgium) via two mirrors and upward to the sample (3-mm thick Full Reflective Ag Mirror, F21-005, AHF, mounted in a TIRF Filter Cube for BX2/IX2, F91-960, AHF) to the objective (UPLSAPO-60XW, Olympus). Sample emission was transmitted focused through a 75-µm pinhole (P75S, Thorlabs, Munich, Germany) via an achromatic lens (AC254-200-A-ML, Thorlabs), collimated again (AC254-50-A-ML, Thorlabs) and spectrally split (Chroma T560lpxr, F48-559, AHF). The blue range was filtered (Chroma ET525/50m, F47-525, AHF) and polarization was split (PBS251, Thorlabs). The red range was also filtered (Chroma ET705/100m, AHF) and polarization was split (PBS252, Thorlabs). Photons were detected on four avalanche photodiodes (Perkin Elmer or EG&G SPCM-AQR12/14), which were connected to a time-correlated single photon counting (TCSPC) device (SPC-630, Becker & Hickl GmbH, Berlin, Germany) over a router (HRT-82,

Becker & Hickl) and power supply (DSN 102, Picoquant). Signals were stored in 12-bit first-in-first-out (FIFO) files.

All analyses of experimental data were performed in the software package PAM[36]. The software is available as source code, requiring MATLAB to run, or as pre-compiled standalone distributions for Windows or MacOS at http://www.cup.uni-muenchen.de/pc/lamb/software/pam.html or hosted in Git repositories under http://www.gitlab.com/PAM-PIE/PAM and http://www.gitlab.com/PAM-PIE/PAMcompiled. Sample data is provided under http://www.gitlab.com/PAM-PIE/PAM-sampledata. A detailed manual is found under http://pam.readthedocs.io.

Hohlbein lab (TIRF)

Our setup is conceptually identical to the TIRF microscope presented in Supplementary Figure 4 (for TIRF)[37]. For excitation, we used a fibre-coupled laser engine (Omicron, Germany) equipped with four lasers of different wavelengths (405 nm, 473 nm, 561 nm, and 642 nm). A home-written LabVIEW program independently controlled the laser intensities and triggered the camera. The single mode fibre generated a Gaussian shaped beam profile and a point source output at the other end of the fibre. The divergent light is collimated (f = 100 mm, Thorlabs, Germany) and a second lens focuses (f = 200 mm, Thorlabs, Germany) the light back into the back focal plane of a 100x NA 1.49 TIRF objective (Nikon, Japan). A polychroic mirror (zt405/473/561/640rpc, Chroma, USA) and a multibandpass filter (zet405/473/561/640m, Chroma, USA) are used to block any laser light in the emission path. After spatial filtering of the fluorescence with a two-lens system consisting of two tube lenses (f = 200 mm, Thorlabs, Germany) and an adjustable slit (Thorlabs, Germany), the light was spectrally split using two dichroic mirrors (zt561rdc and zt640rdc, Chroma) and a mirror into three beams corresponding to a blue, green, and red fluorescence detection channel. The three beams were then focused (f = 300 mm) on an Ixon Ultra 897 emCCD camera with 512 x 512 pixel (Andor, Northern-Ireland) that was operated in a photon-counting mode.

For image analysis we used a modified version of TwoTone, a freely available, MATLAB-based software package, which identifies molecules and measures the photon counts by fitting the molecular point spread functions to two dimensional Gaussians[38].

Hübner lab (confocal)

The setup was similar to Supplementary Figure 3 with the following components. Donor and acceptor excitation was done with a cw laser at 532 nm (GCL-005-L-LK, Crystalaser, Reno, NV) for the donor and a 635 nm pulsed laser diode (LDH-P-635+Sepia -PDL-808, Picoquant GmbH, Germany) using 100 ps pulses at 10 MHz for the acceptor. Lasers were put through a glass fiber (SMC-460, Schäfter und Kirchhoff) and collimated with UPLSAPO 4X collimation lens. Combining the lasers was accomplished with dichroic mirror Q555LP (Chroma, DM1). Dichroic DM2 was a Z532/633 (Chroma). Fluorescence collection and focusing was done with a CFI Plan Apo VC 60XWI (Nikon) objective. Lenses L1 and L2 focussing the fluorescence on a 50 µm pinhole (P50H, Thorlabs) were two tube lenses (f=200 mm, MXA20696, Nikon). Fluorescence

separation was done with dichroic 640DCXR (Chroma, DM3). For fluorescence clean-up the filters FF01-582/75 (Semrock) were used for donor and HQ650/100 (Chroma) for acceptor fluorescence. The signal was then collected with SPCM-AQRH-14 (Excelitas) APDs. TCSPC electronics were a TimeHarp 200 (Picoquant).
For the measurements a home written software based on LabView (National Instruments) and for the data reduction an Igor Pro (Wavemetrics) based home written software was used.

Kapanidis lab (confocal)
smFRET experiments were carried out on a custom-built confocal microscope[39,40], as shown schematically in Supplementary Figure 3. The setup was modified to allow ALEX of donor and acceptor fluorophores. Custom-written LabVIEW software was used to register and evaluate the detected signal.
Data analysis was carried out using custom-written Matlab software[41].

Lamb lab (confocal)
Single-molecule FRET experiments with pulsed-interleaved excitation (PIE) and multiparameter fluorescence detection (MFD) were performed on a homebuilt confocal microscope as described previously[42]. In addition to the schematic shown in Supplementary Figure 3, a polarizing beam splitter is installed after the confocal pinhole to split the signal by polarization before the dichroic mirror (640DCXR, AHF Analysentechnik). Pulsed-interleaved excitation was performed at 532 nm (PicoTA 530, PicoQuant) and 640 nm (LDH-D-C640, PicoQuant) at a repetition rate of 26.67 MHz with a delay of ~18 ns and a laser power of 100 µW. Fluorescence emission was filtered (donor: Brightline HQ582/75, acceptor: Brightline HQ700/75, AHF Analysentechnik), focused on avalanche photodiodes (SPCM-AQR, Perkin-Elmer) and recorded on single-photon-counting cards (SPC-154, Becker&Hickl). Data analysis was performed using the *PAM* software package written in MATLAB (The MathWorks)[36]. Single-molecule events were identified using a sliding-time-window burst search algorithm with a countrate threshold of 10 kHz, a time window of 500 µs and a minimum photon number of 100. To remove photoblinking and -bleaching events, the ALEX-2CDE filter was applied using an upper threshold of 10 (ref. [43]).

Lee lab (confocal)
We performed smFRET measurement using a home-built confocal microscope, similar with the setup described in Supplementary Figure 3, which has been well described in our previous works[44,45]. The alternation of two lasers (ALEX) was achieved using acoustic-optic modulators. The data acquisition and analysis were performed using a home-built software based on LabVIEW program as described before[46].

Lemke lab (confocal)
The setup was as described in Supplementary Figure 3 and previously in detail in refs. [47,48], with the following elements changed. Lasers were a LDH 485 (Picoquant) and a SuperK Extreme (NKT Potonics) filtered with a 572/15 bandpass alternating at 26,6 Mhz and combined onto the laser path with DM1= R488-Di01. Dichroic Mirror DM2 was a ZT 488/561/660 (AHF) and for DM3 a zt

561 RDC-UF (Chroma) and a FF650-D01 (Semrock) were used in sequence for three detection channels. For the three detection channels the filters 525/50 ET (Semrock), 620/60 ET (Chroma) and 700/75 ET (Chroma) were used. The analysis was done using self-written code in IgorPro (Wavemetrics) following procedures described in detail in refs. [49,50].

Levitus Lab (confocal)

Fluorescence intensity decays were acquired at room temperature using the timecorrelated single photon counting technique. A fiber supercontinuum laser (Fianium SC450) was used as the excitation source. The laser provides 6 ps pulses at a variable repetition rate, set at 20 MHz. The laser output was sent through an acousto-optical tunable filter (Fianium AOTF) to obtain 552 nm excitation. Fluorescence emission was collected at a 90° angle and detected using a double-grating monochromator (Jobin-Yvon, Gemini-180) and a microchannel plate photomultiplier tube (Hamamatsu R3809U-50). The emission monochromator was set to 580 nm. The polarization of the emission was collected at the magic angle relative to the excitation. A single photon counting card (Becker-Hickl, SPC830) was used for data acquisition. The IRF was measured with a 3% Ludox scattering solution (Sigma-Aldrich, MO) and had a fwhm of approximately 80 ps when measured at 552 nm. The data were deconvoluted and fitted with a sum of exponential terms using software written in-house (ASUFIT). The quality of the fit was evaluated based on the residuals.

Michaelis lab (Confocal)

The confocal setup used for this study has been described in detail recently[51]. It resembles the one depicted in supplementary figure 3, but with the major addition of polarization sensitivity. A polarizing beam splitting cube positioned after the collimating lens behind the pinhole splits the light into its components parallel and perpendicular with respect to the excitation light. Each of the two resulting beams is then split by a dichroic mirror and focused onto an APD after passing an emission filter, similar to what is shown in supplementary figure 3. Differing from Schwarz et al. 2018, a different polychroic mirror was used during the initial measurements of samples 1-lo, 1-mid, 2-lo and 2-mid (Triple Line zt488/532/658, AHF Analysentechnik AG, Tübingen, Germany). The setup also provides an additional 488nm laser line and the filters and dichroic mirrors can be exchanged in order to perform smFRET experiments with a 488nm-excitable donor and a 647nm-excitable acceptor (e.g. samples 3-lo and 3-mid described in supplementary note 6).

For data analysis, an earlier version of the software package PAM (PIE analysis with MATLAB) was used [36]. The newest version of the software is available via https://gitlab.com/PAM-PIE/PAM.

Michaelis lab (TIRF)

All TIRF-measurements were conducted using a custom-build prism-type TIRF setup which has been described in detail recently[52]. The setup is similar to the setup described in Supplementary Figure 4, only with some minor additions namely an acousto-optic tunable filter (AOTFnC-VIS, AA *Opto-Electronic*) and an IR-laser based auto-focus system. The AOTF allows for the selection of the excitation wavelength and the control of laser intensity and duration.

The acquired data was analyzed using a custom-written software called *SM-FRET* which was described in detail[52].

Sanabria Lab (confocal)

The home built confocal system and data analysis at the Sanabrias' lab was recently described in detail[53,54]. It is similar to the one described in Supplementary Figure 3, but with four detectors and different spectral windows. Differences are briefly described below. The microscope body is an Olympus IX-73 with a 60X, 1.2 NA collar (0.17) corrected Olympus objective. It uses Pulsed Interleaved Excitation (PIE)[42] with diode lasers at 485 nm and 640 nm (PicoQuant, Germany) operated at 40 MHz with 25 ns interleaved time. The power at the objective was 120 μW at 485 nm and 39 μW at 640 nm. Emitted photons were collected through the same objective and spatially filtered through a 70 μm pinhole to limit the effective confocal detection volume. Fluorescence emission is separated into parallel and perpendicular polarization components at two different spectral windows using band pass filters ET525/50 and ET720/150 (Chroma Technology Co.) for donor and acceptor, respectively. In total, four photon-detectors are used—two for donor (PMA Hybrid model 40 PicoQuant, Germany) and two for acceptor (PMA Hybrid model 50, PicoQuant, Germany). To insure temporal data registration of the 4 synchronized input channels, we used a HydraHarp 400 TCSPC module (PicoQuant, Germany) in Time-Tagged Time-Resolved mode.
Data analysis uses Multiparameter Fluorescence Detection software suit developed at the Seidel's lab (http://www.mpc.hhu.de/software/software-package.html).

Schlierf Lab (confocal)

Observations of single-molecule fluorescence were made on a custom-built dual-color and dual-polarization confocal setup based on an inverted microscope (Eclipse Ti-E, Nikon, Tokyo, Japan) as previously described in[55,56]. Briefly, donor and acceptor fluorophores were excited with linearly polarized 530-nm and 640-nm picosecond pulsed laser sources (LDH-P-FA-530L and LDH-D-C-640, both from PicoQuant, Berlin, Germany) driven in pulsed interleaved excitation mode at a total repetition rate of 50 MHz. The laser beams were coupled to a polarization-maintaining single-mode optical fiber (P3-488PM-FC-2, Thorlabs, NJ, USA), collimated (60FC-T-4-RGBV42-47, Schäfter und Kirchhoff, Hamburg, Germany), and focused by a water immersion objective (CFI Plan Apo WI 60x, NA 1.2, Nikon). Emitted fluorescent light was collected by the same objective, separated from the excitation light by a dual-edge dichroic mirror (zt532/642rpc, Chroma, Bellows Falls, VT, USA), and focused on a 50-μm pinhole (Thorlabs). Donor and acceptor photons were spectrally separated by single-edge dichroic mirrors (FF650-Di01, Semrock, USA) after a polarizing beam splitter (CM1-PBS251, Thorlabs), band-pass-filtered (FF01-582/75, Semrock, Rochester, NY, USA; ET700/75M, Chroma), and focused onto four single-photon-counting avalanche diodes (τ-SPADs, PicoQuant). Photons were registered by four individual time-correlated single-photon counting modules (Hydra Harp, PicoQuant) with a time resolution of 16 ps. Synchronization with the lasers for alternating excitation was accomplished with the aid of a diode laser driver (PDL828, PicoQuant).

Data analysis was performed with custom-written Matlab scripts (Mathworks, USA) and single-molecule events were identified from the acquired photon stream by a burst search algorithm as described in[57,58]. The analysis software is available upon request.

Schuler Lab (confocal)

A commercial confocal instrument (MT200, PicoQuant, Berlin) or a custom-built instrument[59]. were used for the measurements. Both instruments were equipped with an UplanApo 60×/1.20-W objective (Olympus), a 100-µm confocal pinhole, and HydraHarp 400 counting electronics (PicoQuant, Berlin). They were operated with pulsed interleaved excitation (20 MHz) in a configuration similar to that shown in Supplementary Figure 3 with the following components.

In the MT200 setup a 485-nm pulsed diode laser (LDH-D-C-485, Picoquant) was used for donor excitation, and a SC-450-4 supercontinuum fiber laser (Fianium) filtered with a z582/15 bandpass filter (Chroma) for acceptor excitation. Dichroic mirror DM2 was a BS R405/488/594 (Semrock) and DM3 a 585DCXR (Chroma). Donor fluorescence was filtered with a ET 525/50 (Chroma) and recorded with a SPCM-AQRH-14 (PerkinElmer Optoelectronics) APD. Acceptor fluorescence was filtered with an HQ 650/100 (Chroma) and recorded with a SPCM-AQR-14 APD (PerkinElmer Optoelectronics) APD.

In the custom built setup, the donor was excited with a SC-450-4 supercontinuum fiber laser (Fianium) filtered with a BrightLine HC 520/5 band-pass filter (Semrock) and acceptor excitation was done with a 635nm pulsed diode laser (LDH-D-C-635M, PicoQuant). In this instrument, the dichroic mirrors here were a zt405/530/630rpc (Chroma) for DM2 and a 635DCXR (Chroma) for DM3. Donor fluorescence was filtered with an ET585/65m (Chroma) and recorded with a τ-SPAD (PicoQuant). Acceptor fluorescence was cleaned up with LP647RU and HC750/SP (Chroma) filters and recorded with an SPCM-AQR-14 APD (PerkinElmer Optoelectronics). Data were analyzed using custom-developed software written in C++ and Mathematica.

Seidel Lab

Ensemble Time Correlated Single Photon Counting (eTCSPC)

Fluorescence lifetime decays were recorded by FT300 setup (PicoQuant, Germany) using a white light laser from NKT Photonics (Germany) with repetition rate 20 MHz for excitation. All samples were measured in Quartz Ultra-Micro-cuvettes (Helma #105.252.85.40), with a total sample volume of 20µl. A Ludox scattering solution was used to record the instrument response function (IRF). The detailed measurement conditions for the experiments are provided in the Table N8.1.

Table N8.1: Settings for Picoquant FT300 setup

Settings/dye	Atto 550	Alexa 488	Atto 647N	Alexa647	Alexa 594
Excitation, nm	552	485	635	635	590
Emission, nm	580	520	665	665	617
Bandpass, nm	5.4	9.2	9.2	8.1	8.1
Excitation filter	none	ZET 488/10x	ZET 635/20x	ZET 635/20x	none
Emission filter	FGL 570	FGL 515	FGL 645	FGL 645	FGL 610

Confocal setup 1 (for samples labelled with Alexa488-Atto647N)

The general scheme of the setup is described by Sisamakis et al[50] (see Fig. 18.5 therein). All sample solutions were measured in NUNC chambers (Lab-Tek, Thermo Scientific, Germany) with 300 µL sample volume. The fluorescent donor molecules (Alexa 488) are excited by a pulsed diode laser (LDH-D-C 485, PicoQuant), at 485 nm operated at 64 MHz, 110 µW at the sample in one color excitation experiment or at 32 MHz in PIE experiment, 110µW at the sample. The laser light is guided into the epi-illuminated confocal microscope (Olympus IX71, Hamburg, Germany) by dichroic beamsplitter FF500/646-Di01 (Semrock, USA) focussed by a water immersion objective (UPlanSApo 60x/1.2w, Olympus Hamburg, Germany). In PIE experiments the fluorescent acceptor molecules (Atto647N) are additionally excited by 635 nm pulsed diode laser (LDH-D-C 640, PicoQuant). The emitted fluorescence is collected through the objective and spatially filtered using a pinhole with typical diameter with 100 µm. Then, the signal is split into parallel and perpendicular components via a polarizing beam splitter and then at two different spectral windows (e.g. "green" and "red") and then split again using 50/50 beam splitters resulting in a total of eight detection channels. Additionally green (HQ 520/35 nm for Alexa488) from AHF, Tübingen, Germany and red (HQ 720/150 nm for Atto647N) bandpass filters (AHF, Tübingen, Germany) are placed in front of the detectors to provide the registration only of the fluorescence photons coming from the acceptor and donor molecules. Detection is performed using eight avalanche photodiodes (4 green channels: τ-SPAD (PicoQuant, Germany) and 4 red channels: AQR 14 (Perkin Elmer). The detector outputs were recorded by a TCSPC module (HydraHarp 400, PicoQuant).

Confocal setup 2 (for samples labelled with Atto550-Atto647N)

The confocal setup 2 is similar to the confocal setup 1 described above. It has the following components (only the differences are mentioned):

Confocal microscope: Olympus IX71 (Hamburg, Germany).

Objective: Olympus UPlanSApo 60x/1.2w (Hamburg, Germany).

Dichroic Beamsplitter: F68-532_zt532/640NIRrpo (AHF, Tübingen, Germany).

Fluorescence dichroic beamsplitter: T640lpxr (AHF, Tübingen, Germany).

Diode lasers: 530 nm (LDH-P-FA 530B, PicoQuant) and 640 nm (LDH-D-C 640, PicoQuant), both with a repetition rate 32 MHz and with a power 75.5 µW and 16.7 µW at the sample, respectively.

Bandpass filters: green ET595/50 and red HQ730/140.

2 green and 2 red detectors: both SPCM-AQRH 14 (Excelitas, USA).

Confocal setup 3 (for samples labelled with Alexa488-Alexa594)

The confocal setup 3 is similar to the confocal setup 1 described above. It has the following components (only the differences are mentioned):

Confocal microscope (Olympus IX70, Hamburg, Germany).

Dichroic beam splitter: Q505LP (AHF, Tübingen, Germany).

Fluorescence dichroic beamsplitter: 595 LP DCXR (AHF, Tübingen, Germany).

Diode lasers: 495 nm (PicoQuant, Germany) with a repetition rate of 32 MHz and a power at the sample of 110 µW.

Bandpass filters: green HQ520/66, red HQ630/60.

2 green and 2 red detectors: SPCM-AQRH 14 (Perkin Elmer).
TCSPC module SPC 132 (Becker&Hickl, Germany).

The recorded data were analyzed with a home-written LabView software that was developed in the Seidel lab and is described in ref. [50]. The software is available on the homepage of the Seidel group (http://www.mpc.hhu.de/software/software-package.html).
To analyze the recorded fluorescence bursts, a burst search algorithm according to reference [60] was applied. The confocal setups were calibrated by the PIE measurements as described in this work or by FRET-lines relating the donor fluorescence lifetime to the intensity-based FRET efficiency as described in ref. [61].

Tinnefeld lab (confocal)
The measurements were carried out on a custom-built confocal microscope [62] based on an IX 71 (Olympus) similar to the setup shown in Supplementary Figure 3 with alternating laser excitation. Pulsed Lasers (637 nm, 80 MHz, LDH-D-C-640; 532 nm 80 MHz, LDH-P-FA-530B; both PicoQuant) were powered by a Sepia 2 (PicoQuant) unit. Both lasers were combined by a dichroic mirror (640 LPXR, AHF). In addition to the setup shown in Supplementary Figure 3, the lasers were alternated by an acousto optical tunable filter (AOTFnc-VIS, AA optoelectronic) and coupled into a single mode fiber (P3-488PM-FC-2, Thorlabs) to obtain a Gaussian beam profile. After a linear polarizer (LPVISE100 A, Thorlabs) and lambda quarter plate (AQWP05M 600, Thorlabs), circular polarized light was obtained. After a dual band dichroic beam splitter (z532/633, AHF), the light was focused by an oil-immersion objective (UPLSAPO 100XO, NA 1.40, Olympus). The emitted light was collected by the same objective and focused on a 50 µm pinhole (Linos). Subsequently, the fluorescence was split by a dichroic mirror (640DCXR, AHF) into a green (Brightline HC582/75, AHF; RazorEdge LP 532, Semrock) and red (Bandpass ET 700/75m, AHF; RazorEdge LP 647, Semrock) detection channel. Two SPADs (τ-SPAD 100, PicoQuant) accounted for the detection. The SPAD signals were registered by a TCSPC card (SPC-830, Becker&Hickl). The setup was controlled with custom-made LabView (National Instruments) software. Recorded data were analyzed with a LabView software [63]. To analyze the recorded fluorescence bursts, a burst search algorithm according to reference [64] was applied.

Weninger lab (TIRF)
The microscope is similar to that in Supplementary Figure 4 [31]. Briefly, we illuminate immobilized samples at the surface of a quartz microscope slide with prism-type total internal reflection of laser beams at the quartz/buffer interface. Alternating illumination of 532 nm or 640 nm allows sequential excitation of donor and acceptor dyes when using Atto 550, Alexa 647 and Atto 647N. Fluorescence emission is collected by a 1.20 N.A. water-immersion microscope objective (Olympus UIS2 UPlanSApo 60x/1.20 W). The fluorescence image is spectrally divided with a Dualview splitter (DV2, Photometrics) incorporating a 645dcxr dichroic mirror with a 585/70 bandpass filter (donor) and a 700/75 bandpass filter (acceptor) (all from Chroma Technology Corp.). The spectrally divided image is detected with an emCCD (Cascade 521B, Photometrics) operating at 10 Hz.

We use home-written analysis software implemented in MATLAB (MathWorks)[32]. Immobilized single molecules are detected in a ten-frame averaged image as pixels of maximum intensity above an empirically determined threshold (typically based on the statistics of all pixels in the field of view as 7 standard deviations above the average), separated by five or more pixels from any neighboring maxima. Fluorescence intensity for a single molecule is extracted from each frame of a movie as the sum of the 4 brightest pixels in a 3x3 pixel region centered on the local maximum. Before a FRET experiment, we acquire a spectrally split image of a field of immobilized, fluorescent 100 nm diameter polystyrene spheres that emit broadly into both channels to build a mapping between donor and acceptor channels using the MATLAB image processing toolbox command cp2tform. This mapping is applied to movies containing FRET data to obtain donor and acceptors intensities from immobilized single molecules. Background for each detected molecule is calculated locally as the median value of the 16x16 pixel region around the identified peak pixel. The background value is subtracted from the single molecule intensity values. The background value is verified by requiring the background-subtracted intensity time-trace for a single molecule emission return to zero upon a single step photobleaching event. Leakage between donor and acceptor channels is measured separately using singly labeled samples. FRET efficiency is then calculated as described in the main text.

Bibliography:

1. Sindbert, S. *et al.* Accurate Distance Determination of Nucleic Acids via Förster Resonance Energy Transfer: Implications of Dye Linker Length and Rigidity. *J. Am. Chem. Soc.* **133**, 2463-2480 (2011).
2. Widengren, J. & Schwille, P. Characterization of photoinduced isomerization and back-isomerization of the cyanine dye Cy5 by fluorescence correlation spectroscopy. *J. Phys. Chem. A* **104**, 6416-6428 (2000).
3. Widengren, J., Schweinberger, E., Berger, S. & Seidel, C. A. M. Two new concepts to measure fluorescence resonance energy transfer via fluorescence correlation spectroscopy: Theory and experimental realizations. *J. Phys. Chem. A* **105**, 6851-6866 (2001).
4. Hendrix, J. & Lamb, D. C. Pulsed Interleaved Excitation: Principles and Applications. *Fluorescence Fluctuation Spectroscopy (Ffs), Pt A* **518**, 205-243 (2013).
5. Margeat, E. *et al.* Direct Observation of Abortive Initiation and Promoter Escape Within Single Immobilized Transcription Complexes. *Biophys. J.* **90**, 1419-1431 (2006).
6. Peulen, T. O., Opanasyuk, O. & Seidel, C. A. M. Combining graphical and analytical methods with molecular simulations to analyze time-resolved FRET-measurements of labeled macromolecules accurately. *J. Phys. Chem. B* **121**, 8211-8241 (2017).
7. Wozniak, A. K., Schröder, G. F., Grubmüller, H., Seidel, C. A. M. & Oesterhelt, F. Single-Molecule FRET Measures Bends and Kinks in DNA. *Proc. Natl. Acad. Sci. USA* **105**, 18337-18342 (2008).
8. Ivani, I. *et al.* Parmbsc1: a refined force field for DNA simulations. *Nat. Meth.* **13**, 55-58 (2016).
9. Muschielok, A. *et al.* A Nano-Positioning System for Macromolecular Structural Analysis. *Nat. Meth.* **5**, 965-971 (2008).
10. Kalinin, S. *et al.* A toolkit and benchmark study for FRET-restrained high-precision structural modeling. *Nat. Meth.* **9**, 1218-1227 (2012).

11. Hellenkamp, B., Wortmann, P., Kandzia, F., Zacharias, M. & Hugel, T. Multidomain Structure and Correlated Dynamics Determined by Self-Consistent FRET Networks. *Nat. Meth.* **14**, 174-180 (2017).
12. Beckers, M., Drechsler, F., Eilert, T., Nagy, J. & Michaelis, J. Quantitative Structural Information from Single-Molecule FRET. *Farad. Discuss.* **184**, 117-129 (2015).
13. Dimura, M. *et al.* Quantitative FRET Studies and Integrative Modeling Unravel the Structure and Dynamics of Biomolecular Systems. *Curr. Opin. Struct. Biol.* **40**, 163-185 (2016).
14. Nagy, J., Eilert, T. & Michaelis, J. Precision and Accuracy in smFRET based structural studies - a benchmark study of the FAST-Nano-Positioning System. *J. Chem. Phys.* **148** (2018).
15. Treutlein, B. *et al.* Dynamic architecture of a minimal RNA polymerase II open promoter complex. *Mol. Cell* **46**, 136-146 (2012).
16. Hohlbein, J. *et al.* Conformational landscapes of DNA polymerase I and mutator derivatives establish fidelity checkpoints for nucleotide insertion. *Nat. Commun.* **4**, 2131 (2013).
17. Tyagi, S. & Lemke, E. A. Genetically encoded click chemistry for single-molecule FRET of proteins. *Methods Cell Biol.* **113**, 169-187 (2013).
18. Tyagi, S. & Lemke, E. A. Single-molecule FRET and crosslinking studies in structural biology enabled by noncanonical amino acids. *Curr. Opin. Struct. Biol.* **32**, 66-73 (2015).
19. Eilert, T., Beckers, M., Drechsler, F. & Michaelis, J. Fast-NPS - A Markov chain Monte Carlo-based analysis tool to obtain structural information from single-molecule FRET measurements. *Comput. Phys. Commun.* **219**, 377-389 (2017).
20. Lipfert, J. *et al.* Double-stranded RNA under force and torque: Similarities to and striking differences from double-stranded DNA. *Proc. Natl. Acad. Sci. USA* **111**, 15408-15413 (2014).
21. Liebl, K., Drsata, T., Lankas, F., Lipfert, J. & Zacharias, M. Explaining the striking difference in twist-stretch coupling between DNA and RNA: A comparative molecular dynamics analysis. *Nucleic Acids Res.* **43**, 10143-10156 (2015).
22. Stelzl, L. S., Erlenbach, N., Heinz, M., Prisner, T. F. & Hummer, G. Resolving the conformational dynamics of DNA with Ångström resolution by pulsed electron-electron double resonance and molecular dynamics. *J. Am. Chem. Soc.* **139**, 11674-11677 (2017).
23. Vafabakhsh, R. & Ha, T. Extreme Bendability of DNA Less than 100 Base Pairs Long Revealed by Single-Molecule Cyclization. *Science* **337**, 1097-1101 (2012).
24. Case, D. A. *et al.* AMBER 2016. (University of California, San Francisco, 2016).
25. Jorgensen, W. L., Chandrasekhar, J., Madura, J. D., Impey, R. W. & Klein, M. L. Comparison of Simple Potential Functions for Simulating Liquid Water. *J. Chem. Phys.* **79**, 926-935 (1983).
26. Joung, I. S. & Cheatham, T. E. Determination of alkali and halide monovalent ion parameters for use in explicitly solvated biomolecular simulations. *J. Phys. Chem. B* **112**, 9020-9041 (2008).
27. Li, P. F., Roberts, B. P., Chakravorty, D. K. & Merz, K. M. Rational design of particle mesh ewald compatible Lennard-Jones parameters for +2 metal cations in explicit solvent. *J. Chem. Theory Comput.* **9**, 2733-2748 (2013).
28. Gohlke, H., Kiel, C. & Case, D. A. Insights into protein-protein binding by binding free energy calculation and free energy decomposition for the Ras-Raf and Ras-RalGDS complexes. *J. Mol. Biol.* **330**, 891-913 (2003).
29. Kruger, A. C., Hildebrandt, L. L., Kragh, S. L. & Birkedal, V. Structural dynamics of nucleic acids by single-molecule FRET. *Methods Cell Biol.* **113**, 1-37 (2013).
30. Preus, S., Noer, S. L., Hildebrandt, L. L., Gudnason, D. & Birkedal, V. iSMS: single-molecule FRET microscopy software. *Nat Methods* **12**, 593-594 (2015).
31. McCann, J. J., Choi, U. B., Zheng, L., Weninger, K. & Bowen, M. E. Optimizing Methods to Recover Absolute FRET Efficiency from Immobilized Single Molecules. *Biophys. J.* **99**, 961-970 (2010).

32. Choi, U. B., Weninger, K. R. & Bowen, M. E. Immobilization of proteins for single-molecule fluorescence resonance energy transfer measurements of conformation and dynamics. *Methods Mol. Biol.* **896**, 3-20 (2012).
33. Gouridis, G. *et al.* Conformational dynamics in substrate-binding domains influences transport in the ABC importer GlnPQ. *Nat. Struct. Mol. Biol.* **22**, 57-64 (2015).
34. Bennet, I. A. *et al.* Regional conformational flexibility couples substrate specificity and scissile phosphate diester selectivity in human flap endonuclease 1. *Nucleic Acids Res.* **46**, 5618-5633 (2018).
35. Hua, B. *et al.* An improved surface passivation method for single-molecule studies. *Nat Methods* **11**, 1233-1236 (2014).
36. Schrimpf, W., Barth, A., Hendrix, J. & Lamb, D. C. PAM: A Framework for Integrated Analysis of Imaging, Single-Molecule, and Ensemble Fluorescence Data. *Biophys. J.* **114**, 1518-1528 (2018).
37. Farooq, S. & Hohlbein, J. Camera-based single-molecule FRET detection with improved time resolution. *Phys. Chem. Chem. Phys.* **17**, 27862-27872 (2015).
38. Holden, S. J. *et al.* Defining the limits of single-molecule FRET resolution in TIRF microscopy. *Biophys. J.* **99**, 3102-3111 (2010).
39. Santoso, Y. *et al.* Conformational transitions in DNA polymerase I revealed by single-molecule FRET. *Proc Natl Acad Sci U S A* **107**, 715-720 (2010).
40. Robb, N. C. *et al.* The transcription bubble of the RNA polymerase-promoter open complex exhibits conformational heterogeneity and millisecond-scale dynamics: implications for transcription start-site selection. *J. Mol. Biol.* **425**, 875-885 (2013).
41. Tomescu, A. I., Robb, N. C., Hengrung, N., Fodor, E. & Kapanidis, A. N. Single-molecule FRET reveals a corkscrew RNA structure for the polymerase-bound influenza virus promoter. *Proc Natl Acad Sci U S A* **111**, E3335-3342 (2014).
42. Kudryavtsev, V. *et al.* Combining MFD and PIE for Accurate Single-Pair Förster Resonance Energy Transfer Measurements. *ChemPhysChem* **13**, 1060-1078 (2012).
43. Tomov, T. E. *et al.* Disentangling Subpopulations in Single-Molecule FRET and ALEX Experiments with Photon Distribution Analysis. *Biophys. J.* **102**, 1163-1173 (2012).
44. Kim, C., Kim, J. Y., Kim, S. H., Lee, B. I. & Lee, N. K. Direct characterization of protein oligomers and their quaternary structures by single-molecule FRET. *Chem Commun (Camb)* **48**, 1138-1140 (2012).
45. Kim, J. Y. *et al.* Solution single-vesicle assay reveals PIP2-mediated sequential actions of synaptotagmin-1 on SNAREs. *EMBO J.* **31**, 2144-2155 (2012).
46. Lee, N. K. *et al.* Accurate FRET Measurements Within Single Diffusing Biomolecules Using Alternating-Laser Excitation. *Biophys. J.* **88**, 2939-2953 (2005).
47. Milles, S., Koehler, C., Gambin, Y., Deniz, A. A. & Lemke, E. A. Intramolecular three-colour single pair FRET of intrinsically disordered proteins with increased dynamic range. *Mol Biosyst* **8**, 2531-2534 (2012).
48. Milles, S. & Lemke, E. A. Single Molecule Study of the Intrinsically Disordered FG-Repeat Nucleoporin 153. *Biophys. J.* **101**, 1710-1719 (2011).
49. Eggeling, C. *et al.* Data registration and selective single-molecule analysis using multi-parameter fluorescence detection. *J. Biotechnol.* **86**, 163-180 (2001).
50. Sisamakis, E., Valeri, A., Kalinin, S., Rothwell, P. J. & Seidel, C. A. M. Accurate Single-Molecule FRET Studies Using Multiparameter Fluorescence Detection. *Methods Enzymol.* **475**, 455-514 (2010).
51. Schwarz, M. *et al.* Single-molecule nucleosome remodeling by INO80 and effects of histone tails. *FEBS Lett.* **592**, 318-331 (2018).

52. Dörfler, T., Eilert, T., Rocker, C., Nagy, J. & Michaelis, J. Structural Information from Single-molecule FRET Experiments Using the Fast Nano-positioning System. *Jove-Journal of Visualized Experiments* (2017).
53. Dolino, D. M., Rezaei Adariani, S., Shaikh, S. A., Jayaraman, V. & Sanabria, H. Conformational selection and submillisecond dynamics of the ligand-binding domain of the N-methyl-D-Aspartate receptor. *J Biol Chem* **291**, 16175-16185 (2016).
54. Ma, J. *et al.* High Precision FRET at Single-molecule Level for Biomolecule Structure Determination. *J Vis Exp*, e55623 (2017).
55. Hartmann, A., Krainer, G. & Schlierf, M. Different fluorophore labeling strategies and designs affect millisecond kinetics of DNA hairpins. *Molecules* **19**, 13735-13754 (2014).
56. Krainer, G., Hartmann, A. & Schlierf, M. farFRET: Extending the Range in Single-Molecule FRET Experiments beyond 10 nm. *Nano Lett* **15**, 5826-5829 (2015).
57. Hartmann, A., Krainer, G., Keller, S. & Schlierf, M. Quantification of Millisecond Protein-Folding Dynamics in Membrane-Mimetic Environments by Single-Molecule Forster Resonance Energy Transfer Spectroscopy. *Anal. Chem.* **87**, 11224-11232 (2015).
58. Krainer, G., Hartmann, A., Anandamurugan, A., Gracia, P. & Schlierf, M. Ultrafast protein folding through polar interactions in membrane-mimetic environments. *J. Mol. Biol.* **430** (2018).
59. König, I. *et al.* Single-molecule spectroscopy of protein conformational dynamics in live eukaryotic cells. *Nat. Meth.* **12**, 773-779 (2015).
60. Fries, J. R., Brand, L., Eggeling, C., Kollner, M. & Seidel, C. A. M. Quantitative identification of different single molecules by selective time-resolved confocal fluorescence spectroscopy. *J. Phys. Chem. A* **102**, 6601-6613 (1998).
61. Kalinin, S., Valeri, A., Antonik, M., Felekyan, S. & Seidel, C. A. M. Detection of Structural Dynamics by FRET: A Photon Distribution and Fluorescence Lifetime Analysis of Systems with Multiple States. *J. Phys. Chem. B* **114**, 7983-7995 (2010).
62. Nickels, P. C. *et al.* Molecular force spectroscopy with a DNA origami-based nanoscopic force clamp. *Science* **354**, 305-307 (2016).
63. Kapanidis, A. N. *et al.* Fluorescence-aided molecule sorting: Analysis of structure and interactions by alternating-laser excitation of single molecules. *Proc. Natl. Acad. Sci. USA* **101**, 8936-8941 (2004).
64. Nir, E. *et al.* Shot-Noise Limited Single-Molecule FRET Histograms: Comparison Between Theory and Experiments. *J. Phys. Chem. B* **110**, 22103-22124 (2006).
