# Supplementary material for: Precision and accuracy of single-molecule FRET measurements—a multi-laboratory benchmark study
Source: Nat Methods. 2018 Aug 31;15(9):669–76. doi: 10.1038/s41592-018-0085-0 (PMC6121742; doi:10.1038/s41592-018-0085-0)
Supplement: Supplementary file 10 — Reporting Summary [file 41592_2018_85_MOESM2_ESM.pdf]

nature research

Corresponding author(s): Thorsten Hugel
Tim Craggs
Claus Seidel
Jens Michaelis

Reporting Summary

Nature Research wishes to improve the reproducibility of the work that we publish. This form provides structure for consistency and transparency in reporting. For further information on Nature Research policies, see Authors & Referees and the Editorial Policy Checklist.

Statistical parameters

When statistical analyses are reported, confirm that the following items are present in the relevant location (e.g. figure legend, table legend, main text, or Methods section).

n/a	Confirmed	
☐	☒	The exact sample size (*n*) for each experimental group/condition, given as a discrete number and unit of measurement
☐	☒	An indication of whether measurements were taken from distinct samples or whether the same sample was measured repeatedly
☒	☐	The statistical test(s) used AND whether they are one- or two-sided<br>*Only common tests should be described solely by name; describe more complex techniques in the Methods section.*
☒	☐	A description of all covariates tested
☒	☐	A description of any assumptions or corrections, such as tests of normality and adjustment for multiple comparisons
☐	☒	A full description of the statistics including central tendency (e.g. means) or other basic estimates (e.g. regression coefficient) AND variation (e.g. standard deviation) or associated estimates of uncertainty (e.g. confidence intervals)
☒	☐	For null hypothesis testing, the test statistic (e.g. *F*, *t*, *r*) with confidence intervals, effect sizes, degrees of freedom and *P* value noted<br>*Give P values as exact values whenever suitable.*
☒	☐	For Bayesian analysis, information on the choice of priors and Markov chain Monte Carlo settings
☒	☐	For hierarchical and complex designs, identification of the appropriate level for tests and full reporting of outcomes
☒	☐	Estimates of effect sizes (e.g. Cohen's *d*, Pearson's *r*), indicating how they were calculated
☐	☒	Clearly defined error bars<br>*State explicitly what error bars represent (e.g. SD, SE, CI)*

Our web collection on statistics for biologists may be useful.

Software and code

Policy information about availability of computer code

Data collection	There was no common software used and no special code developed, but each group used their own custom softwares. The protocol gave enough detail that the results did not depend on the particular software used, one of the strengths of this study.
Data analysis	A major part of this manuscript is to describe the data analysis in great detail. This is independent of the software used, one of the main outcomes of this study.

For manuscripts utilizing custom algorithms or software that are central to the research but not yet described in published literature, software must be made available to editors/reviewers upon request. We strongly encourage code deposition in a community repository (e.g. GitHub). See the Nature Research guidelines for submitting code & software for further information.

Data

March 2018

Policy information about availability of data

All manuscripts must include a data availability statement. This statement should provide the following information, where applicable:

- Accession codes, unique identifiers, or web links for publicly available datasets
- A list of figures that have associated raw data
- A description of any restrictions on data availability

All data is available upon request fom the authors. The data of figure 2 is available on Zenodo at: http://doi.org/10.5281/zenodo.1249497.

Field-specific reporting

Please select the best fit for your research. If you are not sure, read the appropriate sections before making your selection.

☒ Life sciences ☐ Behavioural & social sciences

For a reference copy of the document with all sections, see nature.com/authors/policies/ReportingSummary-flat.pdf

Life sciences

Study design

All studies must disclose on these points even when the disclosure is negative.

Sample size	We invited 22 labs and 20 labs finally responded. All resulting uncertainties are given, they take the sample size into account. Finally, similiar results were obtained for different samples measured by a different number of labs (not all labs could measure all samples).
Data exclusions	No data was excluded from the analysis.
Replication	Each lab was asked to perform experiments according to standards used in the lab. Reproducibility in this respect is already given by the multi-laboratory approach used in this study.
Randomization	The samples were randomly allocated to the various labs
Blinding	Data collection and analysis was blind

Materials & experimental systems

Policy information about availability of materials

n/a	Involved in the study
☒	☐ Unique materials
☒	☐ Antibodies
☒	☐ Eukaryotic cell lines
☒	☐ Research animals
☒	☐ Human research participants

Method-specific reporting

n/a	Involved in the study
☒	☐ ChIP-seq
☒	☐ Flow cytometry
☒	☐ Magnetic resonance imaging
